# Characterization of silk genes in *Ephestia kuehniella* and *Galleria mellonella* revealed duplication of sericin genes and highly divergent sequences encoding fibroin heavy chains

**DOI:** 10.3389/fmolb.2022.1023381

**Published:** 2022-11-29

**Authors:** Bulah Chia-hsiang Wu, Ivo Sauman, Houda Ouns Maaroufi, Anna Zaloudikova, Martina Zurovcova, Barbara Kludkiewicz, Miluse Hradilova, Michal Zurovec

**Affiliations:** ^1^ Biology Centre of the Czech Academy of Sciences, Institute of Entomology, Ceske Budejovice, Czechia; ^2^ Faculty of Science, University of South Bohemia, Ceske Budejovice, Czechia; ^3^ Institute of Molecular Genetics, Academy of Sciences of the Czech Republic, Praha, Czechia

**Keywords:** synteny, mucin, mediterranean flour moth, wax moth, pyralidae, crambidae

## Abstract

Silk is a secretory product of numerous arthropods with remarkable mechanical properties. In this work, we present the complete sequences of the putative major silk proteins of *E. kuehniella* and compare them with those of *G. mellonella*, which belongs to the same moth family Pyralidae. To identify the silk genes of both species, we combined proteomic analysis of cocoon silk with a homology search in transcriptomes and genomic sequences to complement the information on both species. We analyzed structure of the candidate genes obtained, their expression specificity and their evolutionary relationships. We demonstrate that the silks of *E. kuehniella and G. mellonella* differ in their hydrophobicity and that the silk of *E. kuehniella* is highly hygroscopic. In our experiments, we show that the number of genes encoding sericins is higher in *G. mellonella* than in *E. kuehniella*. By analyzing the synteny of the chromosomal segment encoding sericin genes in both moth species, we found that the region encoding sericins is duplicated in *G. mellonella*. Finally, we present the complete primary structures of nine *fibH* genes and proteins from both families of the suborder Pyraloidea and discuss their specific and conserved features. This study provides a foundation for future research on the evolution of silk proteins and lays the groundwork for future detailed functional studies.

## Introduction

The Pyraloidea are the third largest superfamily of the Ditrysian Lepidoptera order, containing about 16,000 species. They are found on all continents except Antarctica and consist of two families: Pyralidae and Crambidae. About 5,000 representatives of the Pyralidae family have been described, including a number of important pests. The Mediterranean flour moth, *E. kuehniella* Zeller (Lepidoptera: Pyralidae), is one of the most important pests of stored products. The larvae infest stocks of flour or cereal grains as a food source, but they do the most damage by producing silk that clogs machinery (Jacob and Cox, 1977). The larvae spend most of their lives in silk tubes that provide protection from parasitoids and reduce water loss (Fedic et al., 2003). *G. mellonella* (also from the family Pyralidae) is a pest of honey bees whose larvae live in hives protected by a maze of feeding tubes (Ellis and Hayes, 2009). Previous studies of *G. mellonella* helped to elucidate that the general protein composition of silk is conserved in moths (Zurovec et al., 1992; Zurovec et al., 1995; Zurovec et al., 1998). The study of sericin genes in *G. mellonella* also revealed that there is a high proportion of proteins surrounding the fibroin core, which is associated with an unusually high number of sericin genes (Kludkiewicz et al., 2019).

The silk of lepidoptera is produced in the transformed labial salivary gland of the larva, which is called the silk gland (SG). The posterior silk gland (PSG) produces filaments consisting of three proteins: fibroin heavy chain, fibroin light chain and fibrohexamerin (Shimura et al., 1982), while the middle silk gland (MSG) produces envelope proteins that mainly have an adhesive function and primarily consist of sericins (Gamo, 1982; Prudhomme et al., 1985). The anterior silk gland (ASG) is a tubular duct lined by a cuticle. The silk undergoes significant changes during evolution, both in the sequence of individual proteins and in the presence of individual protein components.

The fibroin heavy chain (FibH) is the best-studied silk component. It contains regions of regular protein secondary structures consisting of antiparallel beta-sheets and forms crystalline domains responsible for fiber strength (Deny, 1980). These tend to be primarily composed of the simple amino acids alanine, glycine, and serine, which enable the formation of beta structures (Craig, 1997). Despite the profound sequence differences between species, there are structural requirements needed for fiber strength, and a limited number of *ß*-sheet configurations for suitable crystal domain motifs exist. This may have led to convergent evolution and the reappearance of motifs found in unrelated species (Lucas and Rudall, 1968). Previous experiments have shown that the silk fibers of *E. kuehniella* and *G. mellonella* have approximately the same tensile strength as those of *B. mori*, although both contain relatively short and scattered putative crystallites in the FibH (Fedic et al., 2003). Proteins produced by MSG are less well studied and seem to be subject to even greater changes than fibroins. These include large adhesive proteins, sericins, mucins and zonadhesin-like proteins, as well as seroins and protease inhibitors involved in protection against microorganisms. The low conservation of silk gene sequences makes it difficult to identify new proteins based on homology between more distant lepidopteran species; however, homology can be very useful for identifying genes in closely related species. Recent advances in proteomics and sequencing of lepidopteran genomes have provided a flood of information on new silk components and have made it possible to obtain complete sequences of large repetitive genes that were previously difficult to study (Davey et al., 2021).

In this study, we present the complete sequences of the putative major silk proteins from two members of the moth family Pyralidae, *E. kuehniella* (subfamily Phycitinae) and *G. mellonella* (subfamily Galleriinae). We identified silk genes in both moths based on proteomic analysis of cocoon silk and by searching for homologies in the transcriptomes and genomes of both species. We also compared genomic sequences of *E. kuehniella* and *G. mellonella* with genomic DNA from *Amyelois transitella* (subfamily Phycitinae), also from the Pyralidae family. We discovered a region containing clusters of sericin genes and identified blocks of synteny (colocalized gene clusters shared between genomes). The resulting microsynteny map allowed identification of duplication events in the sericin family. Finally, we present the complete primary structures of nine FibH proteins from both families of the suborder Pyraloidea and discuss their specific and conserved features.

## Materials and methods

### Insects and silk

Mediterranean flour moth (*E. kuehniella*) and waxmoth (*G. mellonella*) larvae were laboratory strains previously established from specimens found in České Budějovice, Czech Republic and kept in the Institute of Entomology, Biology Centre of the Czech Academy of Sciences. The *E. kuehniella* larvae were reared on a mixture of wheat flour and wheat bran (volume ratio of 4:1) supplemented with a small amount of dry yeast at 24°C without humidity control. The food was sterilized at 110°C for 2 h before adding the yeast (Marec and Traut, 1994). The *G. mellonella* larvae were reared on a semi-artificial diet at 30°C (Sehnal, 1966). The diet for *G. mellonella* consisted of wheat flower, corn and wheat meals in ratios 1:2:1 mixed with dry milk, dry yeast, beeswax, glycerol, and honey. *B. mori* cocoons were a gift from Dr. D. Zitnan, (Bratislava, Slovakia).

### Histology and electron microscopy

Whole mount preparations of SGs from *E. kuehniella* were conducted as follows: SGs were dissected from water anesthetized last instar wandering stage larvae, transferred to a drop of phosphate-buffered saline (PBS) on a microscope slide, covered with a coverslip, and imaged under an Olympus BX63 microscope (Olympus Corporation, Tokyo, Japan) equipped with a CCD camera (Olympus DP74).

The histology of *E. kuehniella* larvae was carried out as follows: The cuticles of water anesthetized larvae were punctured with a fine needle under Bouin–Hollande fixing solution supplemented with mercuric chloride (Levine et al., 1995). After one hour of fixation, the larvae were cut into three parts and then fixed overnight at 4°C. Standard histological procedures were used for tissue dehydration, embedding in Paraplast, sectioning (7 μm), deparaffinization, and rehydration. Sections were treated with Lugol’s iodine followed by 7.5% sodium thiosulfate solution to remove residual heavy metal ions, washed in distilled water, and stained with the HT15 Trichrome Staining Kit (Masson) (Sigma-Aldrich, Inc., St. Louis, MO, United States) according to the manufacturer’s protocol. The stained sections were dehydrated and mounted using a DPX mounting medium (Fluka, Buchs, Switzerland). High-resolution images were acquired using a BX63 microscope, DP74 CMOS camera, and cellSens software (Olympus) by stitching multiple images together.

Semi-thin sections of cocoons were produced as follows: Pieces of freshly spun and degummed cocoons were prepared in PBS and fixed in 2.5% glutaraldehyde or at least 4 h at room temperature (RT) or overnight at 4°C. Specimens were then dehydrated and embedded in Epon resin as previously described (Kludkiewicz et al., 2019). Semi-thin sections were cut with a glass knife and placed onto a droplet of 10% acetone on a microscope slide. The dried sections were stained with toluidine blue and imaged under a light microscope.

The analysis of the ultrastructure of the silk was conducted as follows: Silk samples were cut from cocoons, glued to the surface of aluminum holders, sputter-coated with gold, and analyzed using a Jeol JSM-7401F scanning electron microscope (Jeol, Akishima, Japan).

### Northern blotting and qPCR

Total RNA was extracted from dissected larvae and SG with TRIzol reagent (Invitrogen). RNA aliquots of 5 µg were collected for agarose electrophoresis, blotted onto a nylon membrane (Hybond N+, Sigma-Aldrich, St. Louis, United States), and hybridized under high stringency conditions as previously described (Zurovec et al., 2016). Probes for northern blotting were amplified using reverse transcription polymerase chain reaction and primers listed in Supplementary Table S1A, then labeled with *a*-^32^P[dATP] using random priming with an Oligo labeling kit (Thermo Fisher Scientific, Prague). Autoradiographic detection was performed using the storage phosphor screen of a STORM 860 Phosphorimager (Molecular Dynamics, Chatsworth, United States).

qPCR was performed using HOT FIREPol EvaGreen qPCR Mix Plus (Solis BioDyne, Tartu, Estonia). Five individuals were used for each sample. All samples were collected in triplicate. The PCR reaction volume of 20 µL contained 5 µL of diluted cDNA and 250 nM primers. Amplification was carried out using a Rotor-Gene Q MDx 2plex HRM (Qiagen, Hilden, Germany) for 45 cycles (95°C for 15 s; annealing temperature adjusted to the primer pair for 30 s; 72°C for 20 s) following an initial denaturation/Pol activation step (95°C for 15 min). Each sample was analyzed in triplicate. Primers (Supplementary Table S1B) were designed using Geneious Prime software platform (Biomatters, Auckland, New Zealand; version 2021.2.2) to ensure that each amplicon was specific. The output was analyzed using the software Rotor Gene Q (version 2.3.5). Elongation factor 1 alpha (EF1a, NM_001044045.1) was used as a reference gene, and the relative expression of the target genes was calculated using the 2^−ΔΔCT^ method (Livak and Schmittgen, 2001). Statistical analysis was performed using the Student’s t-test in R (version 4.1.1); *p*-values < 0.05 were considered statistically significant. The detailed statistical analysis is shown in Supplementary Table S2.

### Transcriptome preparation

RNA isolation, cDNA library synthesis, and RNA sequencing were performed as previously described (Rouhova et al., 2021). Briefly, last instar wandering larvae were dissected and tissues were separated. The RNA for transcriptome preparation was isolated using TRIzol reagent and further purified using a NucleoSpin RNA II kit (Macherey-Nagel, Duren, Germany). The mRNA was then isolated using Oligo(dT)25 Dynabeads (Ambion, Life Technologies). RNA integrity was checked, and concentration was measured using a Bioanalyser 2100 (Agilent, Waldbronn, Germany). The cDNA library was prepared using a NEXTflex Rapid RNA-Seq Kit (Bioo Scientific, Austin, TX, United States). Sequencing was performed using a MiSeq (Illumina, San Diego, United States), generating sequences in a 2 × 150 nt pair-end format. The BUSCO tool suite (version 3.0) (Simao et al., 2015) was used to assess the completeness of the assembly. A total of 1.6 × 10^7^ reads were assembled *de novo* using Trinity software (version 2.9.1 + galaxy1) on the Galaxy platform (Afgan et al., 2018). The transcriptome was further improved with the genome annotation pipeline MAKER (version 2.28) by incorporating information on the *E. kuehniella* genome assembly (Visser et al., 2021), as well as protein datasets for *B. mori*, *G. mellonella*, and arthropoda (Odb10, https://busco.ezlab.org/). Full-length transcripts were found using the genome annotation pipeline MAKER (version 2.28). The completeness of the resulting transcriptome assemblies was assessed using BUSCO (version 5.2.2, lineage dataset insecta_odb10). Transcripts were annotated using NCBI BLAST (version 2.12.0+), InterProScan (version 5.52–86.0), and Pepstats/Pepinfo from EMBOSS (version 6.5.7).

The transcriptome of *G. mellonella* was previously prepared (Kludkiewicz et al., 2019) using Roche GS-FLX 454 pyrosequencing according to the manufacturer’s instructions. Three cDNA libraries were prepared: those from the SGs of the penultimate-instar larvae (PI), the post-feeding wandering last instar larvae (WS), and the apolyzing (initial phase of pupation) last instar larvae (ECD). These were then sequenced, and the data were concatenated.

### Chromosomal localization and collinearity analyses

We used high-quality genome sequences of *E. kuehniella* (Visser et al., 2021) and *G. mellonella* (GenBank assembly accession GCA_003640425.2). Transcripts of *E. kuehniella* and *G. mellonella* silk genes were mapped to the genomes using minimap2 (version 2.24-r1122) to locate potential gene clusters (Li, 2018). Syntenic relationships were then built based on reciprocal best translated blast (tblastx, version 2.12.0+) hits among the transcriptome datasets of *E. kuehniella*, *G. mellonella*, *A. transiella* (GenBank assembly accession GCA_001186105.1), and *B. mori* (GCA_014905235.2) and visualized using R package ggplot2 (Wickham, 2009).

### Silk degumming and hygroscopicity tests

To determine the percentage of soluble silk components, cocoon samples containing approximately 40 mg of dried *E. kuehniella* and *G. mellonella* silk material were cut into pieces, weighed, submerged in water, and boiled three times for 15 min. The samples were then centrifuged, and the soluble fraction was discarded. The undissolved silk remaining in the pellet was washed five times with water, vacuum dried, and weighed. The soluble fraction was measured by calculating the weight loss percentage before and after the degumming process as described previously (Kludkiewicz et al., 2019).

To measure the hygroscopicity of silk, cocoon samples approximately 40 mg each of *E. kuehniella*, *G. mellonella* and *B. mori* were vacuum dried, weighed, and then incubated in a jar with 75% humidity for 48 h. Moisture uptake was inferred from the percentage increase in the sample weight before and after the incubation. Six biological replicates were used for every sample. Statistical significance was tested by the Student’s t-test in R (version 4.1.1).

The grand average of hydropathicity index (GRAVY) of the FibH was calculated using the ExPASy ProtParam server (https://web.expasy.org/protparam/) from the sum of the hydropathy values of all amino acids divided by the sequence length (Kyte and Doolittle, 1982).

### Protein identification using mass spectrometry

Silk samples were dissolved in 8 M urea and further processed with SP3 as previously described (Hughes et al., 2014). After washing the samples, they were digested with trypsin, acidified with trifluoroacetic acid to a final concentration of 1%, and peptides were desalted with homemade C18 disk-packed tips (Empore, Oxford, United States) according to Rappsilber et al. (Rappsilber et al., 2007).

The samples were processed and analyzed using nanoscale liquid chromatography coupled to tandem mass spectrometry (nLC-MS/MS) as described elsewhere (Erban et al., 2020). The analysis and quantification of proteins were performed using MaxQuant label-free algorithms (MaxQuant, version 1.5.3.8) (Cox et al., 2014). The false discovery rate (FDR) was set to 1% for both proteins and peptides, and a minimum length of seven amino acids was set. The Andromeda search engine (Cox et al., 2011) was used to compare the MS/MS spectra with the transcriptome/genome-derived *E. kuehniella* protein database. Further data analysis was performed using Perseus 1.5.2.4 software (Tyanova et al., 2016).

### Phylogenetic analysis

Coding sequences identified in the annotated genomes were used. Codon-based alignment was performed using MEGA7 software according to the MUSCLE method (Kumar et al., 2016). The phylogram was generated using the IQ-TREE server (Nguyen et al., 2015), which included both the selection of the best substitution model by ModelFinder (Kalyaanamoorthy et al., 2017) and tree inference using MLE (ultrafast bootstrap, 1,000 replicates).

### Identification of *fibH, ser1* and *muc1* genes in other pyraloidea

*G. mellonella* FibH sequence was inferred from two long-read sequencing genome assemblies. The FibH sequences of *Acentria ephemerella, Acrobasis suavella, Chilo suppressalis, Cnaphalocrocis exigua, Endotricha flammealis, Hypsopygia costalis and Plodia interpunctella* were identified from high-quality genomes published by the Darwin Tree of Life Project (Blaxter and Project, 2022).

We identified *fibH* genes from these assemblies using TBLASTN and conserved N- and C-termini as query sequences. Fibroin sequences were predicted from the surrounding sequence using online Augustus (Stanke and Morgenstern, 2005). The software BioEdit (v 7.2) was used to visualize sequence alignments (Hall, 1999). Information on accession numbers of genome assemblies and sequences was shown in Table 3. We also used TBLASTN and conserved C-termini of Ser1 and Muc1 as query sequences.

## Results

### *E. kuehniella* silk and silk glands

**FIGURE 1:**
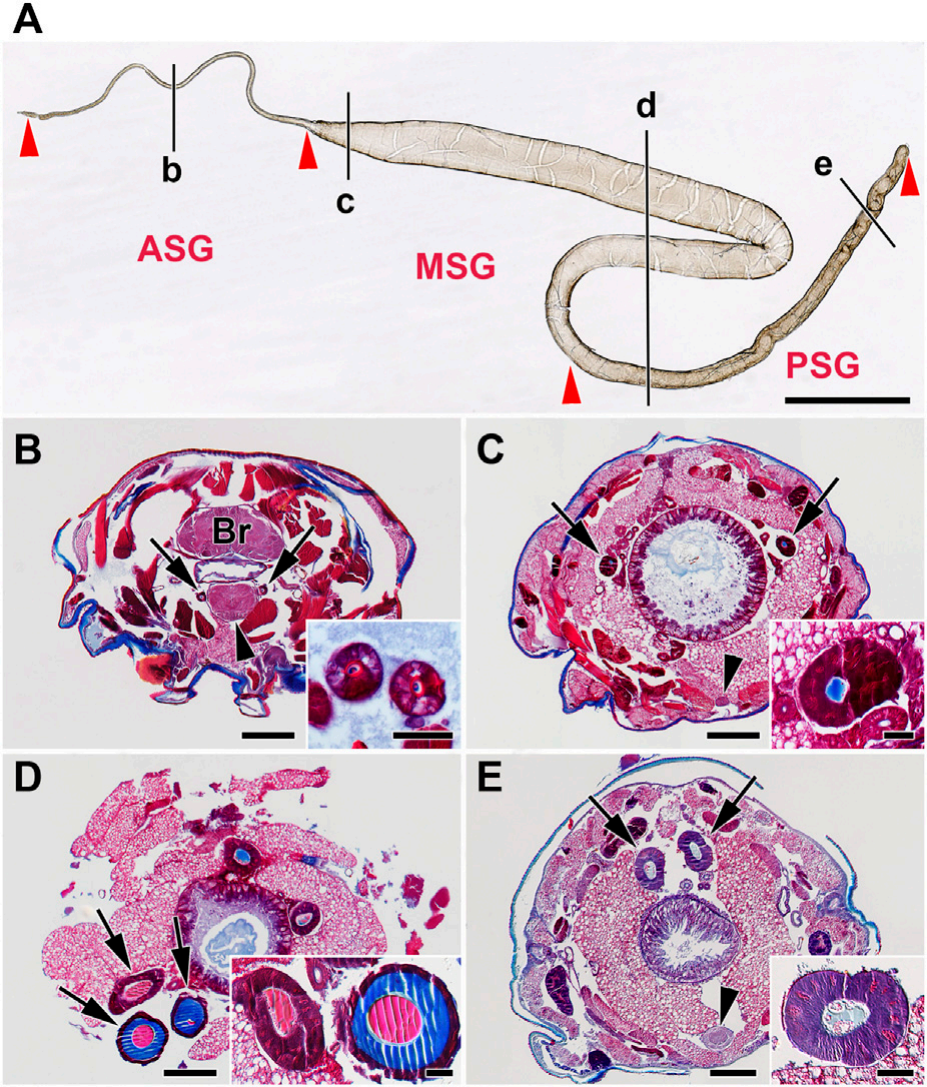
Morphology of the silk gland (SG) from *E. kuehniella* last instar larvae. **(A)** Whole mount preparation of one SG illustrates its overall morphology. Red arrowheads depict the boundaries of the SG compartments, where ASG = anterior SG, MSG = middle SG, and PSG = posterior SG. Black lines marked by the lowercase letters b–e refer to the whole-body sections B–E and show the approximate positions where the glands were cut in transverse Paraplast sections. **(B–E)** Transverse Paraplast sections through the body of the last larval instar stained with Masson trichrome stain (Sigma). The inset images show higher magnification of the SG sections marked by arrows. **(B)** ASG; brain (Br); the arrowhead depicts the suboesophageal ganglion (SOG). **(C)** Anterior portion of the MSG; arrowhead shows ventral nerve cord. **(D)** MSG in the region of the sigmoidal loop. **(E)** PSG; the arrowhead marks the ventral nerve cord ganglion. Red areas are acidic; blue areas are alkaline. Scale-bars: **(A)**, 1,000 μm; **(B–E)**, 200 μm; inset images, 50 μm.

The SG of *E. kuehniella* consists of a tube with large polyploid secretory cells. Like in other moth species, three regions can be distinguished morphologically: anterior, middle, and posterior (Figure 1A). The PSG is approximately 20% shorter than the MSG and is not folded. The SG extends about two-thirds of the length of the larval body. The ASG is narrow and gradually widens into the MSG. A large sigmoid loop forms at the junction between the MSG and the PSG. The diameter of the MSG remains more or less the same and decreases only slightly toward the PSG. The boundary between the rear part of the MSG and the PSG is less distinct than in *G. mellonella* or *B. mori.*

**FIGURE 2:**
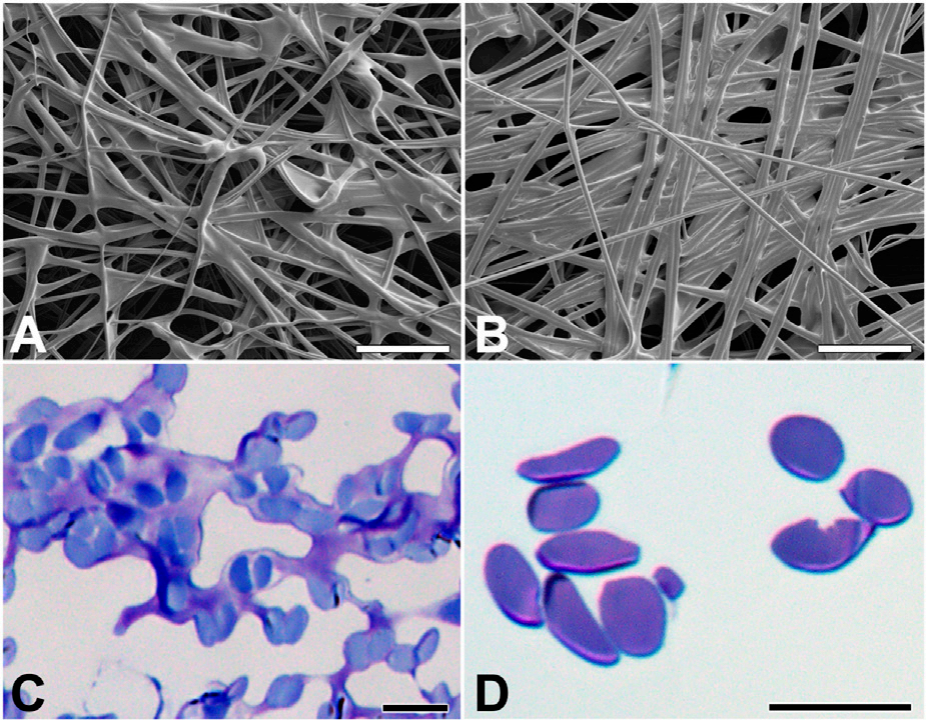
Silk of *E. kuehniella* cocoon. **(A,B)** Scanning electron micrographs of the outer and inner surfaces and inner surfaces of the cocoon, respectively. **(C,D)** Toluidine blue stained semi-thin sections of the silk fibers of the cocoon before and after degumming, respectively. Scale bars: A,B = 50 μm; C,D = 10 μm.

Silk fiber width varies among moth species, ranging from 12 μm in *B. mori* to 5 μm in *G. mellonella* and 0.5–1 μm in diameter in *Tineola bisselliella* (Kludkiewicz et al., 2019; Rouhova et al., 2021). To study the morphology of the silk cocoons and fibers, we characterized them using a scanning electron microscope (Figures 2A,B). The width of silk fiber of *E. kuehniella* is approximately 1 μm. The overall structure of the silk of *E. kuehniella* appears to be similar to that of *G. mellonella*.

Cells with polyploid nuclei are found along the entire length of the gland. The gland only produces fibroins in the PSG, and these are thought to be mixed with sericins and other silk components in the MSG and ASG. As can be seen on the paraffin sections stained with Masson trichrome stain, there are color differences in the liquid silk in the different glandular compartments. At least two types of secretion are seen on the glandular sections: a column of fibroin surrounded by a layer of sticky sericins (the color changes in the stained sections). While the fibroin in the PSG was stained blue, both the fibroin and sericin in the MSG and ASG were red (Figures 1B–E).

Silks differ in sericin content from 26% in *B. mori* to 48% in *G*. *mellonella* (Kludkiewicz et al., 2019). To compare the percentage of these proteins in the silks, we dissolved the coating proteins of *E. kuehniella*, *G. mellonella*, and *B. mori* by degumming the silk in water, which dissolved and removed most of the sericin layer. The removal of sericins was verified by microscopic examination. Interestingly, the degumming dissolved also part of the silk core of *E. kuehniella* (Figures 2C,D). Therefore, we concluded that the silk of *E. kuehniella* is more soluble than that of *G*. *mellonella* and, therefore, that this method is not suitable for measuring the exact proportion of sericins in *E. kuehniella* silk.

**FIGURE 3:**
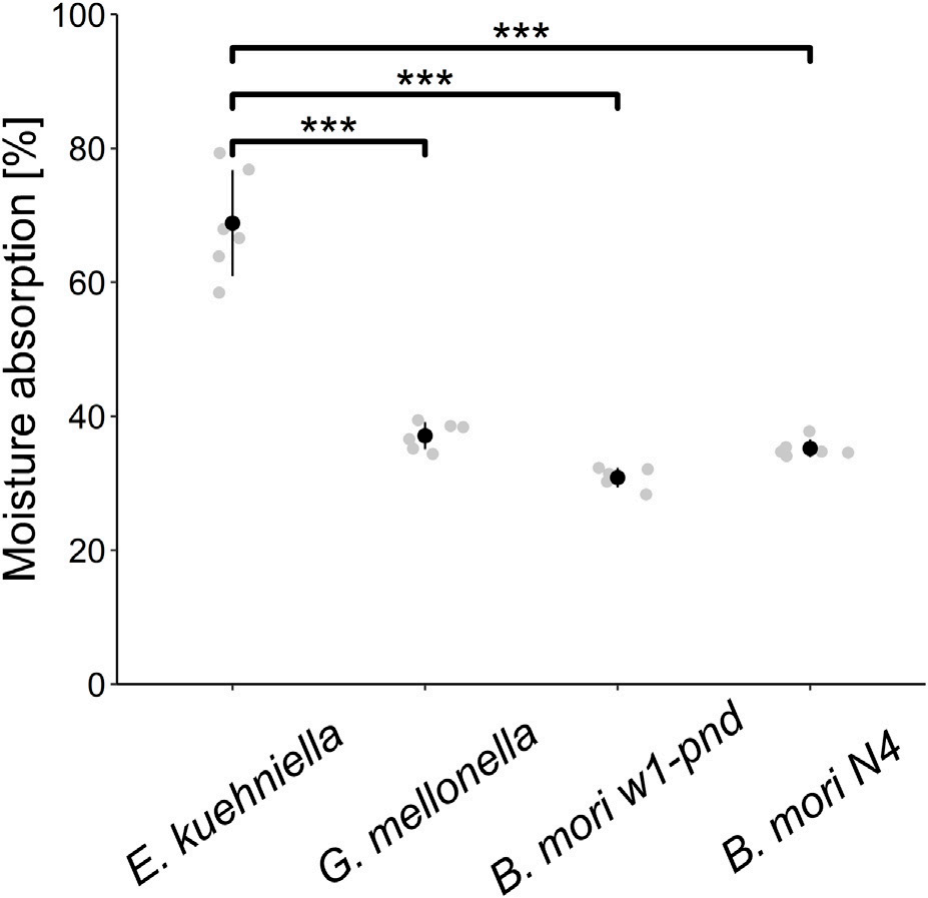
Hygroscopicity evaluation. The scatter plot shows the average amounts of water absorption in the dehydrated cocoon silk of *E. kuehniella*, *G. mellonella*, and *B. mori* *w1-pnd* and *N4* strains. Black dots represent the mean, and vertical bars represent the mean ± SD. *** indicates *p* < 0.001 (paired *t*-test comparing means, *n* = 6).

Because the properties of different silk species can be distinctive with respect to water, such as solubility or even hygroscopicity, we also tested water adsorption from the environment. As can be seen in Figure 3, *E. kuehniella* silk is highly hygroscopic compared to *G. mellonella* and *B. mori* silks, and it can retain twice as much water as *G. mellonella* silk (68% and 30%, respectively; Figure 3).

### Transcriptome *de novo* assembly

The first *de novo* assembly of the silk gland-specific transcriptome of *E. kuehniella* revealed 43,923 contigs with an average length of 1,012.7 base pairs. The completeness of the non-redundant transcripts was assessed using the BUSCO tool suite. The results showed that the transcriptome was 81.6% complete. However, approximately 25% of the complete and duplicated BUSCOs indicated redundant isoforms, and approximately 19% of the incomplete BUSCOs (8% fragmented and 10.4% missing) indicated missing genes. To address this issue, we took advantage of the long-read genome assembly of *E. kuehniella* (Visser et al., 2021) and used a combined transcriptome assembly strategy to generate an improved transcriptome. The MAKER-annotated genome contained a total of 13,382 recovered protein-coding genes with an average gene length of 7,207.8 base pairs. The BUSCO statistics showed a completeness of 98.6%, while the levels of redundancy and incompleteness decreased to 0.5% and 1.4%, respectively. We concluded that this improved transcriptome contained high-quality data suitable for further analysis (see Supplementary Table S3).

### Detection of *E. kuehniella* candidate silk proteins

Sequence annotation revealed that a substantial proportion of cDNAs encode ribosomal proteins or proteins involved in protein translation or transport. Potentially secreted proteins identified by the presence of a putative signal peptide accounted for approximately 10% of all annotated contigs. Because silk genes evolve rapidly, it is difficult to identify them in new moth species based on homology without information on genes from a closely related species. In this way, we were able to reliably identify the sequences of the FibH, the fibroin light chain, and P25/fibrohexamerin (P25) based on homology to known genes.

Because *E. kuehniella* silk was available to us, we chose proteomic analysis as the primary method for detecting the gene sequences that encode its components. The proteins of a silk cocoon were dissolved in urea and trypsinized, and the resulting peptides were analyzed using proteomic analysis. The MS/MS spectra of the peptides were aligned with the protein sequence database derived from the reference transcriptome. It was expected that most of the proteins detected in the silk would not be structural components because some of the housekeeping proteins are secreted from SG cells *via* apocrine-like secretion during silk spinning. We identified 140 proteins, 77 of which contained a predicted signal peptide sequence. BLAST-based annotations were performed using the NCBI nr database, and the annotations were manually verified. Based on the annotations, we excluded most proteins with close homologs in other moth species that were not associated with silk structure.

### Expression specificity of candidate *E. kuehniella* proteins

**FIGURE 4:**
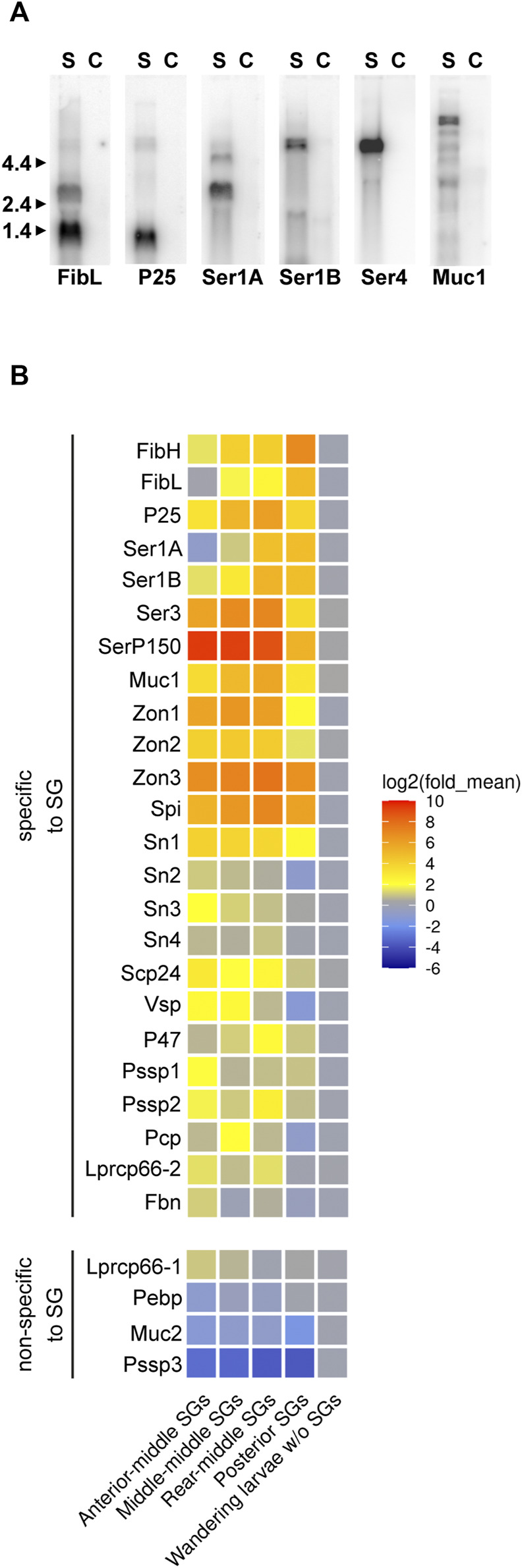
Expression of selected silk genes detected by **(A)** northern blotting and **(B)** qPCR. **(A)** Lanes: C – larva without SG, S – larval SG. Total RNA (5 μg) was separated on an agarose gel, blotted to a nylon membrane and probed with [^32^P]-labelled cDNA fragments from each of the indicated genes. The length (kb) of the size marker is indicated on the left side. **(B)** Relative expression of candidate tissue-specific genes in controls and parts of silk glands of last instar larvae examined *via* qPCR. mRNA expression levels were normalized to the internal reference gene elongation factor 1-α. Heatmap was plotted based on log2-transformed fold change between SG and control, indicated by the colored scale. Genes of significantly higher expression level in SG (*p* < 0.05) were classified as SG-specific genes (see Supplementary Table S2 for statistics). Gene names are shown in Table 1.

Putative structural silk proteins are likely to be abundant. They carry signal peptides at their N-terminal sequences, and their transcripts are specific to the SG. We isolated total RNA from control larvae with ablated SGs, as well as from different parts of the SG. The SG specificity of the candidate transcripts encoding silk proteins was confirmed using northern blotting and qPCR analyses. The northern blotting analysis revealed that some candidate genes could produce more bands, suggesting alternative splicing (Figure 4A). Most of the transcripts showed distinct differences in expression in SG sub-regions. For example, *Ek-serP150* is highly expressed in the anterior part of the MSG, whereas the *Ek-Zon1* and *Ek-Ser1A* transcripts are predominantly expressed in the rear MSG (Figure 4B). Interestingly, maximal expression of the *Ek-P25* transcript was found in the rear MSG.

### Comparison of candidate silk proteins between *E. kuehniella and G. mellonella*

To identify a complete set of candidate silk-encoding genes of *E. kuehniella*, we performed a parallel study on *G. mellonella.* Previous results on the silk of *G. mellonella* were supplemented with a new cocoon protein proteomic analysis. The tryptic peptides were tested against the custom protein database derived from the NCBI dataset (GenBank assembly accession GCA_003640425.2) and the previously created transcriptome (Kludkiewicz et al., 2019). The resulting set of *G. mellonella* sequences was used to search for homologous sequences in the transcriptome of *E. kuehniella* and *vice versa*. Data for both species were then complemented based on homology. Thus, BLAST searches of the *G. mellonella* sequences of P-12 (LOC113521678), P13 (LOC113521978), mucin-5AC-like (LOC113516440), seroin 2 (LOC113518101) zonadhesin (LOC113516017), and candidate silk proteins (LOC113523011, LOC113515440, and LOC113511581) revealed new putative *E. kuehniella* homologs that were not detected *via* the proteomic approach (Table 1). Conversely, we discovered homologs of *E. kuehniella* proteins annotated as fibrillin (OP185491), pupal cuticle protein-like (OP185495), and phosphatidylethanolamine-binding protein (OP185492) in the *G. mellonella* transcriptome (not found in *G. mellonella* silk proteomics). In addition, the new proteomic analysis of *G. mellonella* cocoon silk revealed 18 silk protein candidates that were not previously detected in silk, including several sericins, mucin, zonadhesin, seroin, and cuticle proteins (Table 2).

**TABLE 1:** Major silk proteins in *E. kuehniella* cocoons. GenBank – GenBank accession numbers; Intensity – MaxQuant peptide intensity; M. W. – molecular weight (kDa); pI – isoelectric point; H. I. – hydropathy index (GRAVY); 1^st^/2^nd^/3^rd^ AA (%) – proportion of the three most frequent amino acids; Evid. – data to detect/infer proteins, where N, P, Q, and T represent northern blotting, proteome, qPCR, and transcriptome.

Gene/protein	GenBank	Intensity	Evid.	M. W.	pI	H. I.	1st AA (%)	2nd AA (%)	3rd AA (%)
Ek-Ser3	ON604819	8093000000	PQ	144.19	6.865	−0.823	S (52.6)	G (16.2)	T (8.3)
Ek-FibH	ON604816	5058700000	PQ	493.70	3.491	0.054	S (23.1)	G (22.0)	A (19.1)
Ek-FibL	ON604822	4001400000	NPQ	26.41	4.186	0.299	A (20.1)	G (11.0)	N (10.2)
Ek-Zon1	ON604824	3562800000	PQ	140.56	4.717	−0.542	C (15.1)	P (9.3)	G (8.6)
Ek-P25	ON604823	3071800000	NPQ	25.02	7.334	−0.076	L (8.7)	F (8.3)	N (8.3)
Ek-SerP150	ON604820	2846400000	PQ	173.26	4.212	−0.849	S (34.6)	Q (11.1)	G (9.2)
Ek-Muc1	ON604821	2764400000	NPQ	473.65	4.044	−1.378	Q (25.7)	S (23.5)	E (11.0)
Ek-Zon2	ON604825	1061200000	PQ	101.09	4.907	−0.235	C (8.9)	S (7.8)	N (7.3)
Ek-Ser1A	ON604817	231370000	NPQ	124.10	4.196	−0.545	T (19.7)	S (17.6)	A (13.1)
Ek-Lprcp66-1	ON604829	112370000	PQ	18.28	6.503	0.236	G (22.5)	A (16.6)	L (10.7)
Ek-Ser1B	ON604818	76048000	NPQ	149.79	4.068	0.032	A (17.3)	S (14.6)	P (13.5)
Ek-Sn1	ON604827	58061000	PQ	29.50	4.461	−0.603	P (13.9)	E (9.8)	V (9.8)
Ek-Lprcp66-2	ON604830	27834000	PQ	20.43	7.358	0.085	A (21.8)	G (18.9)	Y (11.7)
Ek-Zon3	ON604826	17266000	PQ	48.74	4.119	−0.431	P (11.0)	S (10.4)	C (9.7)
Ek-Sn4	ON604828	9657900	PQ	12.44	6.792	−0.323	A (11.3)	G (10.4)	N (9.3)
Ek-Sn3	OP185489	7321500	PQ	11.63	4.532	−0.679	E (10.4)	N (10.4)	S (10.4)
Ek-P47	OP185490	5865900	PQ	31.90	4.528	−0.647	E (12.0)	K (9.1)	N (8.3)
Ek-Pebp	OP185492	4880600	PQ	23.00	9.368	−0.111	V (11.3)	A (10.8)	P (8.5)
Ek-Fbn	OP185491	3261600	PQ	153.14	5.147	−0.797	P (10.8)	T (8.1)	E (8.0)
Ek-Pcp	OP185495	2008500	PQ	26.85	6.502	−0.574	A (28.0)	Q (14.2)	P (9.4)
Ek-Muc2	OP185487	764630	P	40.22	3.388	−0.866	T (36.9)	E (16.6)	S (9.1)
Ek-Csp1	OP185497	-	T	58.41	6.619	−1.298	S (10.9)	Q (9.9)	N (9.1)
Ek-Csp2	OP251358	-	T	31.29	7.353	−0.789	P (11.9)	S (8.6)	D (7.6)
Ek-Csp3	OP251359	-	T	44.91	4.582	−0.369	V (10.4)	P (10.1)	S (9.4)
Ek-Muc3	OP185493	-	T	85.32	8.361	−0.914	T (15.0)	A (11.4)	P (9.9)
Ek-P-12	OP185496	-	T	16.82	7.047	−0.625	G (39.5)	Q (9.6)	F (7.9)
Ek-P13	OP185503	-	T	10.68	9.988	−0.458	P (14.3)	V (10.2)	A (9.2)
Ek-Ser4	OP251360	-	NT	48.72	4.455	−0.613	S (40.6)	G (9.2)	A (8.4)
Ek-Sn2	OP185488	-	QT	24.71	6.226	−0.450	S (12.0)	N (10.2)	F (7.8)
Ek-Zon4	OP185494	-	T	22.19	7.112	−0.536	C (14.8)	P (10.8)	G (7.9)

**TABLE 2:** Major silk proteins in *G. mellonella* cocoon. GenBank accession numbers; Intensity – MaxQuant peptide intensity; M. W. – molecular weight (kDa); pI – isoelectric point; H. I. – hydrophobicity index (GRAVY); 1^st^/2^nd^/3^rd^ AA (%) – proportion of the three most frequent amino acids; Evid. – protein newly identified in this paper (A) or previously identified in (Kludkiewicz et al., 2019) (B).

Gene/protein	Genbank	Intensity	Evid.	M. W.	pI	H. I.	1st AA (%)	2nd AA (%)	3rd AA (%)
Gm-FibL	XM_026895755	22648000000	AB	27.01	3.994	0.352	A (19.1)	L (9.7)	N (9.4)
Gm-FibH	XM_026905081	20827000000	AB	487.83	3.723	0.553	G (31.3)	A (23.3)	S (17.6)
Gm-P250	XM_031911780	18581000000	AB	56.27	11.222	−0.564	S (34.0)	G (10.6)	P (9.9)
Gm-Muc4-L	MG770312	15626000000	AB	174.28	4.301	−0.883	S (19.7)	Q (11.9)	E (9.7)
Gm-P25	XM_026894481	11985000000	AB	24.84	5.301	−0.047	L (11.0)	N (10.6)	A (8.3)
Gm-Ser1B	XM_031911923	5701200000	AB	84.29	6.258	−0.805	S (35.2)	A (20.7)	N (14.0)
Gm-P17	XM_026896665	5607100000	AB	9.31	7.906	−0.666	G (29.9)	K (16.5)	D (14.4)
Gm-P150	XM_026908157	3074000000	AB	155.41	4.745	−1.247	S (22.4)	Q (17.3)	N (7.8)
Gm-Sn1	XM_031913182	1892500000	AB	30.26	4.715	−0.535	P (15.6)	N (9.1)	F (8.0)
Gm-MG5	MG770318	1173700000	AB	65.34	10.791	−1.257	S (45.1)	N (13.3)	R (7.9)
Gm-Ser1A	MG770315	941580000	AB	94.82	3.627	−0.521	S (22.3)	Q (14.4)	A (8.6)
Gm-P22	MG770325	629610000	AB	22.24	4.497	−0.370	T (18.0)	S (14.7)	P (10.6)
Gm-ZdA	XM_026895687	478590000	AB	104.52	4.442	−0.343	C (8.7)	N (7.8)	G (7.4)
Gm-P47	XM_026903570	468680000	AB	55.37	3.894	−1.033	N (16.1)	E (13.2)	S (9.0)
Gm-GMPiso00198	XM_026896664	383160000	AB	15.44	4.006	−0.804	E (18.4)	P (17.7)	C (9.9)
Gm-MG4	XM_026893397	104810000	AB	96.02	6.345	−1.040	S (40.2)	N (24.6)	G (6.0)
Gm-Sn3	XM_026903091	100400000	AB	11.56	8.498	−0.058	N (12.3)	V (12.3)	F (8.5)
Gm-Usp03	XM_031907470	85362000	A	122.76	3.827	−0.109	T (16.8)	S (11.4)	I (8.2)
Gm-P-8	MH464805	68619000	AB	8.41	8.864	−0.381	K (12.7)	P (11.4)	A (7.6)
Gm-GMPiso00148	XM_026905986	59040000	AB	38.03	4.258	−1.217	A (19.0)	E (15.2)	K (14.9)
Gm-Usp01	XM_031910267	39692000	A	78.93	7.319	−1.186	N (13.2)	P (9.6)	S (9.5)
Gm-Usp02	XM_031911792	29142000	A	78.80	4.260	−0.445	S (14.9)	P (13.3)	Q (10.7)
Gm-Ds-like	XM_031908961	27903000	A	106.79	4.419	−0.744	S (21.0)	N (8.9)	E (8.8)
Gm-MG9	XM_031911849	25465000	AB	53.65	4.522	−0.664	S (51.4)	A (18.0)	N (12.9)
Gm-Muc-6-like	XM_026903992	12493000	A	35.23	3.737	−0.343	P (12.0)	C (10.2)	S (8.6)
Gm-Eln-like	XM_026907481	11901000	A	27.18	10.646	0.539	A (30.1)	G (14.9)	L (8.3)
Gm-Lprcp66-like	XM_026899336	11741000	A	20.19	8.846	0.022	G (24.6)	A (12.3)	Y (12.3)
Gm-P13	XM_026907679	11723000	AB	10.22	9.408	−0.535	P (14.6)	G (11.5)	A (9.4)
Gm-Papln-like	XM_031914292	8296100	A	273.14	4.388	−0.645	E (9.4)	T (9.4)	G (9.0)
Gm-Iff6-like	XM_026895003	5793800	A	49.24	4.216	−0.933	S (29.2)	G (20.2)	N (18.6)
Gm-Cp21-like	XM_026905740	5698400	A	30.92	9.426	0.609	A (35.9)	V (14.4)	P (12.5)
Gm-P-12	MH464803	5559200	AB	11.77	8.505	−0.625	G (31.4)	S (17.4)	Q (8.3)
Gm-Sn4	XM_026903191	5067300	A	11.96	7.834	−0.243	S (11.6)	G (10.7)	V (10.7)
Gm-Imuc-C.1-like	XM_031908793	2629500	A	21.24	4.321	−0.323	T (17.7)	E (8.9)	V (8.3)
Gm-Sn2	XM_031913070	2473700	AB	19.70	9.317	−0.330	A (10.9)	S (10.9)	N (10.3)
Gm-Muc5AC-like	XM_026900881	2253000	A	96.92	7.745	−0.885	T (14.5)	A (9.8)	P (9.2)
Gm-Lpcp-23-like	XM_026893171	1278700	A	35.69	9.832	0.747	A (22.8)	S (11.9)	L (9.2)
Gm-Zon-like	XM_026900349	1113800	A	46.15	7.341	−0.393	C (14.6)	P (8.9)	K (6.9)
Gm-LcpA2B-like	XM_026905750	863070	A	15.56	6.512	0.302	A (15.5)	P (15.5)	V (13.5)
Gm-Cp3-like	XM_026892615	783140	A	23.16	6.801	0.525	A (35.4)	V (9.2)	G (7.5)
Gm-Ser2-like	XM_031911858	727170	A	24.05	3.881	−0.631	S (53.5)	A (9.8)	N (7.8)
Gm-MG-2	XM_031911783	727170	AB	30.75	3.896	−0.602	S (52.3)	A (10.4)	N (6.7)
Gm-Fbn	XM_026904452	-	A	150.36	5.483	−0.756	T (11.0)	P (8.7)	C (8.0)
Gm-Pcp-like	XM_026899249	-	A	25.50	6.292	−0.592	A (26.9)	Q (12.4)	P (11.2)
Gm-Pedp1-like	XM_026894507	-	A	14.19	9.149	−0.655	P (9.5)	G (8.7)	K (7.9)
Gm-MG-1/MG-3	XM_031911850	-	B	30.62	3.823	−0.479	S (42.1)	G (15.8)	N (8.8)
Gm-MG6	XM_026893136	-	B	91.96	10.571	−1.551	S (54.6)	N (16.0)	E (6.7)
Gm-MG7	XM_031911880	-	B	55.07	3.360	−0.862	S (38.3)	N (22.4)	G (11.1)
Gm-MG8	XM_031911957	-	B	28.34	3.517	0.082	S (32.0)	G (23.2)	A (17.4)
Gm-P-11	XM_026895673	-	B	10.95	4.986	−0.291	S (12.1)	L (11.1)	G (8.1)
Gm-P-7/P14	XM_031911797	-	B	14.34	3.830	−0.261	D (14.0)	I (12.4)	S (12.4)
Gm-GMPcon00005	XM_026895223	-	B	57.59	4.701	−0.243	A (10.8)	V (9.9)	S (9.5)
Gm-GMPiso00278	XM_026899644	-	B	32.09	6.502	−0.866	P (11.0)	D (7.8)	Q (7.4)
Gm-GMPiso00090/00234	XM_026897762	-	B	26.45	8.911	−0.474	L (10.4)	S (9.5)	A (7.9)

Interestingly, we found no clear homologs of several silk proteins of *G. mellonella* in *E. kuehniella*, suggesting that these proteins may be putative species-specific genes. These include proteins such as P250 (LOC113513637), P17 (LOC113512752), MG5 (LOC113521079), P22 (LOC113513778), GMPiso00198 (LOC113512751), MG4 (LOC113509977), P-8 (LOC113513777), MG9 (LOC116413334), MG-2 (LOC113519334), P-7/P14 (LOC116413327), MG6 (LOC113509728), MG-1/MG-3 (LOC113517751), MG7 (LOC113522155), MG8 (LOC116413345), P-11 (LOC113511945), GMPiso00090/GMPiso00234 (LOC113513780) were previously characterized. Several other novel proteins were detected in this study in the silk of *G. mellonella* and automatically annotated as dentin sialophosphoprotein-like (LOC113509273), cuticle protein LPCP-23-like (LOC113509759), cell wall protein IFF6-like (LOC113511377), serine-rich adhesin for platelets-like (LOC113523571), protein PB18E9.04c-like (LOC113512274), and sericin-2-like (LOC113522365; see Table 2).

This comparative approach allowed us to overcome some limitations of the proteomic analysis and helped to identify additional silk genes. The final list of *E. kuehniella* and *G. mellonella* candidate silk proteins is shown in Tables 1, 2.

### Some of the silk genes are arranged in clusters

**FIGURE 5:**
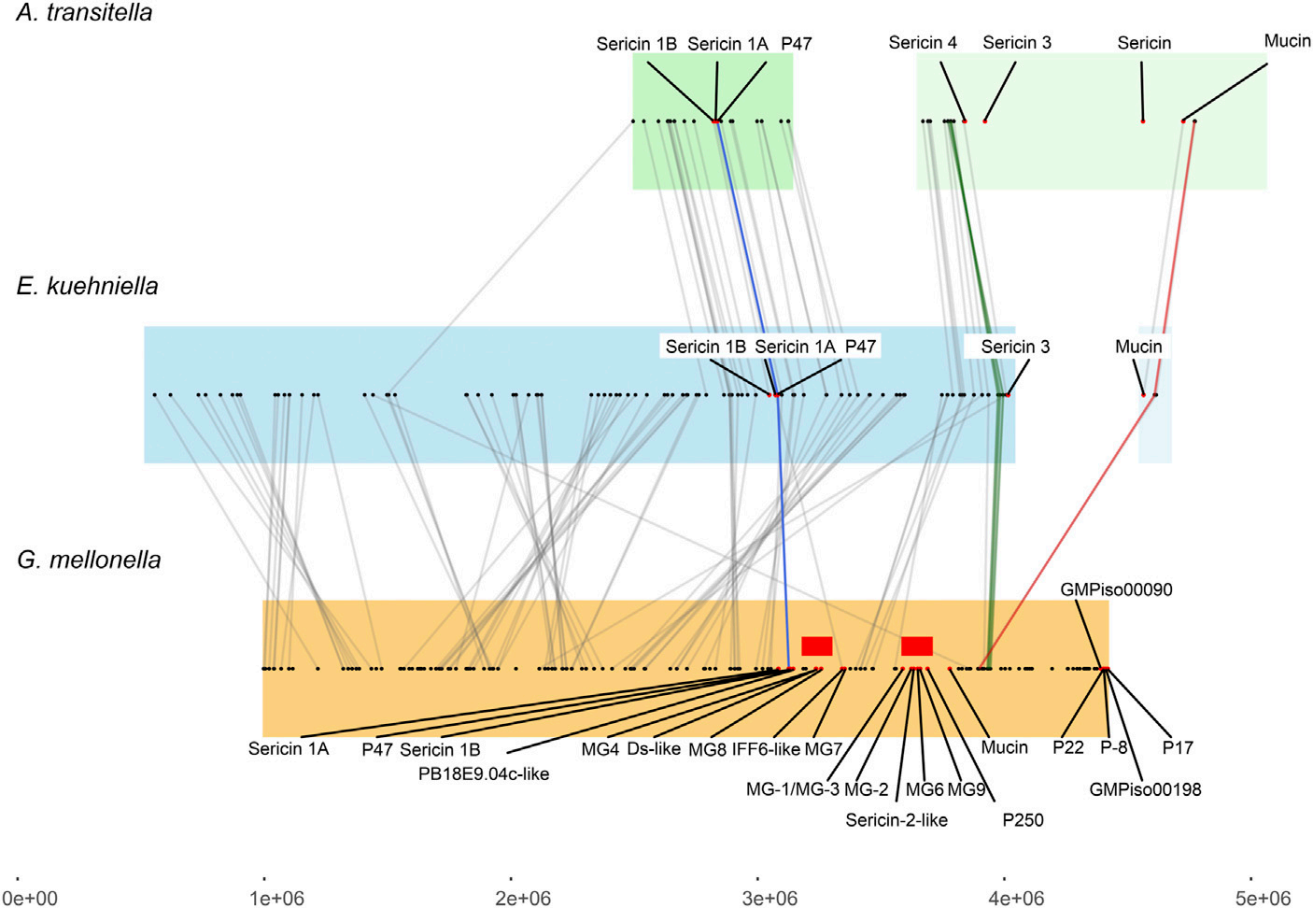
Collinearity analysis of sericin genes among *E. kuehniella*, *G. mellonella*, and *A. transitella*. Collinear blocks are highlighted with background colors. Silk genes are indicated with red dots. Red rectangles indicate the area of the *G. mellonella* genome containing recently duplicated silk genes. The color and gray lines connect the syntenic gene pairs. The proposed “landmark” genes (see Supplementary Table S5 for gene names) that are adjacent to the silk genes in three species are connected with blue, green and magenta lines.

Our results show that several silk genes including sericins, seroins, and zonadhesins, form clusters on different chromosomes. The genes for sericins were located in two clusters that are conserved between species to some extent. However, the microsynteny of one of these clusters appears to be impaired in *G. mellonella* compared to *E. kuehniella,* and *A. transitella*. Figure 5 shows the microsynteny of a genomic region containing mainly genes for sericins from three moths flanked by evolutionarily conserved genes (see Table 3). The lines connect the putative orthologs of the conserved genes. Comparison of sericin coding regions between species showed that a number of sericin genes are in tight cluster, which is present only in *G. mellonella* but not in the other two species. Our data support the hypothesis that the genomic region encoding the sericin gene cluster has been recently duplicated in *G. mellonella*.

**TABLE 3:** Comparison of protein parameters of FibH including number of amino acid residues, molecular weight, percentage of three major amino acids, H.I. - hydropathy index (GRAVY) and isoelectric pont (pI). Genbank accession numbers: *G. mellonella* (XM_026905081), *E. kuehniella* (ON604816), *P. interpunctella* (JAJAFS010000023.1), *A. suavella* (OW971947.1), *E. flammealis* (LR990872.1), *H. costalis* (OW443343.1), *C. exigua* (CM032477.1), *Ch. Suppressalis* (OU963910.1), *A. ephemerella* (OW971889.1), *B. mori* (NM_001113262.1), *A. yamamai* (AB542805.1) and *S. cynthia* (AB971865).

Species	Family	Subfamily	Number a.a.	Mw	Ala (%)	Gly (%)	Ser (%)	H. I.	pI
*Galleria mellonella*	Pyralidae	Galleriinae	6020	487830	23.30	31.30	17.60	0.553	3.94
*Ephestia kuehniella*	Pyralidae	Phycitinae	5631	493710.7	19.10	21.90	23.10	0.054	3.81
*Plodia interpunctella*	Pyralidae	Phycitinae	4714	413334.4	26.30	18.30	18.50	0.084	4.04
*Acrobasis suavella*	Pyralidae	Phycitinae	6057	534495.5	15.50	23.9	24.20	-0.438	3.78
*Endotricha flammealis*	Pyralidae	Pyralinae	8027	685384.5	34.90	20.00	19.90	0.164	3.43
*Hypsopygia costalis*	Pyralidae	Pyralinae	5981	521977.6	22.20	24.9	25.70	-0.17	3.323
*Cnaphalocrocis exigua*	Crambidae	Pyraustinae	5418	485846.3	20.60	26.30	5.10	-0.452	4.31
*Chilo suppressalis*	Crambidae	Crambinae	4386	383147.9	43.10	16.00	13.00	0.011	5.33
*Acentria ephemerella*	Crambidae	Nymphulinae	6151	533043.56	21.9	27.8	10.3	-0.138	9.78
*Bombyx mori*	Bombycidae	Bombycinae	5263	391633.58	30.2	45.9	12.1	0.216	4.39
*Antheraea yamamai*	Saturniidae	Saturniinae	2856	234576.05	42.9	27.2	11.0	0.186	4.49
*Samia cynthia*	Saturniidae	Saturniinae	2880	227361.93	45.4	31.7	6.7	0.336	4.85

### Phylogenetic relationships among silk genes

**FIGURE 6:**
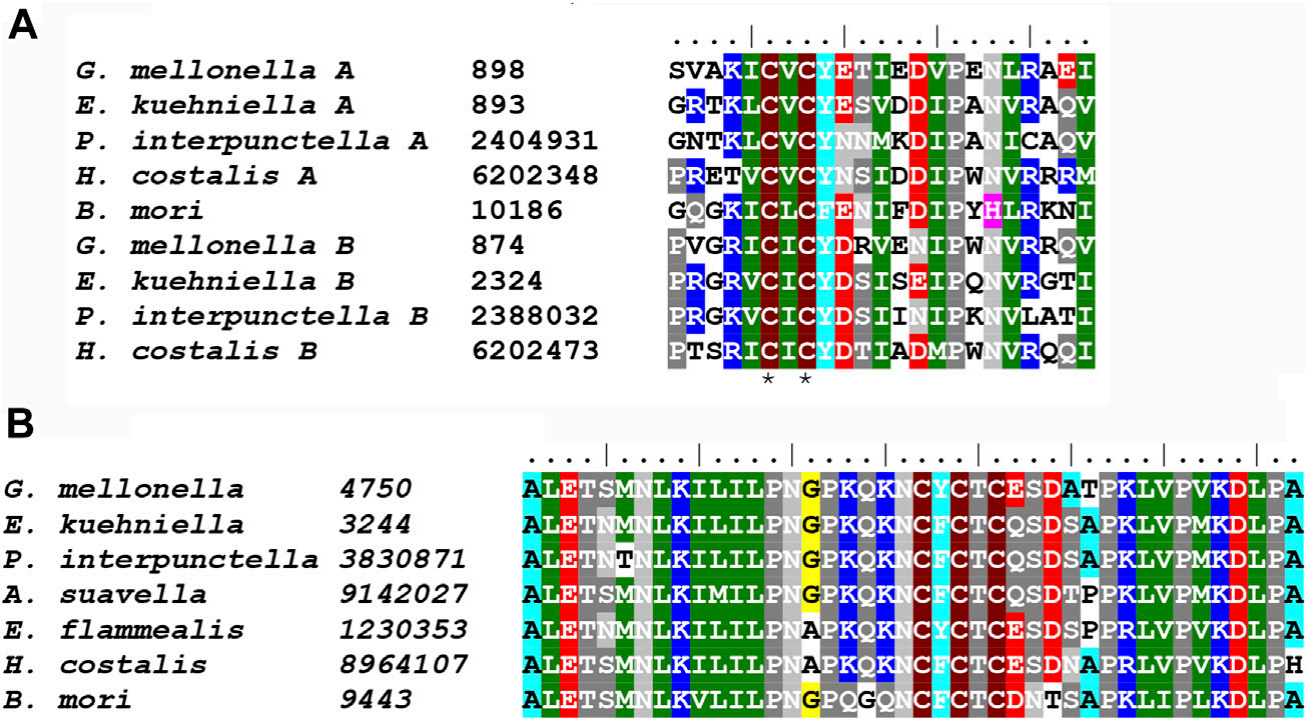
**(A)** Multiple sequence alignment of Ser1 C-terminal sequences containing conserved CXCX motif: *G. mellonella* Ser1A (MG770315), *E. kuehniella* Ser1A (ON604817) *P. interpunctella* Ser1A (JAJAFS010000011.1), *H. costalis* Ser1A (OW443355.1), *B. mori* Ser1 (XM_038013610.1), *G. mellonella* Ser1B (XM_031911923), *E. kuehniella* Ser1B (ON604818), *P. interpunctella* Ser1B (JAJAFS010000011.1), *H. costalis* Ser1B (OW443355.1). Conserved cysteine residues are marked with asterisks. **(B)** Multiple sequence alignment of Muc1 C-terminal sequences containing conserved CXCXCX motif: *G. mellonella* (MG770312.1), *E. kuehniella* (ON604821), *P. interpunctella* (JAJAFS010000011.1), *A. suavella* (OW971956.1), *E. flammealis* (LR990879.1), *H. costalis* (OW443355.1), *B. mori* (XM_021350004.2). Conserved cysteine residues are marked with asterisks.

The two major classes of putative adhesive proteins produced by the MSG are sericins and mucins. They are generally encoded by large genes with repetitive sequences that contain a high proportion of serine residues. We identified at least four putative sericin proteins (Ek-Ser1A, Ek-Ser1B, Ek-Ser3 and Ek-Ser4), three different mucins (Ek-Muc1, Ek-Muc2 and Ek-Muc3), and one protein (Ek-P150) in *E. kuehniella* that can be classified as both a sericin and a mucin. Two sericin-1-like proteins, Ek-Ser1A and Ek-Ser1B, carry a CXCX motif near the C-terminus, whereas Ek-Muc1 has the three-Cys motif NCFCTC near the C-terminus (Figure 6), similar to the KCYCSC motif of Ek-P150. Such motifs seem to be conserved among species.

It has previously been suggested that sericin 1 (Ser1), mucin-1 (Muc1), and P150 loci might be related (Kludkiewicz et al., 2019). To explore possible phylogenetic relationships, we tested these sequences in one dataset. The phylogram (Supplementary Figure S1A) clearly shows, apart from mucin-1, a distinct group of Ser1 and P150. Within this group, the P150 loci formed well-supported subclusters, but the discrimination of Ser1 and P150 may not be complete because Gm-Ser1A is separated from the other Ser1-like proteins.

We found four seroin-like proteins localized in a single cluster in the genomes of both *E. kuehniella* and *G. mellonella*. All four seroin types contained putative signal peptides. Except for Ek-Sn2, all were found in the cocoons of both species *via* proteomic analysis (Ek-Sn2 was inferred from homology and its expression was validated by qPCR). Previously, only three seroin genes were identified in *G. mellonella* (Kludkiewicz et al., 2019). As shown in Supplementary Figure S1B, the newly discovered seroin 4 forms a separate branch.

Within the Pyraloidea superfamily, species have only one copy of the *fibrohexamerin (P25)* gene, and its genealogy follows the phylogeny of the Pyraloidea (Supplementary Figure S1C). At least four zonadhesin-like proteins have been detected in *E. kuehniella*. Zonadhesins apparently have EGF_2/TIL domains (these domains partially overlap; see Supplementary Table 4). Phylogenetic analysis revealed that these genes belong to three well-delineated clusters (Supplementary Figure S1D). A schematic diagram showing the evolutionary relationships of the Pyraloidea moths is also shown for comparison in Supplementary Figure 1E (adapted from Regier et al., 2012).

### Comparison of FibH proteins from nine species of pyraloidea

To learn more about the specific and conserved features of FibH proteins, we identified seven additional *fibH* genes from pyralid moths in assemblies published by the Wellcome Sanger Institute. The species comprise two families (five members of Pyralidae and three Crambidae) and six subfamilies. The list of species and protein parameters is shown in Table 3. A schematic representation of the FibH sequences is shown in Supplementary Figure S2.

**FIGURE 7:**
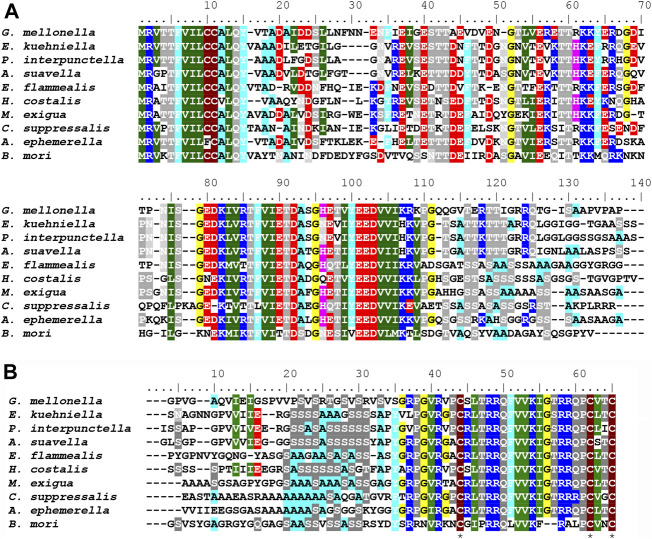
**(A)** Multiple sequence alignment of FibH N-terminal sequences **(B)** Multiple sequence alignment of FibH C-terminal sequences Conserved cysteine residues are marked with asterisks. Genbank accession numbers are listed in Table 3. Complete amino acid FibH sequences are shown in Supplementary Figure S2.

The length of fibroin proteins ranges from 4386 (*Ch. suppressalis*) to 8027 amino acids (*E. flammealis*). As expected, FibH molecules consist of nonrepetitive N- and C-termini that are well conserved, and large repetitive regions, that are highly species-specific (Figure 7). As shown in Supplementary Figure S2, the arrangements and lengths of the repetitive sequences vary considerably, but all sequences of the Pyralidae memebrs (*A. suavella*, *E. flammealis*, *H. costalis*, *G. mellonella* and *P. interpunctella*) have clusters of amino acid residues similar to *E. kuehniella* VIVIEENQSSAAAAASSSSS with 4 hydrophobic amino acids and 1-4 hydrophilic amino acids, and a crystalline domain with a block of alanine and serine residues. The hydrophobic motif also occurs in *A. ephemerella* which belongs to the family Crambidae and in the C-terminal part of *C. exigua* FibH. while in *Ch. suppressalis* this motif does not exist (Supplementary Figure S2).

There are major differences in the hydrophobicity of the FibH proteins, with *G. mellonella* having the most hydrophobic fibroin, whereas the silks of *A. suavella* and *C. exigua* are very hydrophilic (Table 3). The FibH of *E. kuehniella* is much less hydrophobic than the FibH of *G. mellonella* (see Supplementary Figures S2A,B), which is probably related to the high hygroscopicity and solubility of this silk (see above). The fibroin genes can also be divided into two categories according to the regularity of the arrangement of the repeated sequences, with the fibroins of *E. kuehniella* and *G. mellonella* showing a very regular arrangement of these sequences while *Ch. suppressalis* is the most irregular (Supplementary Figure S2I). Interestingly, *A. ephemerella* FibH contains 3.7% tyrosine residues and the isoelectric point (pI) is 9.62 which is reminiscent of the FibH from caddisfly *P. conspersa* (Rouhová et al., 2022). *A. ephemerella* is an aquatic insect whose larva pupates in an underwater cocoon filled with air.

## Discussion

In this study, we identified 30 genes encoding major silk components of *E. kuehniella* cocoon and verified specificity of their expression. We also analyzed silk genes from *G. mellonella*, which belongs to the same moth family. By comparing the silk genes of the two species, we gained insight into the degree of divergence between the species and found that several orthologs of genes encoding sericins present in *G. mellonella* are absent in *E. kuehniella*. In addition, we annotated the *fibH* genes of several other members of the Pyraloidea and analyzed their sequences.

### Specific features of silk from pyralid moths

The silks studied so far are characterized by the insolubility of the fibers and the solubility of the sericin coating. Consequently, the fibroins of *B. mori*, *A. yamamai*, and other saturniids are hydrophobic with an GRAVY index representing the hydrophobicity (Kyte and Doolittle, 1982) ranging from 0.186 to 0.336. Interestingly, the GRAVY indexes of fibroins in the Pyraloidea vary greatly from the extremely hydrophobic fibroin of *G. mellonella* (GRAVY = 0.553) to the hydrophilic fibroins of *A. suavella* or *C. exigua* with a negative GRAVY index of −0.440 and −0.452, respectively. The silk of *E. kuehniella* exhibits intermediate hydrophobicity (GRAVY = 0.054), and is readily soluble under the conditions used in degumming the silk of other species.

Comparison of available FibH sequences from Pyraloidea revealed remarkable differences in size, amino acid composition, structure of repeats etc. These molecules contain putative crystalline regions consisting of Ala and Ser residues typical of molecules of the X-ray class III (Lucas and Rudall, 1968), which are shorter than similar S(A)_13-15_S motifs in FibH proteins of *A. yamamai* or *S. ricini*. Crystalline sequences include the SSAAAAASSSS motif in *E. kuehniella* and the SSAASAAAA motif in *G. mellonella* (Supplementary Figure S2). Previous experiments have shown that fibers with regularly ordered repeat sequences of fibroins from *G. mellonella* and *E. kuehniella* have much higher tensile strength than fibers from *P. interpunctella* with disordered repeat sequences (Fedic et al., 2003). Interestingly, *C. exigua*, contains a crystalline A_8_S_2_ sequence accompanied by (PXX)_8–21_ motifs (Supplementary Figure S2). Such motifs have been shown to form so called polyproline II helices that can self-assemble and form compacted structures (Jin et al., 2009).

It has been consistently reported that the silks of some arthropod species can absorb considerable amounts of water and that they are quite hygroscopic; for example, the aggregate glue in spider webs absorbing atmospheric water and dissolving glycoproteins so that they spread and adhere upon contact with flying insects (Opell and Stellwagen, 2019). The silk of *B. mori* can absorb up to 30% of its weight in water (Hasan et al., 2019). Silk is considered as a highly hygroscopic material, and degummed silk is slightly less hygroscopic because the sericins absorb better than the fibre (Sonwalkar, 1993). Our results show that the hygroscopicity of *E. kuehniella* silk is extremely high, at least twice that of *B. mori* or *G. mellonella*. It is likely that both the sericins and fibroin core contribute to this property. The high hygroscopicity of *E. kuehniella* silk is possibly an adaptation to the dry environment in which Mediterranean flour moths and other members of subfamily Phycitinae live. The cocoon of *E. kuehniella* may help it absorb water from the air and protect the pupa from desiccation. It has previously been reported that *E. kuehniella* silk appears to increase moisture in stored agricultural products, increasing the likelihood of fungal outbreaks (https://www.internationalpheromones.com/product/meal-moths-ephestia-plodia-species/).

### Genes encoding coating proteins

The adhesive proteins that form the envelope around the fibroin core can be formally divided into several classes, including Ser1-like proteins, high-serine outer layer sericins, P150-like sericins, mucins, and zonadhesin-like proteins.

Ser1-like proteins are expressed in the rear part of MSG and are deposited on the fibroin core as the first sericin layer (Takasu et al., 2007; Kludkiewicz et al., 2019; Rouhova et al., 2021). *B. mori* contains a single Ser1-like gene consisting mainly of a repetitive sequence of 38 amino acid residues, of which 31% are serine residues. It is expressed in the middle and posterior regions of the MSG, and four to five Ser1 transcripts are generated by alternative splicing. The truncated Ser1 mutant of *B. mori* tends not to spin and often forms coarse cocoons (Takasu et al., 2017). Interestingly, Ser1-like proteins in other species, such as *E. kuehniella* and *G. mellonella*, are encoded by two genes (Ser1A and Ser1B), encoding proteins with 14–17% serine residues in *E. kuehniella* and 22–35% serine residues in *G. mellonella*. All Ser1 proteins contain CXCX motifs near their C-terminus, including those of *B. mori*, *A. yamamai*, and *T. bisselliella* (Kludkiewicz et al., 2019). Ser1-like proteins may represent a constant sericin component present in most moth silks and could have a specific function by directly covering the fibroin fiber as the innermost layer.

It has been reported that *B. mori* has only one sericin protein in the cocoon besides Ser1, namely sericin 3 (sericins 2 and 4 are not present in the cocoon silk) (Takasu et al., 2007; Dong et al., 2019). Ser3 is characterized by a high serine content, is localized in the outer silk layer, and possibly serves as a lubricant to reduce friction during secretion (Takasu et al., 2007). We discovered a putative sericin gene product from *E. kuehniella* (Ek-Ser4), which contains more than 40% serine residues, resembling Ser3 of *B. mori* or MG2 or MG6 of *G. mellonella*. In contrast, there are at least eight sericins with high-serine in *G. mellonella.* Homologs of these *G. mellonella* genes are most likely absent in *E. kuehniella*. Previous phylogenetic analyses support the idea that sericins resembling Ser3 in *B. mori* expand multiple times during evolution, as suggested by the species-specific branching of sericin proteins in the phylogenetic trees of *G. mellonella, A. yamamai* and *S. cynthia ricini* (Kludkiewicz et al., 2019). The high proportion of sericins in the silk of *G. mellonella* may contribute to the compactness of the cocoon in this species as required for its protection from bees. Our results suggest that sericins may serve as adhesives, lubricants or regulators of silk compactness and cross-linking of silk proteins.

Previous phylogenetic analyses have shown that the cocoon of *G. mellonella* contains a very abundant sericin-like protein called P150. It appeared to be phylogenetically quite distant from other sericin genes (Kludkiewicz et al., 2019). In this study, we discovered the putative homolog of this protein in *E. kuehniella* and named it Ek-P150. Phylogenetic analyses show that P150-like proteins form a distinct group that can be classified as both mucin and sericin because they contain serine rich repeats and a CXCXCX motif near their C-termini.

Mucins form a distinct group of silk proteins that contain serine-rich repetitive sequences. Unlike sericins they include repeats with threonine and proline residues (Syed et al., 2008). Such proline-threonine-serine motifs usually account for about one-third of the total length of the mucin protein (Perez-Vilar and Hill, 1999). We found at least three mucins in the silk of *E. kuehniella* and at least four in the silk of *G. mellonella*. At least one of them in each species could represent mucin-1-like proteins with a CXCXCX motif near the C-terminus. Our study is consistent with the idea of a common origin of the mucin and sericin families.

### Impact of omics methods on silk research

Omics methods allow silk to be studied with great breadth and resolution by capturing large, if not complete, populations of genes or proteins that are related to their structure and elucidating the evolutionary and structural relationships among them. In addition, parallel study of related species allows comparison and completion of missing information. Comparative data can be also supplemented using sequences from high-quality genomes published by Wellcome Sanger Institute and other sources. However, due to the rapid diversification of these proteins, the similarities among silk proteins are not obvious, sequence conservation is rare and limited to species of the same family or subfamily. Identification of the structural components of silk still requires “traditional methods”. The lack of similarity and correct annotation of genomic data is an important limitation. Here we were able to detect heavy chain fibroins in a number of pyralid moths based on sequence conservation in both ends of the fibroin molecules and compare their structures. Our results suggest that Ser1 and Muc1 can also be detected based on similarity. The use of BLAST methods for most other genes is still limited and needs more information.

Using omics data, we analyzed homologous regions on chromosomes and traced the synteny of putative sericin sequences in tight clusters. Detailed analysis revealed that the sericin region containing at least 12 genes in the *G. mellonella* genome is likely the result of a recent duplication. Synteny provides a robust framework for the identification of homologs to known genes, helps searching for new genes, and provides important information on annotation. For example, two of the genes localized in the *G. mellonella* genomic sericin cluster—annotated as dentin sialophosphoprotein-like (XM_031908961) and cell wall protein IFF6-like (XM_026895003)—are expressed in SGs, contain a signal peptide, a repetitive sequence, and a high proportion of serine residues (21% and 29%), and thus appear to belong to the sericin family of *G. mellonella*.

Overall, we identified the silk genes in *E. kuehniella* and *G. mellonella* based on proteomic analysis of cocoon silk and by homology searches in the transcriptomes and genomes of both species. We discovered a region containing clusters of sericin genes and identified blocks of synteny between both genomes (colocalized gene clusters shared between genomes). The resulting microsynteny map allowed identification of duplication events in the sericin family. Finally, we present the complete primary structures of nine *fibH* genes and proteins from both families of the suborder Pyraloidea and discuss their specific and conserved features.

## Data Availability

The trancriptome assembly was deposited in the Dryad repository (https://doi.org/10.5281/zenodo.7273794). The experimental data that support the findings of this study is available within this article or its Supplementary Material. List of silk gene candidates their GenBank accession codes are listed in Tables 1, 2. The sequences of FibH from 9 pyralid species are available as Supplementary Data (Supplementary Figure S2).
