# Supplementary material for: Characterization of silk genes in Ephestia kuehniella and Galleria mellonella revealed duplication of sericin genes and highly divergent sequences encoding fibroin heavy chains
Source: Front Mol Biosci. 2022 Nov 29;9:1023381. doi: 10.3389/fmolb.2022.1023381 (PMC9745057; doi:10.3389/fmolb.2022.1023381)
Supplement: Supplementary file 1 [file DataSheet3.pdf]

Supplementary Material

Supplementary Figure 1. Phylogenetic analysis of selected silk proteins in Pyraloidea species and evolutionary relationships among Pyraloidea.

Supplementary Figure 2. Supplementary Figure 2. FibH protein sequences of nine pyraloid moths predicted from the genomic regions using online Augustus software (Stanke and Morgenstern, 2005).

Supplementary Table 1. Summary of primer sequences used in *E. kuehniella* for (A) northern blot probes and (B) real-time qPCR.

Supplementary Table 2. Statistics of expression levels of selected silk genes detected by qPCR in *E. kuehniella*. AMS, anterior-middle SG; MMS, middle-middle SG; RMS, rear-middle SG; PS, posterior SG; PS, anterior-middle SG; WLWS, wandering larva without SG. Statistical differences (T-test; $P < 0.05$) are indicated by asterisk (*).

Supplementary Table 3. (A) BUSCO assessment of initial and improved transcriptome of *E. kuehniella*. (B) Genome assembly statistics for *E. kuehniella*.

Supplementary Table 4. Summary of domains identified in zonadhesin protein sequences of *E. kuehniella*, *G. mellonella*, *A. transitella*, and *Danaus plexippus plexippus*. The search was performed using the web tool MOTIF Search (https://www.genome.jp/tools/motif/) against motif library PROSITE Pattern, PROSITE Profile and Pfam.

Supplementary Table 5. Summary of proposed landmark genes linked with silk genes in *E. kuehniella*, *G. mellonella* and *A. transitella*. These genes are evolutionarily conserved and their positions in the genomes of the three species are shown in Figure 5. The corresponding genes in Figure 5 are connected by coloured lines (blue, green and magenta).

Supplementary Figure 1. Phylogenetic analysis of selected silk proteins in Pyraloidea species and evolutionary relationships among Pyraloidea. (**A-D**) Phylograms of selected silk proteins. The evolutionary history was inferred by the Maximum Likelihood method with 1000× bootstrap. The percentage of trees in which the associated taxa clustered together is shown below the branches. The tree is drawn to scale, with branch lengths measured in the number of substitutions per site. Abbreviations of species are as follows: Amtr, *A. transitella*; Epku, *E. kuehniella*; Game, *G. mellonella*; Stti, *Stenopsyche tienmushanensis*; Epel, *Ephestia elutella*; Coce, *Corcyra cephalonica*; Hade, *Haritalodes derogata*; Osfu, *Ostrinia furnicalis*; Dapl, *Danaus plexippus*; Pcon, *Plectrocnemia conspersa*. (A) Sericins / mucins / P150. Based on TIM+F+I transition model. Amtr – Ser1A (LOC106134399), Ser1B (LOC106134400 transcript X1), Muc (LOC106133704), P150 (LOC106132366); Epku – Ser1A (ON604817), Ser1B (ON604818), Muc (ON604821), P150 (ON604820); Game – Ser1A (LOC113519119, transcript X1), Ser1B (LOC113512273), Muc (MG770312), P150 (LOC113522468). (B) Seroins. Based on HKY+F+G4 model. Amtr – Sro1 (LOC106133523), Sro2 (LOC106133521), Sro3 (LOC106133479), Sro4 (LOC106133522), Sro-like (LOC106133524); Epku – Sro1 (ON604827), Sro2 (OP185488), Sro3 (OP185489), Sro4 (ON604828); Game – Sro1 (LOC113518338), Sro2 (LOC113518101), Sro3 (LOC113518258), Sro4 (LOC113518326); Stti – Sro (Steno.02678-RA). (C) P25. Based on TN+F+I+G4 model. Epku – Fhx (ON604823); Epel – Fhx part (00014683-RA); Amtr – Fhx-like (LOC106131755); Game – Fhx (LOC113510933); Coce – P25 (GQ901976); Hade – P25 (KY792994); Osfu – Fhx-like (LOC114362712); Dapl – Fhx-like (LOC116776386). (D) Zonadhesins. Based on GTR+F+I+G4 model. Amtr – zon-like1 (LOC106138817), zon-like2 (LOC106136372), zon-like3 (LOC106130156), zon-like4 (LOC106131409); Epku – Zon01 (ON604824), Zon02 (ON604825), Zon03 (ON604826), zon-like (OP185494); Game – zon-like1 C2 (LOC113516017), zon-like2 C3 (LOC113511957), zon-like3 C1 (LOC113519003), zon-like4 (LOC113511955), ZdB C3 (MG770321), ZdA C3 (MG770320), uncharacterized protein (LOC113509084), zon_C2 (LOC113511802); Dapl – zon-like C3 (LOC116765598), zon C2 (LOC116771299); Pcon – Zon8 (OL405648). (**E**) The evolutionary relationships of the Pyraloidea (adapted from Regier et al., 2012, DOI: 10.1111/j.1365-3113.2012.00641.x.).

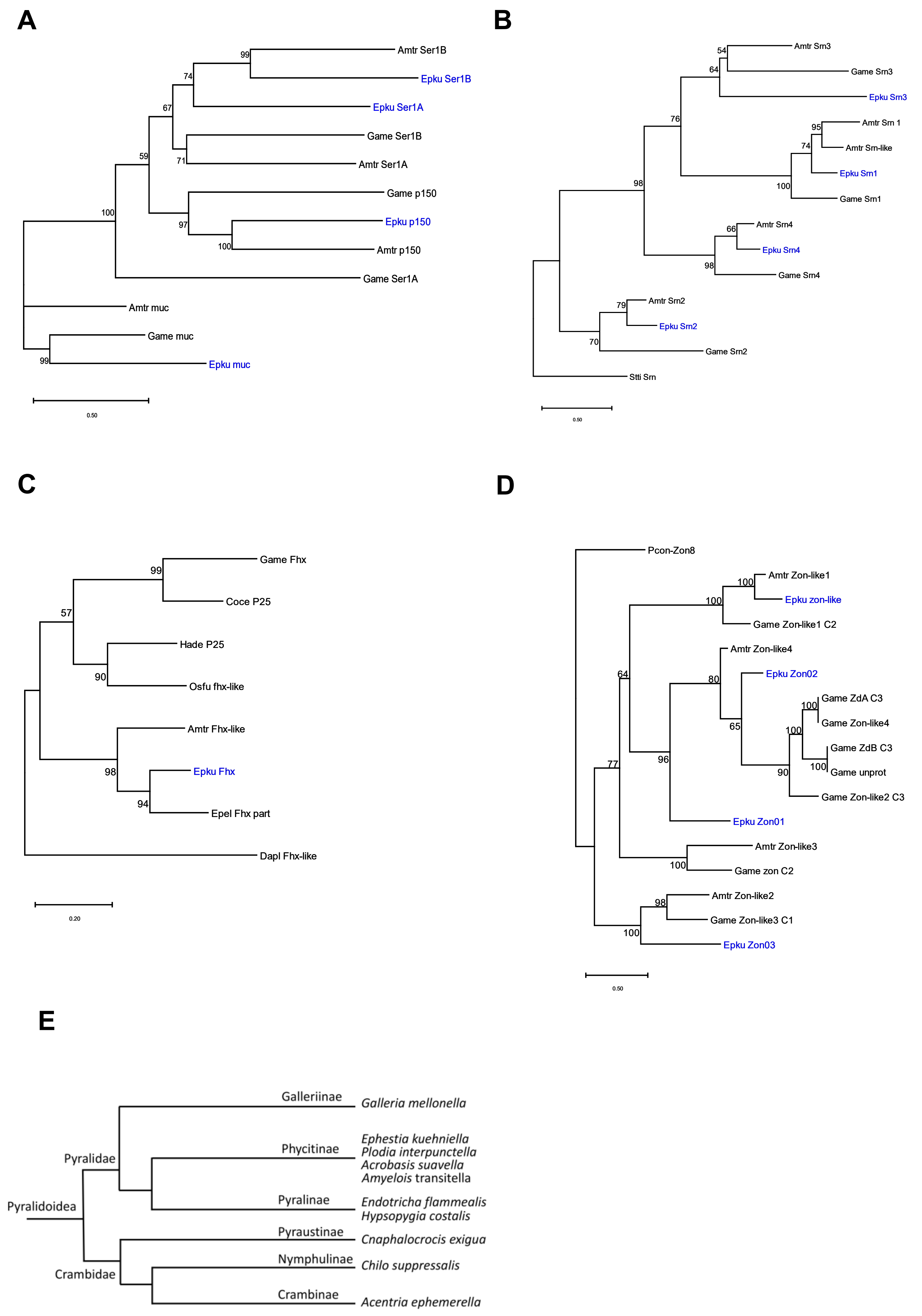

A
Amtr Ser1B
Epku Ser1B
Epku Ser1A
Game Ser1B
Amtr Ser1A
Game p150
Epku p150
Amtr p150
Game Ser1A
Amtr muc
Game muc
Epku muc
0.50
B
Amtr Srn3
Game Srn3
Epku Srn3
Amtr Srn 1
Amtr Srn-like
Epku Srn1
Game Srn1
Amtr Srn4
Epku Srn4
Game Srn4
Amtr Srn2
Epku Srn2
Game Srn2
Stti Srn
0.50
C
Game Fhx
Coce P25
Hade P25
Osfu fhx-like
Amtr Fhx-like
Epku Fhx
Epel Fhx part
Dapl Fhx-like
0.20
D
Pcon-Zon8
Amtr Zon-like1
Epku zon-like
Game Zon-like1 C2
Amtr Zon-like4
Epku Zon02
Game ZdA C3
Game Zon-like4
Game ZdB C3
Game unprot
Game Zon-like2 C3
Epku Zon01
Amtr Zon-like3
Game zon C2
Amtr Zon-like2
Game Zon-like3 C1
Epku Zon03
0.50
E
Galleriinae
Galleria mellonella
Phycitinae
Ephestia kuehniella
Plodia interpunctella
Acrobasis suavella
Amyelois transitella
Pyralidae
Pyralidoidea
Pyralinae
Endotricha flammealis
Hypsopygia costalis
Pyraustinae
Cnaphalocrocis exigua
Nymphulinae
Chilo suppressalis
Crambidae
Crambinae
Acentria ephemerella

Supplementary Figure 2. FibH protein sequences of nine pyraloid moths predicted from the genomic regions using online Augustus software (Stanke and Morgenstern, 2005). Amino acids are highlighted with cyan (hydrophilic: RKDENQ), magenta (hydrophobic: YVMCLFIW), or no shadings (netural: SGHTAP). Genbank accession codes: *E. kuehniella* (ON604816), *G. mellonella* (NHTH01000008.1/NHTH01000133.1), *E. flammealis* (LR990872.1), *H. costalis* (OW443343.2), *P. interpunctella* (JAJAFS010000023.1), *A. suavella* (OW971947.1), *A. ephemerella* (OW971889.1), *C. exigua*, (CM032477.1), *Ch. suppressalis* (OU963910.1).

(A) ***Ephestia kuehniella*** **FibH**

MRVTTFVILCCALQYVAAADILETGILGGNVREVSESTTDNFTTDGNGNVTEVKTTHKEFRRQGEVPNNISGEDKLVRTFVIETDASGNEVIYEEDVVIHKVPGTSATTKTTTARRQLGGIGGTGAASSSAATSPPRPTSPYGPNGSSSAATSSASGSAP
VIVIEENQSSAAAAAASSSSSGPNGLLGPYGPNGSAASSAAASGAAGAGGLNGLYGSNGALLSGAAGVNGLNGLYGPNGSSASSAGAAGIGGLNGLNGSNGSASAASSSASGSGP
VIVIEENQSSAAAAAASSSSSGQNGLLGPYGPNGSSASSAASSGAAGAGGLNGIYGSNGALLSGAAGANGLNGLYGPNGSSASSAGAAGIGGLNGLNGSNGSASAASSSASGSGP
VIVIEENQSSAAAAAASSSSSGQNGLLGPYGPNGSAASSAAASGAAGAGGLNGLYGSNGGLLSGAAGVNGLNGLYGPNGSSASSAGAAGIGGLNGLNGSNGSASAASSSASGSGP
VIVIEENQSSAAAAAASSSSSGQNGLLGPYGPNGSAASSAAASGAAGAGGLNGLYGSNGALLSGAAGVNGLNGLYGPNGSSASSAGAAGIGGLNGLNGSNGSASAASSSASGSGP
VIVIEENQSSAAAAAASSSSSGQNGLLGPYGPNGSAASSAAASGAAGAGGLNGLYGSNGALLSGAAGVNGLNGLYGPNGSSASSAGAAGIGGLNGLNGSNGSASAASSSASGSGP
VIVIEENQSSAAAAAASSSSSGQNGLLGPYGPNGSAASSAAASGAAGAGGLNGLYGSNGALLSGAAGVNGLNGLYGPNGSSASSAGAAGIGGLNGLNGSNGSASAASSSASGSGP
VIVIEENQSSAAAAAASSSSSGQNGLLGPYGPNGSAASSAAASGAAGAGGLNGLYGSNGALLSGAAGVNGLNGLYGPNGSSASSAGAAGIGGLNGLNGSNGSASAASSSASGSGP
VIVIEENQSSAAAAAASSSSSGQNGLLGPYGPNGSSASSAASSGAAGAGGLNGIYGSNGALLSGAAGANGVNGLYGPNGSSASSAGAAGIGGLNGLNGSNGSASAASSSASGSGP
VIVIEENQSSAAAAAASSSSSGQNGLLGPYGPNGSAASSAAASGAAGAGGLNGLYGSNGALLSGAAGVNGLNGLYGPNGSSASSAGAAGIGGLNGLNGSNGSASAASSSASGSGP
VIVIEENQSSAAAAAASSSSSGQNGLLGPYGPNGSAASSAAASGAAGAGGLNGLYGSNGALLSGAAGVNGLNGLYGPNGSSASSAGAAGIGGLNGLNGSNGSASAASSSASGSGP
VIVIEENQSSAAAAAASSSSSGQNGLLGPYGPNGSAASSAAASGAAGAGGLNGLYGSNGALLSGAAGVNGLNGLYGPNGSSASSAGAAGIGGLNGLNGSNGSASAASSSASGSGP
VIVIEENQSSAAAAAASSSSSGQNGLLGPYGPNGSAASSAAASGAAGAGGLNGLYGSNGALLSGAAGANGLNGLYGPNGSSASSAGAAGIGGLNGLNGSNGSASAASSSASGSGP
VIVIEENQSSAAAAAASSSSSGQNGLLGPYGPNGSAASSAAASGAAGAGGLNGLYGSNGALLSGAAGVNGLNGLYGPNGSSASSAGAAGIGGLNGLNGSNGSASAASSSASGSGP
VIVIEENQSSAAAAAASSSSSGQNGLLGPYGPNGSAASSAASSGAAGAGGLNGIYGSNGALLSGAAGANGVNGLYGPNGSSASSAGAAGIGGLNGLNGSNGSASAASSSASGSGP
VIVIEENQSSAAAAAASSSSSGQNGLLGPYGPNGSAASSAAASGAAGAGGLNGLYGSNGALLSGAAGVNGLNGLYGPNGSSASSAGAAGIGGLNGLNGSNGSASAASSSASGSGP
VIVIEENQSSAAAAAASSSSSGQNGLLGPYGPNGSAASSAAASGAAGAGGLNGLYGSNGALLSGAAGVNGLNGLYGPNGSSASSAGAAGIGGLNGLNGSNGSSAAASSSASGSGP
VIVIEENQSSAAAAAASSSSSGPNGLLGPYGPNGSSASSAGAAGIGGLNGLNGSNGSASAASSSASGSGP
VIVIEENQSSAAAAAASSSSSGPNGLLGPYGPNGSSASSAASSGAAGAGGLNGIYGSNGALLSGAAGVNGLNGLYGPNGSSASSAGAAGIGGLNGLNGSNGSASAASSSASGSGP
VIVIEENQSSAAAAAASSSSSGQNGLLGPYGPNGSAASSAAASGAAGAGGLNGLYGSNGALLSGAAGVNGLNGLYGPNGSSASSAGAAGIGGLNGLNGSNGSASAASSSASGSGP
VIVIEENQSSAAAAAASSSSSGQNGLLGPYGPNGSAASSAAASGAAGAGGLNGIYGSNGALLSGAAGVNGLNGLYGPNGSSASSAGAAGIGGLNGLNGSNGSASAASSSASGSGP
VIVIEENQSSAAAAAASSSSSGPNGLLGPYGPNGSSASSAGAAGIGGLNGLNGSNGSSSAAGIGGLNGLNGSNGSSSAASSSASGNGP
VIVIEENQSSAAAAAASSSSSGPNGLLGPYGPNGSSASSAGAAGIGGLNGLNGSNGSASAASSSASGSGP
VIVIEENQSSAAAAAASSSSSGQNGLLGPYGPNGSAASSAAASGAAGAGGLNGLYGSNGALLSGAAGVNGLNGLYGPNGSSASSAGAAGIGGLNGLNGSNGSASAASSSASGSGP
VIVIEENQSSAAAAAASSSSSGQNGLLGPYGPNGSAASSAAASGAAGAGGLNGIYGSNGALLSGAAGVNGLNGLYGPNGSSASSAGAAGIGGLNGLNGSNGSASAASSSASGSRP
VIVIEENQSSAAAAAASSSSSGPNGLLGPYGPNGSSASSAGAAGIGGLNGLNGSNGSSSAAGIGGLNGLNGSNGSSSAASSSASGNGP
VIVIEENQSSAAAAAASSSSSGPNGLLGPYGPNGSSASSAGAAGIGGLNGLNGSNGSASAASSSASGSGP
VIVIEENQSSAAAAAASSSSSGQNGLLGPYGPNGSAASSAAASGAAGAGGLNGLYGSNGALLSGAAGVNGLNGLYGPNGSSASSAGAAGIGGLNGLNGSNGSASAASSSASGSGP
VIVIEENQSSAAAAAASSSSSGQNGLLGPYGPNGSAASSAAASGAAGAGGLNGIYGSNGALLSGAAGVNGLNGLYGPNGSSASSAGAAGIGGLNGLNGSNGSASAASSSASGSRP
VIVIEENQSSAAAAAASSSSSGPNGLLGPYGPNGSSASSAGAAGIGGLNGLNGSNGSSSAAGIGGLNGLNGSNGSSSAASSSASGNGP
VIVIEENQSSAAAAAASSSSSGPNGLLGPYGPNGSSASSAASSGAAGAGGLNGIYGSNGALLSGAAGANGLNGLYGPNGSSASSAGAAGIGGLNGLNGSNGSASAASSNASGSGP
VIVIEENQSSAAAAAASSSSSGLNGPLGPYGPNGSAASSAAASNAGNIGP
VVIIEDNRSSSSAASSSSLGPNGAYGPYGPYGLNGLYGPNGVYGPNGASVTSANAAGVVGLNGLNGSNGSSAAASSSASGSGP
VIVIEENQSSAAAAAASSSSSGPNGLLGPYGPNGSSASSAGAAGIGGLNGLNGSNGSASAASSSASGSGP
VIVIEENQSSAAAAAASSSSSGQNGLLGPYGPNGSAASSAAASGAAGASGLNGIYGSNGALLSGAAGVNGLNGLYGPNGSSASSAGAAGIGGLNGLNGSNGSASAASSSASGSGP
VIVIEENQSSAAAAAASSSSSGQNGLLGPYGPNGSAASSAAASGAAGAGGLNGIYGSNGALLSGAAGVNGLNGLYGPNGSSASSAGAAGIGGLNGLNGSNGSASAASSSASGSGP
VIVIEENQSSAAAAAASSSSSGPNGLLGPYGPNGSSASSAGAAGIGGLNGLNGSNGSSSAAGIGGLNGLNGSNGSSSAASSSASGNGP
VIVIEENQSSAAAAAASSSSSGPNGLLGPYGPNGSSASSAASSGAAGAGGLNGIYGSNGALLSGAAGANGLNGLYGPNGSSASSAGAAGIGGLNGLNGSNGSASAASSNASGSGP
VIVIEENQSSAAAAAASSSSSGLNGPLGPYGPNGSAASSAAASNAGNIGP
VVIIEDNRSSSSAASSSSLGPNGAYGPYGPYGLNGLYGPNGVYGPNGASATSANAAGVVGLNGLNGSNGSSAAASSSASGSGP
VIVIEENQSSAAAAAASSSSSGPNGLLGPYGPNGSSASSAGAAGIGGLNGLNGSNGSASAASSSASGSGP
VIVIEENQSSAAAAAASSSSSGPNGLLGPYGPNGSSASSAASSGAAGASGLNGIYGSNGALLSGAAGANGVNGLYGPNGSSVSSAGAAGIGGLNGLNGLNGSSSAASSSASGSGP
VIVIEENQSSAAAAAASSSSSGQNGLLGPYGPNGSAASSAAASGAAGAGGLNGIYGSNGALLSGAAGVNGLNGLYGPNGSSASSAGAAGIGGLNGLNGSNGSASAASSSASGSGP
VIVIEENQSSAAAAAASSSSSGQNGLLGPYGPNGSSASSAASSGAGNIGP
VVIIEDNGSSSSAASSSSLGPNGAYGPYGPYGLNGLYGPNGVYGPNGASAASASAAGVVGLNGINGSNGSSAAASSSASGSGP
VIVIEENQSSAAAAAASSSSSGPNGLLGPYGPNGSSASSAGAAGIGGLNGLNGLNGSSSAASSSASGSGP
VIVIEENQSSAAAAAASSSSSGPNGLLGPYGPNGSSASSAGAAGIGGLNGLNGLNGSSSAASSSASGSGP
VIVIEENQSSAAAAAASSSSSGPNGLLGPYGPNGSSASSAGAAGIGGLNGLNGSNGSASAASSSASGSGP
VIVIEENQSSAAAAAASSSSSGQNGLLGPYGPNGSSVSSAASSGAGNIGP
VVIIEDNGSSSSAASSSSLGPNGAYGPYGPYGLNGLYGPNGVYGPNGASAASASAAGVVGLNGINGSNGSSAAASSSASGSGP
VIVIEENQSSAAAAAASSSSSGPNGLLGPYGPNGSSASSAGAAGIGGLNGLNGLNGLNGSSSAASSSASGSGP
VIVIEENQSSAAAAAASSSSSGPNGLLGPYGPNGSSALSAGAAGIGGLNGLNGLNGSSSAASSSASGSGP
VIVIEENQSSAAAAAASSSSSGPNGLLGPYGPNGSSASSAGAAGIGGLNGLNGLNGSSSAASSSASGSGP
VIVIEENQLSAAAAAASSSSSGLNGPLGPYGPNGSAASSAAASGAAGEGGLNGLYGSNGALLSGAAGVNGLNGLYGPNGSSASSAGAAGIGGLNGLNGSNGSASAASSSASGSGP
VIVIEENQSSAAAAAASSSSSGQNGLLGPYGPNGSSASSAASSGAGNIGP
VVIIEDNGSSSSAASSSSLGPNGAYGPYGPYGLNGLYGPNGVYGPNGASAASASAAGVVGLNGINGSNGSSAAASSSASGSGP
VIVIEENQSSAAAAAASSSSSGPNGLLGPYGPNGSSASSAGAAGNGGLNGLNGSSSAASSSASGSGP
VIVIEENQSSAAAAAASSSSSGLNVPLGPYGPNGSSASSAASSGAAGAGGLNGIYGSNGALLSGAAGVNGLNGLYGPNGSSASSAGAAGIGGLNGLNGSNGSSSAASSSASGSGP
VIVIEENQSSAAAAAASSSSSGPNGLLGPYGPNGSAASSAAASNAGNNGP
VVIIERGSSSSAAAGSSSSAPYVLPGVRGPCRLTRRQFVVKIGSRRQPCLTC*

(B) *Galleria mellonella* FibH

```
MRVTTFVILCCALQYVTADAIDDSLLNFNNENFIEIGESTTAEVDVENGTLVERETTRKKYERDGDITPNISGEDKIVRTFVIETDASGHETVYEEDVVIKRKPGQQGVTERTTIGRRQTGISAAPVPAPSSQAPTVVVESNSPIAPAPVSGPVSIGPQLGAVGPYGPSRSSTATTTSGTGVVQQIRTDSTGSSAASAATGASSVAP
VIVIEDGSSAASAAAAGSGASGVGGLGLGALGPLGGIGQSGVSSATTSGAGLGGVGAVGASGLGGLGGAGASAAGSAGAGLGGVGVGGSSGSSAASAASGASGAGE
VILIDDRSSAASAAAAGSGASGVGGLGLSGLGPIGGIGPIGATSASTSGAGLGGVGAAGASGLGGLGGAGASAAGSAGAGLGGIGAGGSSGSSAASAASGASGAGE
VIVIDDRSSAASAAAASSGASGLGGLGLGGLGPYGGIGLNGVSSASALGAGLGGVGTAGASGLGGLEGAGVSAVGPAGAGLGGVGAGGSSGSSAASAASGASGPAP
VIVIEDGSSAASAAAAGSGASGLGGLGLGAWGPLGGIGPNGVSSASATGSAAGSTGAGLGGSGAAGSSAASAASGAAGPAP
VIVIEDGSSAASAAAAGSGASGLGGLGLGAWGPLGGIGPNGVSSASATGSAAGSIGSGLGGSGAAGSSAASAASGAAGAAP
VIVIEDGSSAASAAAAGSGASGVGGLGLGALGPLGGIGPIGASSAGASGAGLGGVGAAGTSGLGGIGGVGASTAGSAGAGLGGIGAGGSSGSSAASAASGASGAGE
VIVIDDRSSAASAAAAGSGASGPGGLGLGVWGPLGGIGPIGASSASASGAGLGGVGAAGTSGLGGLGGAGASAAGSAGAGLGGIGASGSSGSSVASAASGTSGAGE
VIVIDDRSSAASAAAAGSGASGLGGLGLGGLGPYGGIGLNGASSASALGAGLGGVGTAGASGLGGLGGAGASAAGSAGAGLGGVGAGGSSGSSVASAASGASGAGE
VIVIDDRSSAASAAAAGSGASGLGGLGLGVWGPLGGIGPNGVSSASALGAGLGGVGTTGASGLGGLGGAGASAAGSAGAGLGGWSSGSSGSSAASAASGASGAGE
VIVIDDRSSAASAAAAGSGASGPGGLGLGVWGPLGGIGPNGVSSASATGSAAGSTGAGLGGSGAAGSSAASAASGAAGPAP
VIVIEDGSSAASAAAAGSGASGLGGLGLGAWGPLGGIGPNGVSSASATGSAAGSTRAGLGGSGAAGSSAASAASGAAGPAP
VIVIEDGSSAASAAAAGSGASGLGGLGLGAWGPLGGIGPNGVSSASATGSAAGSTGAGLGGSGAAGSSAASAASGAAGPAP
VIVIEDGSSAASAAAAGSGASGVGGLGLSALGPLGGIGPNGVSSASALGAGLGGVGAAGASGLGGLGGAGASAAGSAGAGLGGVGAGGSSGSSAASAASGASGAGE
VIVIDDRSSAASAAAAGSGASGLGGLGLGGLGPYGGIGLNGVSSASALGAGLGGVGTAGASGLGGLGGTGASAAGSAGAGLGGVGAGGSSGSSAASAASGASGAGE
VIVIDDRSSAASAAAAGSGASGVGGLGLGAWGPLGGIGPNGVSSASALGAGLGGVGTAGASGLGGLGGTGASAAGSAGAGIGGVGAGGSSGSSAASAASGASGAGE
VIVIDDRSSAASAAAANSGASGPGGLGLGVWGPLGGIGPIGASSASASGAGLGGVGAPGTSGLGGLGGAGASAAGSAGAGLGGIGASGSSGSSAASAASGTSGAGE
VIVIDDRSSAASAAAAGSGASGLGGLGLGGLGPYGGIGLNGVSSASALGAGLGGVGTAGASGLGGLGGAGAPAAGSAGAGLGGVGTGGSSGSSAASAASGASGAGE
VIVIDDRSSAASAAAAGSGASGPGGLGLGVWGPLGGIGPIGASSASASGAGLGGVGATGTSGLGGLGGAGASAAGSAGAGLGGIGAGGSSGSSAASAASGTSGAGE
VIVIDDRSSAASAAAAGSGASGLGGLGLGGLGPYGGIGLNGVSSASALGAGLGGVGTAGASGLGGLGGTGASAAGSAGAGLGGVGAGGSSGSSAASAASGASGAGE
VIVIDDRSSAASAAAAGSGASGVGGLGLGAWGPLGGIGPNGVSSASALGTGLGGVGTTGASGLGGLGGTGASAAGSAGAGLGGVGAGGSSGSSAASAASGASGAGE
VIVIDDRSSAASAAAADSGASGVGGLGLGAWGPLGGIGPNGVSSASALGTGLGGVGTTGASGLGGLGGAGASAAGSAGAGLGGVGTGGSSGSSAASAASGASGAGE
VIVIDDRSSAASAAAAGSGASGLGGLGLGGLGPYGGIGLNGVSSANALGAGLGGLGTSGASGLGGLGGAGTSAAGSVGAGLGGVGRSGAAGSSSASAASGTAGPAP
VIVIEDGSSAASAAAGSGASGLGGLGLGAWGPLGGIGPNGVSSASATGSTAGSSGAGLGGSGAAGSSAASAASGASGPAP
VIVIEDGSSAASAAAAGSGASGLGGLGLGAWGPLGGIGPNGVSSASATGSAAGSTGAGLGGSGAAGSSAASAASGAAGPAP
VIVIEDGSSAASAAAAGSGASGVGGLGLGALGPLGGIGPIGASSAGASGAGLGGVGAAGASGLGGLGGAGASAAGSAGAGLGGVGSGGSSGSSAASAASGASGAGE
VIVINDRSSAASAAAAGSGASGLGGLGLGGLGPYGGIGLNGVSSASALGAGLGGVGTAGASGLGGLGGTGASAAGSAGAGLGGVGAGGSSGSSAASAASGASGAGE
VIVIDDRSSAASAAAAGSGASGVGGLGLGAWGPLGGIGPIGASSASALGAGLGGVGAAGASGLGGLGGAGASAAGSAGAGLGGVGAGGSSGSSAASAASGASGAGE
VIVIDDRSSAASAAAAGSGASGVGGLGLGAWGPLGGIGPNGLSSASALGAGLGGVGNAGASGLGGLGGVGASAAGSAGAGLGGVGTGGSSGSSAASAASGVSGAGE
VIVIDDRSSAASAAAGSGASGPGGLGLGVWGPLGGIGPIGASSASASGAGLGGVGAAGASGLGGIGGAGASAAGSAGAGLGGVGAGGSSGSSAASAASGASGAGE
VIVIDDRSSAASAAAAGSGASGVGGLGLGAWGPLGGIGPNGVSSASALGAGLGGVGNAGASGLGGLGGVGASAAGSAGAGLGGVGTGGSSGSSAASAASGVSGAGE
VIVIDDRSSAASAAAAGSGASGPGGLGLGVWGPLGGIGPIGASSASASGAGLGGVGAAGASGLGGIGGAGASAAGSAGAGLGGVGAGGSSGSSAASAASGASGAGE
VIVIDDRSSAASAAAAASGASGLDGLGLGGLGPYGGIGLNGVSSASALGAGLGGIGASGASGLGGIGGAGASAAGSAGAGIGGVGVGGSSGSSAASAASDAAGPAP
VIVIEDGSSAASAAAAGSGASGPGGLGLGAWGPLGGIGPNGVSSASATGSTAGSTGAGLGGSGAAGSSAASAASGASGPAP
VIVIEDGSSAASAAAAGSGASGLGGLGLGAWGPLGGIGPNGVSSASATGSAAGSTGAGLGGSGAAGSSAASAASGAAGPAP
VIVIEDGSSAASAAAAGSGASGVGGLGLSALGPLGGIGPNGVSSASALGAGLGGVGAAGASGLGGLGGAGASAAGSAGAGLGGVGAGGSSGSSAASAASGASGAGE
VIVIDDRSSAASAAAAASGASGLDGLGLGGLGPYGGIGLNGVSSASALGAGLGGIGASGASGLGGIGGAGASAAGSAGAGIGGVGVGGSSGSSAASAASGAAGPAP
VIVIEDGSSAASAAAAGSGASGPGGLGLGAWGPLGGIGPNGVSSASATGSTAGSTGAGLGGSGAAGSSAASAASGASGPAP
VIVIEDGSSAASAAAAGSGASGLGGLGLGAWGPLGGIGPNGVSSASATGSAAGSTGAGLGGSGAAGSSAASAASGAAGPAP
VIVIEDGSSAASAAAAGSGASGLGGLGLGAWGPLGGIGPNGVSSASATGSAAGSTGAGLGGSSAAGSSAASAASGAAGPAP
VIVIEDGSSAASAAAAGSGASGVGGLGLSALGPLGGIGPNGVSSASALGAGLGGVGAAGASGLGGLGGAGASAAGSAGAGLGGVGAGGSSGSSAASAASGASGAGE
VIVINDRSSAASAAAAGSGASGLGGLGLGGLGPYGGIGLNGVSSASALGAGLGGVGTAGASGLGGLGGTGASAAGSAGAGLGGVGAGGSSGSSAASAASGASGAGE
VIVIDDRSSAASAAAAGSGASGVGGLGLGAWGPLGGIGPNGVSSASALGAGLGGVGTAGASGLGGLGGTGASAAGSAGAGLGGVGTGGSSGSSAASAASGVSGAGE
VIVIDDRSSAASAAAAGSGASGPGLGLGVWGPLGGTGPIGASSASASGAGLGGVGAAGASGLGGIGGAGASAAGSAGAGLGGIGASGSSGSSAASAASGASGAGE
VIVIDDRSSAASAAAAGSGASGLGGLGLGGLGPYGGIGLNGVSSASALGAGLGGIGASGASGLGGIGGAGASAAGSAGARIGGVGVSGSSGSSAASAASGAGGPAP
VIVIEDGSSAASAAAAGSGASGPGGLGLGAWGPLGGIGPNGVSSASATGSTAGSTGAGLGRSGAAGSSAASAASGASGPAP
VIVIEDGSSAASAAAAGSGASGLGGLGLGAWGPLGGIGPNGVSSASATGSAAGSTGAGLGGSGATGSSAASAASGAAGPAP
VIVIEDGSSAASAAAAGSGASGLGGLGLGAWGPLGGIGPNGVSSASATGSAAGSTGAGLGGSGAAGSSAASAASGAAGPAP
VIVIEDGSSAASAAAAGSGASGVGGLGLSALGPLGGIGPNGVSSANALSAGLGGVGAAGASGLGGLGGAGASAAGSAGAGLGGVGAGGSSGSSAASAASGASGAGE
VIVINDRSSAASAAAAGSGASGLGGLGLGGLGPYGGIGLNGVSSASALGAGLGGVGTAGASGLGGLGGTGASSAGSAGAGLGGVGAGGSSGSSAASAASGASGAGE
VIVIDDRSSAASAAAAGSGASGLGGLGLGGLGPYGGIGLNGVSSASALGAGLGGVGTAGASGLGGLGGTGASAAGSAGAGLGGVGAGGSSGSSAASAASGASGAGE
VIVIDDRSSAASAAAAGSGASGVGGLGLGAWGPLGGIGPIGASSASASGAGLGGVGSAGASGLGGLGGAGASAAGSAGAGLGGIGASGSSGSSAASAASGASGAGE
VIVIDDGSSAASAAAAAAGSGASGPGGLGLGVWGPLGGIGPIGASSASASGAGLGGVGAAGASGLGGLGGAGASAAGSAGAGLGGVGAGGSSGSSAASAASGASGAGE
VIVIDDRSSAASAAAAGSGASGLGGLGLGGLGPYGGIGLNGVSSGSALGAGLGGVGTAGASGLGGLGGTGASAAGSAGAGLGGVGAGGSSGSSAASAASGVSGAGE
VIVIDDRSSAASAAAAGSGASGVGGLGLGAWGPLGGIGPNGVSSASATGSTAGSTGAGLGGSGAAGSSAASAASGASGPAP
VIVIEDGSSAASAAAAGSGASGLGGLGLGAWGPLGGIGPNGVSSASATGSAAGSTGAGLGGSGAAGSSAASAASGASGPAP
VIVIEDGSSAASAAAAGSGASGLGGLGLGAWGPLGGIGPNGVSSASATGSTAGSTGAGLGGSGAAGSSAASAASGAAGPTP
VIVIEDGSSAASAAAAGSGASGVGGLGLGSLGPLGGIGQIGASSAGASGAGLGGVGTAGASGLGGLGGAGASAAGSTGAGLGGVGAGGSSGSSAALAASGTSGAGE
VIVIDDRSSAASAAAASSGASGPGGPGLGAWGPLGGFGPIGASSASASGAGLGGVGAAGTSGLGGLGGVGASAAGSAGAGLGGIGAVGSSGSSAASAASGASGAGE
VIVIDDRSSAASAAAAGSGASGPGGLGLGGLGPYGGIGPNVVSAASAPVVGPSVSSVGPVGAQVIEIGSPVVPSVSRTGSVSRVSVSGRPGVRVPCSLTRRQFVVKIGTRRQPCVTC*
```

(C) *Endotricha flammealis* FibH

MRAITFVILCCALQYVTADRVDDNFHQIEKDFNEVSEDTTDVFTKENGTTFEKTTTRKKFERSGDFTPNISGEDKMVTTFVIETDAQGHQTLYEEDVIIKRVADSGATSSASAASSSAAAGAAGGYGRGGQVAYGSNGPNNAYNGAYGTNGASAAPSSSVTSRHSAIGSYGSGSAAAGAASAASSSSQSAP
VVVIEHNDNAAAAAAAAAAAASGSASGSQGAWGSYGGAWAPYGANSASAASAASSSASGASGYVSNDSGSSAAAAAAAAAASSASGSGP
VIVIEDNDSSAAAAAAAAAAAAGSASGSNGAWGQYGPNGQWGPYGSAAAAAAAAAAAAGSSGYGSYGGVYGSNGAAGASGAAAGSASGSSASGVYGAGSNGSGSAAAAAAAAASSASGSGP
VIVIEDNDSSAAAAAAAAAAAAGSASGSNGAWGQYGPNGQWGPYAGASGAAAGSASGSSASGVYGAGSNGSGSAAAAAAAAASSASGSGP
VIVIEDNDSSAAAAAAAAAAAAGSASGSNGAWGQYGPNGQWGPYGSAAAAAAAAAAAAGSSGYGSYGGVYGSNGAAGASGAAAGSASGSSASGVYGAGSNGSGSAAAAAAAAASSASGSGP
VIVIEDNDSSAAAAAAAAAAAAGSASGSNGAWGQYGPNGQWGPYGNAAAAAAAAAAAAGSSGYGSYGGINGSNGAAGASGAAAGSASGSSASGVYGAGSNGSGSAAAAAAAAASSASGSGP
VIVIEDNDSSAAAAAAAAAAAAGSASGSNGAWGQYGPNGQWGPYGSAAAAAAAAAAAAGSSGYGSYGGVYGSNGAAGAGGAAAGSASGSSASVVYGAGSNGSGSAAAAAAAAASSASGSG
VIVIEDNDSSAAAAAAAAAAAAGSASGSNGAWGQYGPNGQWGPYGSAAAAAAAAAAAAGSSGYGSYGGVYGSNGAAGASGAAAGSASGSSASGVYGAGSNGSGSAAAAAAAAASSASGSGP
VIVIEDNDSSAAAAAAAAAAAAGSASGSNGAWGPYGPNGQWGPYGSAAAAAAAAAAAAGSSGYGSYGGDYRSNGAAGASGAAAGSASGSSASGVYGVGSNGSGSAAAAAAAAASSASGSGP
VIVIEDNDSSAAAAAAAAAAAAGSASGSNGAWGQYGPNGQWGPYGSAAAAAAAAAAAAGSSGYGSYGGVYGSNGAAGANGAAAGSASGSSASGVYGAGSNGSGSAAAAAAAAASRASGSGP
VIVIEDNDSSAAAAAAAAAAAAGSASGSNGAWGPYGPNGQWGPYGSAAAAAAAAAAAAGSSGYGSYGGDYGSNGAAGASGAAAGSASGSSASGVYGVGSNGSGSAAAAAAAAASSASGSGP
VIVIEDNDSSAAAAAAAAAAAAGSASGSNGAWGQYGPNGQWGPYGSAAAAAAAAAAAAGSSGYGSYGGVYGSNGAAGASGAAAGSASGSSASGVYGAGSNGSGSAAAAAAAAASSASGSG
VIVIEDNDSSAAAAAAAAAAAAGSASGSNGAWGQYGPNGQWGPYGSAAAAAAAAAAAAGSSGYGSYGGDYGSNGAAGASGAAAGSASGSSASGVYGVGSNGSGSAAAAAAAAASSASGSGP
VIVIEDNDSSAAAAAAAAAAAAGSASGSNGAWGQYGPNGQWGPYGSAAAAAAAAAAAAGSSGYGSYGGVYGSNGAAGASGAAAGSASGSSASGVYGAGSNGSGSAAAAAAAAASSASGSGP
VIVIEDNDSSAAAAAAATAAAGSASGSNGAWGPYGPNGQWGPYGSAAAAAAAAAAAAGSSGYGSYGGDYGSNGAAGASGAAAGSASGSSASGVYGVGSNGSGSAAAAAAAAASSASGSGP
VIVIEDNDSSAAAAAAAAAAAAGSASGSNGAWGQYGPNGQWGPYGSAAAAAAAAAAAAGSSGYGSYGGVYGSNGAAGASGAAAGSASGSSASGVYGAGSNGSGSAAAAAAAAASSASGSGP
VIVIEDNDSSAAAAAAAAAAAAGSASGSNGAWGQYGPNGQWGPYGSAAAAAAAAAAAAGSSGYGSYGGDYGSNGAAGASGAAAGSASGSSASGVYGVGSNGSGSAAAAAAAAASSASGSGP
VIVIEDNDSSAAAAAAAAAAAAGSASGSNGAWGQYGPNGQWGPYGSAAAAAAAAAAAAGSSGYGSYGGVYGSNGAAGASGAAAGSASGSSASGVYGAGSNGSGSAAAAAAAAASSASGSGP
VIVIEDNDSSAAAAAAATAAAGSASGSNGAWGPYGPNGQWGPYGSAAAAAAAAAAAAGSSGYGSYGGDYGSNGAAGASGAAAGSASGSSASGVYGVGSNGSGSAAAAAAAAASSASGSGP
VIVIEDNDSSAAAAAAAAAAAAGSASGSNGAWGQYGPNGQWGPYGSAAAAAAAAAAAAGSSGYGSYGGVYGSNGAAGASGAAAGSASGSSASGVYGAGSNGSGSAAAAAAAAASSASGSG
VIVIEDNDSSAAAAAAAAAAAAGSASGSNGAWGQYGPNGQWGPYGSAAAAAAAAAAAAGSSGYGSYGGVYGSNGAAGASGAAAGSASGSSASGVYGAGSNGSGSAAAAAAAAASSASGSGP
VIVIEDNDSSAAAAAAAAAAAAGSASGSNGAWGPYGPNGQWGPYGSAAAAAAAAAAAAGSSGYGSYGGDYGSNGAAGASGAAAGSASGSSASGVYGVGSNGSGSAAAAAAAAASSASGSGP
VIVIEDNDSSAAAAAAAAAAAAGSASGSNGAWGQYGPNGQWGPNGSAAAAAAAAAAAAGSSGYGSYGGVYGSNGAAGASGAAAGSASGSSASGVYGAGSNGSGSAAAAAAAAASSASGSG
VIVIEDNDSSAAAAAAAAAAAAGSASGSNGAWGQYGPNGQWGPYGSAAAAAAAAAAAAGSSGYGSYGGVYGSNGAAGASGAAAGSASGSSASGVYGAGSNGSGSAAAAAAAAASSASGSGP
VIVIEDNDSSAAAAAAAAAAAAGSASGSNGAWGPYGPNGQWGPYGSAAAAAAAAAAAAGSSGYGSYGGDYGSNGAAGASGAAAGSASGSSASGVYGVGSNGSGSAAAAAAAAASSASGSGP
VIVIEDNDSSAAAAAAAAAAAAGSASGSNGAWGPYGPNGQWGPYGSAAAAAAAAAAAAGSSGYGSYGGDYGSNGAAGASGAAAGSASGSSASGVYGVGSNGSGSAAAAAAAAASSASGSGP
VIVIEDNDSSAAAAAAAAAAAAGSASGSNGAWGPYGPNGQWGPYGSAAAAAAAAAAAAGSSGYGSYGGDYGSNGAAGASGAAAGSASGSSASGVYGVGSNGSGSAAAAAAAAASSASGSGP
VIVIEDNDSSAAAAAAAAAAAAGSASGSNGAWGQYGPNGQWGPYGSAAAAAAAAAAAAGSSGYGSYGGVYGLNGAAGAGGAAAGSASGSSASGVYGAGSNGSGSAAAAAAAAASSASGSG
VIVIEDNDSSAAAAAAAAAAAAGSASGSNGAWGQYGPNGQWGPYGSAVAAAAAAAAAAGSSGYGSYGGVYGSNGAAGASGAAAGSASGSSASGVYGAGSNGSGSAAAAAAAAASSASRSGP
VIVIEDNDSSAAAAAAAAAAAAASSAASGSQGPWGSYGPWGPYGANSASAASAASSAASGASGYVSNDSGSSAAAAAAASAASGASQSAP
VVVIEHNVNAGAAAAAAAAAASASAAGSQGAYPYGGAYPYGGSWGPYGANSASAASAASSAASGASGFVSNDSGSSAAAAAAASAASGASQSAP
VVVIEHNDNAGAAAAAAAAAASASAAGSQGAYPYGGAYPYGGAWGPYGANSASAASAASSAASGASGYVSNDSGSSAAAAAAASAASGASQSAP
VVVIEHNDNAGAAAAAAAAAASASAAGSQGAYPYGGAYPYGGAWGPYGANSASAASAASSAASGASGYVSNDSGSSAAAAAAASAASGASQSAP
VVVIEHNDNAGAAAAAAAAAASASAAGSQGAWPYGGAWGQYGANSASAASAASSAASGSSGYVSNDSGSSAAAAAAAAAASSASGSGP
VIVIEDNDSSDAAAAAAAAAAAAGSASGSNGAWGPYGPNGQWGPYGSASAAAAAAAAAAGSSGYLSYGGVYGSNGAAGASGAAAGSASGSSASGVYGSGSNGSGSAAAAAAAAASSASGSGP
VIVIEDNDSSAAAAAAAAAAAAGSASGSSGAWGPYGPNGQWGPYGSAAAAAAAAAAAAGSSGYGSYGGVYGSNGAAGASGAAAGSASGSSASGVYGAGSNGSGSAAAAAAAAASSASGSGP
VIVIEDNDSSAAAAAAAAAAAAASSAASGSQGPWGSYGPWGPYGANSASAASAASSAASGASGYVSNDSGSSAAAAAAASAASGASQSAP
VVVIEHNDNASAAAAAAAAAASASAAGSQGAYPYGGAYPYGGSWGPYGANSASAASAASSAASGASGYVSNDSGSSAAAAAAASAASGASQSAP
VVVIEHNDNAGAAAAAAAAAASASAAGSQGAYPYGGAYPYGGAWGPYGANSASAASAASSAASGASGYVSNDSGSSAAAAAAASAASGASQSAP
VVVIEHNDNAGAAAAAAAAAASASAAGSQGAYPYGGAYPYGGAWGPYGANSASAASAASSAASGASGYVSNDSGSSAAAAAAASAASGASQSAP
VVVIEHNDNAGAAAAAAAAAASASAAGSQGAWPYGGAWGQYGANSASAASAASSAASGSSGYVSNDSGSSAAAAAAAAAASSASGSGP
VIVIEDNDSSDAAAAAAAAAAAAGSASGSNGAWGPYGPNGQWGPYGSASAAAAAAAAAAGSSGYGSYGGVYGSNGAAGASGAAAGSASGSSASGVYGSGSNGSGSAAAAAAAAASSASGSGP
VIVIEDNDSSAAAAAAAAAAAAGSASGSSGAWGPYGPNGQWGPYGSSAAAAAAAAAAAGSSGYGSYGGVYGSNGAAGASGAAAGSASGSSASGVYGAGSNGSGSAAAAAAAAASSASGSGP
VIVIEDNDSSAAAAAAAAAAAAASSAASGSQGPWGSYGPWGPYGANSASAASAASSAASGASGYVSNDSGSSAAAAAAASAASGASQSAP
VVVIEHNDNAGAAAAAAAAAASASAAGSQGAYPYGGAYPYGGAWGPYGANSASAASAASSAASGASGYVSNDSGSSAAAAAAASAASGASQSAP
VIVIEHNDNASAAAAAAAAAASASAAGSQGAYPYGGAWGPYGANSASAASAASSAASGASGYVSNDSGSSAAAAAAASAASGASQSAP
VVVIEHNDNAGAAAAAAAAAASASAAGSQGAYPYGGAYPYGGAWGPYGANSASAASAASSAASGASGYVSNDSGSSAAAAAAASAASGASQSAP
VVVIEHNDNAGAAAAAAAASASAAGSQGAYPYGGAYPYGGAWGPYGANSASAASAASSAASGASGYVSNDSGSSAAAAAAAAAASSASGSGP
VIVIEDNDSSAAAAAAAAAAAAGSSSGSNGAWGPYGPNGQWGPYGSAAAAAAAAAAAAAAGSSGYGSYGGVYGSNGAAGASGAAAGSASGSSSGVYGVGSNGSGSAAAAAAAAASSASGSGP
VIVIEDNDSSAAAAAAAAAAAAGSASGSNGAWGPYGPNGQWGPYGSAAAAAAAAAAAAGSSGYGSYGGVYGSNGAAGASGAAAGSASGSSASGVYGVGSNGSGSAAAAAAAAASSASGSGP
VIVIEDNDSSAAAAAAAAAAAAGSASGSNGSWGPYGPNGQWGPYGSAAAAAAAAAAAAGSSGYGSYGGDYGSNGAAGASGAAAGSASGSSASGVYGAGSNGSGSAAAAAAAAASSASGSGP
VIVIEDNDSSAAAAAAAAAAAAGSASGSNGAWGPYGPNGQWGPYGSASAAAAAAAAAAGSSGYGSYGGLYGSNGAAGASGAAAGSASGSSASGVYGVGSNGSGSAAAAAAAAASSASGSGP
VIVIEDNDSSAAAAAAAAAAAAGSASGSNGAWGPYGPNGQWGPYGSAAAAAAAAAAAAGSSGYGSYGGNYGSNGAAGASGAAAGSASGSSASGVYGAGSNGSGSAAAAAAAAASSASGSGP
VIVIEDNDSSAAAAAAAAAAAAGSASGSNGSWGPYGPNGQWGPYGSAAAAAAAAAAAAGSSGYGSYGGDYGSNGAAGASGAAAGSASGSSASGVYGAGSNGSGSAAAAAAAAASSASGSGP
VIVIEDNDSSAAAAAAAAAAAAGSASGSNGAWGPYGPNGQWGPYGSASAAAAAAAAAAGSSGYGSYGGLYGSNGAAGASGAAAGSASGSSASGVYGVGSNGSGSAAAAAAAAASSASGSGP
VIVIEDNDSSAAAAAAAAAAAAGSASGSNGAWGPYGPNGQWGPYGSAAAAAAAAAAAAGSSGYGSYGGNYGSNGAAGASGAAAGSASGSSASGVYGAGSNGSGSAAAAAAAAASSASGSGP
VIVIDDNDSSAAAAAAAAAAAAGSASGSNGAWGPYGPNGQWGPYGSASAAAAAAAAAAGSSGYGSYGGDYGSNGAAGASGAAAGSASGSSASGVYGVGSNGSGSAAAAAAAAASSASGSGP
VIVIEDNDSSAAAAAAAAAAAAGSASGSNGSWGPYGPNGQWGPYGSAAAAAAAAAAAAGSSGYGSYGGDYGSNGAAGASGAAAGSASGSSASGVYGAGSNGSGSAAAAAAAAASSASGSGP
VIVIEDNDSSAAAAAAAAAAAAGSASGSNGAWGPYGPNGQWGPYGSASAAAAAAAAAAGSSGYGSYGGLYGSNGAAGASGAAAGSASGSSASGVYGVGSNGSGSAAAAAAAAASSASGSGP
VIVIEDNDSSAAAAAAAAAAAAGSASGSNGAWGPYGPNGQWGPYGSAAAAAAAAAAAAGSSGYGSYGGNYGSNGAAGASGAAAGSASGSSASGVYGAGSNGSGSAAAAAAAAASSASGSGP
VIVIEDNDSSAAAAAAAAAAAAGSASGSNGAWGPYGPNGQWGPYGSAAAAAAAAAAAAGSSGYGSYGGNYGSNGAAGASGAAAGSASGSSASGVYGAGSNGSGSAAAAAAAAASSASGSGP
VIVIDDNDSSAAAAAAAAAAAAGSASGSNGAWGPYGPNGQWGPYGSASAAAAAAAAAAGSSGYGSYGGDYGSNGAAGASGAAAGSASGSSASGVYGVGSNGSGSAAAAAAAAASSASGSGP
VIVIEDNDSSAAAAAAAAAAAAGSASGSNGAWGPYGPNGQWGPYGSASAAAAAAAAAAGSSGYGSYGGLYGSNGAAGASGAAAGSASGSSASGVYGVGSNGSGSAAVAAAAASSASGSGP
VIVIEDNDSSAAAAAAAAAAAAGSASGSNGAWGPYGPNGQWGPYGSAAAAAAAAAAAAGSSGYGSYGGDYGSNGAAGASGAAAGSASGSSASGVYGAGSNGSGSAAAAAAAAASSASGSGP
VIVIEDNDSSAAAAAAAAAAAAGSASGSNGAWGPYGLNGQWGPYGSASAAAAAAAAAAGSSGYGSYGGLYGSNGAAGASGAAAGSASGSSASGVYGVGSNGSGSAAAAAAAAASSASGSGP
VIVIEDNDSSAAAAAAAAAAAAGSASGSNGAWGPYGPNGQWGPYGSAAAAAAAAAAAAGSSGYGSYGGLYGSNGAAGASGAAAGSASGSSASGVYGVGSNGSGSAAAAAAAAASSASGSGP
VIVIEDNDSTAAAAAAAAAAAASSASGSNGAWGPYGPNGQWGPYGSAAAAAAAAAAAAGSSGYGSYGGDYGSNGAAGASGAAAGSASGSSASGVYGAGSNGSGSAAAAAAAAASSASGSGP
VIVIEDNDSSAAAAAAAAAAAAGSASGSNGAWGPYGPNGQWGPYGSASAAAAAAAAAAGSSGYGSYGGLYGSNGAAGASGAAAGSASGSSASGVYGAGSNGSGSAAAAAAAAASSASGSGP
VIVIEDNDSSAAAAAAAAAAAAGSASGSNGAWGPYGPNGQWGPYGSAAAAAAAAAAAAGSSGYGSYGGDYGSNGAAGASGAAAGSASGSSASGVYGDGSNGSGSAAAAAAAAASSASGSGP
VIVIEDNDSSAAAAAAAAAAAAGSASGSNGAWGPYSGAWGPYGANSASAASAASSSASGASGYVSNDSGSSAAAAAAAAAASSASGSGP
VIVIEDNDSSAAAAAAAAAAAAGSASGSNGAWGPYGPNGQWGPYGSAAAAAAAAAAAAGSSGYGSYGGVYGSNGAAGASGAAAGSASGSSASGVYGAGSNGSGSAAAAAAAAASSASGSGP
VIIIEDNNSSAAAAAAAAAAAAAASASGSQGAWGPYGPNVYGQNGYASGSAAGAASASASSASYGRPGVRGACRLTRRQFVVKIGTRRQPCLTC*

(D) *Hypsopygia costalis* FibH

MRATTFVILCCVLQYVAAQYNDGFLNLKGYREVSETNSEDFTTDSNGTLIERITTHKEYKNQGHAPSGLSGNEKIVRTFVIETDADGQETVYEEDVVIKKLPGHSGESTSASSSSSSSASGSGSTGVGPTVVIERHGSAAGAAASSSPSSGNQGPYGPYGPYGPTGAYGANGAAGASSSSGSSASGSGGYGSNGSGSAAAAAAAAASSSEAGP
VVIIEKSGSSAAAAASGSSSSGSQGAWGPYGPNGEWAQYGSSGASAGSSTASGAGGYGAYGPYGPNGAYGSNGAYGANGAAGASSSSGSSASGSSGSGGYGSNGSGSAAAAAAAAASSSGAGP
VVIIEESGSTAAAAAAAASSSSGGYGAYGPYGPNGAYGSNGVYGANGAAGASSSSGSSASGSGGYGSNGSGSAAAAAAAASSSEAGP
VVIIEENGSSAAAAAAAASSSSGGYGAYGPYGPNAAYGSSGAYGANGAAGASSSSGSSASGSGGYGSNGFGSAAAAAAAASSSEAGP
VVIIEESGSSAAAAAAAASSSSGGYGAYGPYGPNSAYGSSGAYGANGAAGASSSSGSSASGSGGYGSNGSGSGAAAAAASSSAAGP
VLIIEESGSSAAAAASGSSSSGSQGVWGPYGPNGEWAQYGSSGASAGSSAASGAGGYGAYGPYGPNGAYGSNGAYGANGAAGASSSSGSSASGSGGYGSNGSGSAAAAAAAASSSEAGP
VVIIEESGSSAAAAAAAASSSSGGYGAYGPYGPNGAYGSSGAYGANGAAGASSSSGSSASGLGGYGSNGSGSAAAAAAAASSSAAGP
VVIIEESGSSAAAAASGSSSSGSQGVWGPYGPNGEWAQYGSSGASAGSSAASGAGGYGAYGPYGPNGAYGSSGAYGANGAAGASSSSSGYSASGSGGYGSNGSGSAAAAAAAASSSEAGP
VVIIEESGSSAAAAAAAASSSSGGYGAYGPYGPNGAYGSSGAYSANGAAGASSSSGSSASGSGGYGSNGSGSAAATAAASSSEAGP
VVIIEESGSSAAAAAAAASSSSGGYGAYGPYGPNGAYGSSGAYGANGAAGASSSSGSSASGSGGYGSNGSGSAAAAAAAASSSGAGP
VVIIEESGSSAAAAASGSSSSGSQGVWGPYGPNGEWAQYGSSGASAGSSAASGEGGYGAYGPYGPNGAYGSSGAYGANGGAGAASSSGSTASGSGGYGSNGSGSAAAAAAAASSSGAGQ
VVIIEESGSSAAAAASGSSSSGSQGVWGPYGPNGEWAQYGSSGASAGSSAASGAGGYGVYGPYGPNGAYGSSGAYGANGAAGASSSSGSSASGSGGYGSNGSGSAAAAAAAASSSGAGP
VVIIEESGSSAAAAASGSSSSGSQGVWGPYGPNGEWAQYGSSGASAGSSAASGEGGYGAYGPYGPNGAYGSSGAYGANGAAGASSSSGSTASGSGGYGSNGSGSGAATAAASSSGNGP
VVIIEESGSSAAAAASGSSSSGSQGVWGPYGPNGEWAQYGSSGASAGSSAASGAGGYGAYGPYGPNGAYGSSGAYGANGAAGASSSSGSSANGSGGYGSNGSGSAAAAAAAASSSGAGQ
VVIIEESGSTAAAAASGSSSSGSQGVWGPYGPNGEWAQYGSSGASAGSSAASGAGGYGAYGPYGPNGAYGSSGAYGANGAAGASSSSSSSASGSDGYGSGGSGSAAAAAAAASSSGAGP
VVIIEESGSSAAAAASGSSSSGSQGVWGPYGPNGEWAQYGSSGASAGSSAASGAGGYGTYGPYGPNGAYGSSGAYGANGAAGASSSSGSSANGSGGYGSNGSGSAAAAAAAASSSGAGP
VVIIEESGSSAAAAASGSSSSGSQGVWGPYGPNGEWAQYGSSGASAGSSAASGAGGYGAYGPYGPNGAYGSSGAYGANGAAGASSSSGSSANGSGGYGSNGSGSAAAAAAAASSSGAGQ
VVIIEESGSSAAAAASGSSSSGSQGVWGPYGPNGEWAQYGSSGASAGSSAASGAGGYGAYGPYGPNGAYGSSGAYGANGAAGASSSSGSTASGSGGYGSNGSGSAAAAAAAASSSGAGP
VVIIEESGSSAAAAASGSSSSGSQGVWGPYGPNGEWAQYGSSGASAGSSAASGAGGYGAYGPYGPNGAYGSSGAYGANGAAGASSSSGSSASGSGGYGSNGSGSAAAAAAAASSSGAGP
VVIIEESGSSAAAAASGSSSSGSQGVWGPYGPNGEWAQYGSSGASAGSSAASGAGGYGAYGPYGPNGAYGSSGAYGANGAAGASSSSSGYSASGSGGYGSNGSGSAAAAAAAASSSEAGP
VVIIEESGSSAAAAAAAASSSSGGYGAYGPYGPNGAYGSSGAYGANGAAGASSSSGSSASGSGGYGSNGSGSGAAAAAASSSAAGP
VLIIEESGSSAAAAASGSSSSGSQGVWGPYGPNGEWAQYGSSGASAGSSAASGAGGYGAYGPYGPNGAYGSNGAYGANGAAGASSSSGSSASGSGGYGSNGSGSAAATAAASSSEAGP
VVIIEESGSSAAAAAAAASSSSGGYGAYGPYGPNGAYGSSGAYGANGAAGASSSSGSSASGSGGYGSNGSGSAAAAAGASSSGAGP
VVIIEESGSSAAAAASGSSSSGSQGVWGPYGPNGEWAQYGSSGASAGSSAASGEGGYGAYGPYGPNGAYGSSGAYGANGAYGANGAAGASSSSGSTASGSGGYGSNGSGSGAATAAASSSGAGP
VVIIEESGSSAAAAASGSSSSGSQGVWGPYGPNGEWAQYGSSGASAGSSAASGAGGYGAYGPYGPNGAYGSSGAYGANGAAGASSSSGSSANGSGGYGSNGSGSAAAAAVASSSGAGP
VVIIEESGSSAAAAASGSSSSGSQGVWGPYGPNGEWAQYGSSGASAGSSAASGAGGYGAFGPYGPNGAYGSSGAYGANGAAGASSSSGSSASGSGGYGSNGSGSAAAAAAAASSSGAGP
VVIIEENGSSAAAAASGSSSSGSQGVWGPYGPNGEWAQYGSSGASAGSSAASGAGGYGAYGPYGPNGAYGSSGAYGANGAAGASSSSGSSASGSGGYGSNGSGSAAAAAAAASSSGAGP
VVIIEESGSSAAAAASGSSSGSQGVWGPYGPNGEWAQYGSSGASAGSSAASGAGGYGAYGPYGPNGAYGSSGAYGANGAAGASSSSGSSASGSGGYGSNGSGSAAVAAAASSSGAGQ
VVIIEENGSSAAAAASGSSSSGSQGAWGPYGPYGPYGPNGAYSSGAAGASSSSSSSASGPNEYGSNESGSAAAAAASAAGSSGAGPSVVIERHGSAAGAAASSSSSSGAQGPWGPYGPYDLYGPNGAYGTSGSAGASSSSSSSASGSDGYGSGGSGSAAAASAASSSGAGP
VVIIEDNGSSAAAAASGSSSSGSQGVWGPYGPNGEWAQYGSSGASAGSASGAGGYGAYGPYGPNGAYGSSGAYGANGAAGASSSSGSSANGSGGYDSNGSGSAAAAAAAASSSGNGP
VVIIEESGSSAAAAASGSSSSGSQGVWGPYGPNGEWAQYGSSGASAGSSAASGSGGYGAYGPYGPNGAYGSSGAYGANGAAGASSSSGSTASGSGGYGSNGSGSAAAAAAAASSSGNGP
VVIIEESGSSAAAAASGSSSSGSQGVWGPYGPNGEWAQYGSSGASAGSSAASGAGGYGAYGPYGPNGAYGSSGAYGANGAAGASSSSSSSASGSDGYGSGGSGSAAAASAASSSGAGP
VVIIEDNGSSAAAAASGSSSSGSQGVWGPYGPNGEWAQYGSSGASAGSSAASGVGGYGAYGPYGPNGAYGSSGAYGANGAAGASSSSGSSASGSGGYGSNGSGSAAAAAAAASSSGAGP
VVIIEESGSSAAAAASGSSSSASQGVWGPYGPNGEWAQYGSSGASAGSSAASGAGGYGAYGPYGPNGAYGSSGAYGANGAAGASSSSGSSASGSGGYGSNGSGSAGAAAAASSSGAGP
VVIIEESGSSAAAAASGSSSSGSQGVWGPYGPNGEWAQYGSSGASAGSSAASGVGGYGAYGPYGPNGAYGSSGAYGANGAAGASSSSGSSASGSGGYGSNGSGSAAAAAAAASSSGAGP
VVIIEESGSSAAAAASGSSSSGSQGAWGPYGPYGPYGPNGAYGSSGAAGASSSSSSSASGPSEYGSYESGSAAAAAASAAGLSGALPSVVIERHGSAAGAAASSSSSSGAQGPWGPYGPYELYGPNGAYGTSGSAGASSSSSSSASGSDGYGSGGSGSAAEASAASSSGAGP
VVIIEESGSSAAAAASGSSSSGSQGVWGPYGPNGEWAQYGSSGASAGSSAASGAGGYGAYGPYGPNGAYGSSGAYGANGAAGASSSSGSSASGSGGYGSNGSGSAAAAAAAASSSAAGP
VVIIEESGSSAAAAASGSSSSGSQGVWGPYGPNGEWAQYGSSGASAGSSAASGAGGYGAYGPYGPNGAYGSSGAYGANGAAGASSSSGSSASGSGGYGSNGSGSAAAAAADSSSAAGP
VVIIEESGSSAAAAASGSSSSGSQGVWGPYGPNGEWAQYGSSGASAGSSAASGAGGYGAYGPYGPNGAYGSSGAYGANGAAGASSSSGSSASGSGGYGSNGSGSAGAAAAASSSGAGP
VVIIEESGSSAAAAASGSSSSGSQGVWGPYGPNGEWAQYGSSGASAGSSAASGAGGYGAYGPYGPNGAYGSSGAYGANGAAGASSSSGSSASGSGGYGSNGSGSAAAAAAAASSAEAGP
VVIIEESGSSAAAAASGSSSSGSQGVWGPYGPNGEWAQYGSSGASAGSSAASGAGGYGAYGPYGPNGAYGSSGAYGANGAAGASSSSGSSASGSGGYGSNGSGSAGAAAAASSSGAGP
VVIIEESGSSAAAAASGSSSSGSQGVWGPYGPNGEWAQYGSSGASAGSSAASGAGGYGAYGPYGPNGAYGSSGAYGANGAAGASSSSGSSASGSGGYGSNGSGSAAAAAAAASSAEAGP
VVIIEESGSSAAAAASGSSSSGSQGVWGPYGPNGEWAQYGSSGASAGSSAASGAGGYGTYGPYGPNGAYGSSGAYGTNGAAGASSSSGSSASGSGGYGSNGSGSAGAAAAASYSGAGP
VVIIEESGSSAAAAASGSSSSGSQGVWGPYGPNGEWAQYGSSGASAGSSAASGAGGYGAYGPYGPNGAYGSSGAYGANGAAGASSSSGSSASGSGGYGSNGSGSAAAAAAAASSAEAGP
VVIIEESGSSAAAAASGSSSSGSQGVWGPYGPNGEWAQYGSSGASAGSSAASGAGGYGTYGPYGPNGAYGSSGAYGTNGAAGASSSSGSSASGSGGYGSNGSGSAAAAAAAASSAEAGP
VVIIEESGSSAAAAASGSSSSGSQGVWGPYGPNGEWAQYGSSGASAGSSAASGAGGYGAYGPYGPNGAYGSSGAYGANGAAGASSSSGSSASGSGGYGSNGSGSAGAAAAASSSGAGP
VVIIEESGSSAAAAASGSSSSGSQGVWGPYGPNGEWAQYGSSGASAGSSAASGAGGYGAYGPYGPNGAYGSSGAYGANGAAGASSSSGSSASGSGGYGSNGSGSAAAAAAAASSSGAGP
VVIIEESGSSAAAAASGSSSSGSQGVWGPYGPNGEWAQYGSSGASAGSSAASGAGGYGAYGPYGPNGAYGSSGAYGANGAAGASSSSGSSASGSGGYGSNGSGSAAAAAAAASSAEAGP
VVIIEESGSSAAAAASGSSSSGSQGVWGPYGPNGEWAQYGSSGASAGSSAASGAGGYGTYGPYGPNGAYGSSGAYGTNGAAGASSSSGSSASGSGGYGSNGSGSAAAAAAAASSAEAGP
VVIIEESGSSAAAAASGSSSSGSQGVWGPYGPNGEWAQYGSSGASAGSSAASGAGGYGAYGPYGPNGAYGSNGAYGSNGAAGASSSSGSSASGSGGYGSNGSGSAAAATAASSSGAGP
VVIIEDSGSSAAAAASSSSSSGSQGVWGPYGPYGSAVAASSSAAAGSDGYGLNGVYGPNGVYGPNEVYGSNGSNGPNVISSSSSPTIIIEEGRSASSSSSSASGTFAPYARPGVRVPCSLTRRQFVVKIGTRRQPCVTC*

(E) *Plodia interpunctella* FibH

MRVTTFVILCCALQYAAADLFGDSLLAGNAREVSESTTDNFTTDGNGNVTEVKTTHKEYRRHGDVPNNISGEDKLVRTFVIETDASGNEVIYEEDVVIHKVPGTSATTKTTTGRRQLGGLGGSSGSAAASSASASRPTVIEKNASSATVAASSSASGPNGQNGPGPIGSSPSSPASSSAAGLNGAYGPYGPNGLYGPNGVYGPNGASAASASAAGANGLNGANGSSASSAAASSASGSGP
VVVIEENSSSVAAAASSNAAGSNGAYGPYGPNGASAASASAAGANGLNGLNGASAASASAAGANGLNGLNGASAASVSAAGVNGLTRLNGANGLNGLNGANGSSASLGSSAVSGASSSGPAVIIERRQGPNASGSAAAFSTSGLNGPYVPIGSSASSAAASSASGNGP
VIIIEEDSSTVAATTSSAAGSNGAYGPYGPNGASAASAGVAGANGLNGANVLNGLNGANGLNGLNGAYGPNGLSASSAAASSASGP
VVIIEEDSSSAAAAAASSSAAGLNGAYGPNGLYGPNGLNGLNGASAASASAAGANGLNGLNGANGSSASSAAASSANGSGP
VVVIEENSSSVAAAASSNAAGSNGAYGPYGPNGASAASASAAGANGLNGLNGLNGASAASASAAGANGLSGLNGASAASASAAGANGLNGLNGASAASANAAGANGLNGLNGPNGSSASSAAASGASGP
VVIIEENSSSAAAAAASSNAARLNGAYGPYGPNGASAASAGVAGANGLNGANVLNGLNGANGLNGLNGAYGPNGLSASSAAASSASGP
VVIIEEDSSSAAAAAASSSAAGLNGAYGPNGLYGPNGLNGLNGASAASASAAGANGLNGLNGANGSSASSAAASSANGSGP
VVVIEENSSSVAAAASSNAAGSNGAYGPYGPNGASAASASAAGANGLNGLNGLNGASAASASAAGANGLSGLNGASAASASAAGANGLNGLNGASAASANAAGANGLNGLNGPNGSSASSAAASSASGP
VVIIEENSSSAAAAAASSNAAGLNGPYGPNGLYGPNGLNGLNGASAASASAAGANGQNGLNGASAASASAAGPNGLNGLNGASAASANAAGANGLNGLNGPNGSSASSAAASSASGP
VVIIEENSSSAAAAAASSNAARLNGAYGPYGPNGASAASASAAGANGLNGLNGASAASASAAGANGLSGLNGASAASASAAGANGLNGLNGASAASANAAGANGLNGLNGPNGSSASSAAASSASGP
VVIIEENSSSAAAAAASSNAAGLNGAYGPYGPNGLYGPNGLNGLNGASAASANAAGANGLNGLNGPNGSSASSAAASGASGP
VVIIEENSSSAAAAAASSNAARLNGAYGPYGPNGASAASASAAGANGLNGLNGVSAASASTAGANGLSGLNGASAASANAAGANGLNGLNGPNGSSASSAAASSASGP
VVIIEENSSSAAAAAASSNAARLNGAYGPYGPNGLYGPNGLNGLNGASAASASAAGANGQNGLNGASAASASAAGANGLNGLNGPNGLSASSATASSASGP
VIIIEENSSSATSNAAGINGAYGPYGPNGAAAASASAAGANGLNGLNGASAASSSAAGANGLNGLNGANGSSASSAAASSASGP
VIIIEENSSSATSNAAGINGAYGPYGPNGATAASASAAGANGLNGLNGASAASASAAGANGLNGLNGASAASANAAGANGLNGLNGQNGSSASSAAASSASGP
VVIIEENSSSAAAAAASSNAAGLNGAYGPYGPNGLYGPNGLNGLNGASAASASAAGANGLSGLNGANGSSASSAAASGASGP
VVIIEENSSSAATAPSSSAAGLNGAYGPYGPNGLYGPNGLYGPNGLNGLNGASAASASAAGANGLNGLNGANGSSASSAAASSASGSGP
VVVIEENSSSVAAAASSNASGSNGAYGPYGPNGASAASASAAGANGLSGLNGASAASASAAGANGLNGLNGASVASANAAGANGLNGLNGPNGSSASSAAASSASGP
VVIIEENSSSAAAAAASSNAAGLNGAYGPYGPNGLYGPNGLNGLNGASAASASAAGANGQNGLNGASSASASAAGANGLNGLNGASAASSSAAGANGLNGLKGPNGSSASSAAASSASGP
VIIIEENSSSATSNAAGINGAYGPYGPNGIYGPNGLNGLNGANGSSASSASSAASGAGGSGP
VVIIEENSSSAATAASSNAAGLNGAYGPYGPNGASVASAGAVGANGLNGLNGASAASASAAGANGLNGLNGASAASANAAGANGLNGLNGPNGSSASSAAASSASGP
VIIIEENSSSATSNAAGINGAYGPYGPNGATAASASAAGANGLNGLNGASAASASAAGANGLNGLNGASAASANAVGANGLNGLNGPNGSSASSAAASSASGP
VVIIEENSSSAAAAAASSNAAGLNGAYGPYGPNGIYGPNGLNGLNGANGSSASSASSAASGAGGSGP
VVIIEENSSSAATAASSNAAGLNGAYGPYGPNGASVASAGAVGANGLNGLNGASAASASAAGANGLNGLNGASAASANAAGANGLNGLNGPNGSSASSAAASSASGP
VIIIEENSSSATSNAAGINGAYGPYGPNGATAASASAAGANGLNGLNGASAASASAAGANGLNGLNGASAASANAAGANGLNGLNGPNGSSASSAAASSASGP
VVIIEENSSSAATAASSSAAGLNGAYGPYGPNGLYGPNGLYGPNGLNGASAASASAAGANGLSGLNGANGSSASSAAASGASGP
VVIIEENSSSAAAAAASSNAAGLNGAYGPYGPNGASVASAGAVGANGLNGLNGASAASASAAGANGLNGLNGASAASASAAGANGLNGLNGASAASANAAGANGLNGLNGPNGSSASSAAASGASGP
VVIIEENSSSAATAASSSAAGLNGAYGPYGPNGLYGPNGLNGASAASASAAGANGLSGLNGANGSSASSAAASSASGP
VVIIEENSSSPAAAASPNAAGLNGAYGPYGPNGLYGPNGLNGLNGASAASASAAGANGLSGLNGSNGSSASSAAASGASGP
VVIIEENSSSAAAAAASSNAAGLNGAYGPYGPNGLYGPNGLNGLNGASAASASAAGANGLSGLNGANGSSASSASSSAASGASGP
VVIIEENSSSAAAAAASSSATGLNGAYGPYGPNGLYGPNGLNGLNGASAASASAAGANGLSGLNGASAASASAAGANGLSGLNGANGSSASSAAASSASGP
VVIIEENSSSPAAAASPNAAGLNGAYGPYGPNGLYGPNGLNGLNGASAASASAAGANGLSGLNGSNGSSASSAAASSASGSGS
VVVIEENSSSVAAAASSNAAGSNGAYGPYGPNGASVASVGAVGANGLNGLNGASAASASAAGANGLNGLNGASAASANAAGANGLNGLNGPNGSSASSAAASGASGP
VVIIEENSSSAAAAAASSNAAGLNGAYGPYGPNGASVASVGAVGANGLNGASAASASAAGANGLNGLNGASAASANAAGANGLNGLNGASAASVSAAGANGLNGLNGASVASANAAGANGLNGLNGPNGSSASSAAASGASGP
IVIIEENSSSAAAAAASSNAAGLNGAYGPYGPNGASVASANAAGANGLNGLNGPNGSSASSAAASSASGLNGLNGASAASANAAGANGLSGLSASGP
VVIIEENSSSAAAAAASSNAAGLNGAYGPYGPNGLYGPNGLNGLNGASAASASAAGANGLSGLNGANGSSASSAAASGASGP
VVIIEENTSSAAAAAASSSAAGLNGAYGPYGPNGLYGPNVLNGLNGASAASASAAGSNGLSGLNGASAASASAAGANGLSGLNGANGSSASSAAASGASGP
VVIIEENSSSAAAAAASSNAAGLNGAYGPYGPNGVSVASAGAVGANGLNGLNGASAASASAAGANGLNGLNGASAASANAAGANGLNGLNGPNGSSASSAAASSASGP
VVIIEENSSSPAAAASPNAAGLNGAYGPYGPNGLYGPNGLNGLNGASAASASAAGANGLSGLNGSNGSSASLAAASGASGP
VVIIEENSSSAAAAAASSNAAGLNGAYGPYGPNGASVASVGAVGANGLNGLNGASAASAGAVGANGLNGLNGASAASASAAGANGQNGLNGASAASANAAGANGLNGLNGPNGSSATSAAASSASGP
VIIIEENSSSAAAAAASSNAAGLNGAYGPYGPNGASVASAGAVGANGLNGLNGASAASASAAGANGLNGLNGASAASANAAGANGLNGLNGPNGSSASSAVASSASGP
VVIIEENSSSAAAAAASSNAAGLNGAYGPYGPNGASVASAGAVGANGLNGLNGASAASASAAGANGLNGLNGASAASANAAGANGLNGLNGPNGSSASSAAASSASGP
VVIIEESSSSAAAAAASSNAAGLNGSYGPYGPNGASVASAGAVGANGLNGASVASAGAVGANGLNGASAASASAAGANGLNGLNGPNGSSASSAAASSASGP
VVIIEENNSSAATATSNAAGLNGAYGPYGPNGATAASASAAGANGLNGLNGASAASSSAAGANGLNGLNGPNGASAASASAAGANGLNGLNALNGANGSSAASSASGP
VVIIEEDSSSAAAAAASSSSGPNGPRGPYGPGAASAALASSAAGNVASNSP
VIIIEEKSSSSAVASSAVAPAPVTPAPADYIYAPSPSPSSSLAAISSAPGP
VVIVEERGSSASASSSSSSSAPYGVPGVRVPCRLTRRQFVVKIGSRRQPCLIC*

(F) *Acrobasis suavella* FibH

MRGPTFVILCCALQYVAADVLDTGLFGTGNVRELKETTTDDYTTDASGNVTEVKTTHKEFERQGQVPNNISGEDKLVRTFVIETDATGHETIYEEDVIIHKVPGTGATTKTTTGRRQIGNLAALASPSSSSAAASSSAASGQGTNRPNGSTASNSAASASSGSNGLNGQGKNQGPAVVIERNRSSGSGDSSSASSSGSQGLNGLNGSNGAYRSYGPNAQYGPNGSSAGASSSAASGPGRVGPNGSSGNNGP
VVVIDEGSSAAAAAAASSSSSGPGGYGSNGPYGPYGPYGPNGSGSSASGSASSGAGGYGPNGQYGPNGSSAGASSSAASGAGGVGPNGSSSAASAAASSSSGNNGPAVVIDEGSSAAAAAAASSSSSGPGGYGSNGPYRPYGPYGPNGSGSSASGAASSGAGGYGPNGQYGPNGSSAGASSSAASGPGGVGPNGSSSAASAAASSSSGKNGP
VVVIDEGSSAAAAAAASSSSSGPGGYGSNGPYGPYGPYGPNGPYGQNGQYGPNGSSAGASSSAASGPGGVGSNGSSSAASAAASSSSGNNGP
VVVIDEGSSAAAAAAASSSSSGPGGYGPNGPYGPYGPYGPNGSGSSASGSASSGAGGYGPNGQYGPNGSSAGASSSAASGPGGVGPNGSSSAASAAASSSSGNNRP
VVVIDEGSSAAAAAAASSSSSGPGGYGSNGPYGPYGPYGPNGPYGQNGQYGPNGSSAGASSSAASGPGGVGPNGSSSAASAAASSSSGNNGP
VVIIDEGSSAAAAAAASSSSSGPGGYGSNGPYGPYGPYGPNGSGSSASGSASSGAGGYGPNGQYGPNGSSAGASSSAASGPGGVGPNGSSSAASAAASSSSGNNGP
VVVIDEGSSAAAAAAASSSSSGPGGYGSNGPYGPYGPYGPNGPYGQNGQYGPNGSSAGASSSAASGPGGVGPNGSSSAASAAASSSSGNNGP
VVVIDEGSSAAAAAAASSSSSGPGGYGSNGPYGPYGPYGPNGSGSSASGSASSGAGGYGPNGQYGPNGSSAGASSSAASGPGGVGPNGSSSAASAAASSSSGNNGP
VVVIDEGSSAAAAAAASSSSSGPGGYGSNGPYGPYGPYGPNGPYGQNGQYGPNGSSAGASSSAASGPGGVGPNGSSSAASAAASSTSGNNGP
VVVIDEGSSSAATAASSSSSGPGGYGPNGPYGPYGPYGPNGSGSSASGAASSGAGGYGPNGQYGPNGSSAGASSSAASGPGGVGPNGSSSAASAAASSSSGNNGP
VVVIDEGSSAAAAAAASSSSSGQGGYGSNGPYGPYGPYGPNGPYGQNGQYGPNGSSAGASSSAASGPGGVGPNGSSSAASAAASSSSGNNGP
VVVIDEGSSAAAATASSSSSGPGGYGPNGPYGPYGPYGPNGSGSSASGSASSGAGGYGPNGQYGPNGSSAGASSSAASGPGGVGPNGSSSAASAAASSSSGNNGP
VVVIDEGSSAAAAAAASSSSSGPGGYGSNGPYGPYGPYGPNGPYGQNGQYGPNGSSAGASSSAASGPGGVGPNGSSSAASAAASSSSGNNGS
VVVIDEGSSAAAAAAASSSSSGPGGYGSNGPYGPYGPYGPNGSGSSASGSASSGAGGYGPNGQYGPNGSSAGASSSAASGPGGVGPNGSSSAASAAASSSSGNNGP
VVVIDEGSSAAAAAAASSSSSGPGGYGSNGPYGPYGPYGPNGPYGQNGQYGPNGSSAGASSSAASGPGGVGPNGSSSAASAAASSTSGNNGP
VVVIDEGSSSAATAASSSSSGPGGYGPNGPYGPYGPYGPNGSGSSASGAASSGAGGYGPNGQYGPNGSSAGASSSATSGPGGVGPNGSSSAASAAASSSSGNNGP
VVVIDEGSSAAAAAAASSSSSGQGGYGSNGPYGPYGPYGPNGPYGQNGQYGPNGSSAGASSSAASGPGGVGPNGSSSAASAAASSSSGNNGP
VVVIDEGSSAAAAAAASSSSSGPGGYGPNGPYGPYGPYGPNGSGSSASGSASSGAGGYGPNGQYGPNGSSAGASSSAASGPGGVGPNGSSSAASAAASSSSGNNGP
VVVIDEGSSAAAAAAASSSSSGPGGYGSNGPYGPYGPYGPNGPYGQNGQYGPNGSSAGASSSAASGPGGVGPNGSSSAASAAASSSSGNNGP
VVVIDEGSSAAAAAAASSSSSGPGGYGSNGPYGPYGPYGPNGSGSSASGSASSGAGGYGPNGQYGPNGSSAGASSSAASGPGGVGPNGSSSAASAAASSSSGNNGP
VVVIDEGSSAAAAAAASSSSSGPGGYGSNGPYGPYGPYGPNGPYGQNGQYGPNGSSAGASSSAASGPGGVGPNGSSSAASAAASSSSGNNGP
VVVIDEGSSAAAAAAASSSSSGPGGYGSNGPYGPYGPYGPNGSGSSASGSASSGAGGYGPNGQYGPNGSSAGASSSAASGPGGVGPNGSSSAASAAASSSSGNNGP
VVVIDEGSSAAAAAAASSSSSGPGGYGSNGPYGPYGPYGPNGPYGQNGQYGPNGSSAGASSSAASGPGGVGPNGSSSAASAAASSTSGNNGP
VVVIDEGSSSAATAASSSSSGPGGYGPNGPYGPYGPYGLNGSGSSASGSASSGAGGYGPNGQYGPNGSSAGASSSAASGPGGVGPNGSSSAASAAASSSSGNNGP
VVVIDEGSSAAAAAAASSSSSGPGGYGSNGPYGPYGPYGPNGPYGQNGQYGPNGSSAGASSSAASGPGGVGPNGSSSAASAAASSSSGNNGP
VVVIDEGSSAAAAAAASSSSSGPGGYGSNGPYGPYGPYGPNGSGSSASGSASSGAGGYGPNGQYGPNGSSAGASSSAASGPGGVGPNGSSSAASAAASSSSGNNGP
VVVIDEGSSAAAAAAASSSSSGPGGYGSNGPYGPYGPYGPNGPYGQNGQYGPNGSSAGASSSAASGPGGVGPNGSSSAASAAASSSSGNNGP
VVVIDEGSSAAAAAAASSSSSGPGGYGPNGPYGPYGPYGPNGSGSSASGSASSGAGGYGPNGQYGPNGSSAGASSSAASGPGGVGPNGSSSAASAAASSSSGNNGP
VVVIDEGSSAAAAAAASSSSSGPGGYGSNGPYGPYGPYGPNGPYGQNGQYGPNGSSAGASSSAASGPGGVGPNSSSSAASAAASSSSGNNGP
VVVIDEGSSAAAAAAASSSSSGPGGYGSNGPYGPYGPYGPNGSGSSASGSVSSGAGGYGPNGQYGPNGSSAGASSSAASGPGGVGPNGSSSAASAAASSSSGNNGP
VVVIDEGSSAAAAAAASSSSSGPGGYGSNGPYGPYGPYGPNGPYGQNGQYGPIGSSAGASSSAASGPGGVGPNGSSSAASAAASSTSGNNGP
VVVIDEGSSSAATAASSSSSGPGGYGPNGPYGPYGPYGPNGSGSSASGAASSGAGGYGPNGQYGPNGSSAGASSSAASGPGGVGPNGSSSAASAAASSSSGNNGP
VVVIDEGSSAAAAAAASSSSSGQGGYGSNGPYGPYGPYGPNGPYGQNGQYGPNGSSAGASSSAASGPGGVGPNGSSSAASAAASSSSGNNGP
VVVIDEGSSAAAAAAASSSSSGPGGYGPNGPYGPYGPYGPNGSGSSASGSASSGAGGYGPNGQYGPNGSSAGASSSAASGPGGVGPNGSSSAASAAASSSSGNNGP
VVVIDEGSSAAAAAAASSSSSGPGGYGSNGPYGPYGPYGPNGPYGQNGQYGPNGSSAGASSSAASGPGGVGPNGSSSAASAAASRSSGNNGP
VVVIDEGSSAAAAAAASSSSSGPGGYGSNGPYGPYGPYGSNGSGSSASGSASSGAGGYGPNGQYGPNGSSAGASSSAASGPGGVGPNGSSSAASAAASSSSGNNGP
VVVIDEGSSAAAAAAASSSSSGPGGYGSNGPYGPYGPYGPNGPYGQNGQYGPNGSSAGASSSAASGPGGVGPNGSSSAASAAASSSSGNNGP
VVVIDEGSSAAAAAAASSSSSGPGGYGPNGPYGPYGPYGPNGSGSSASGSASSGAGGYGPNGQYGPNGSSAGASSSAASGPGGVGPNGSSSAASAAASSSSGNNGP
VVVIDEGSSAAAAAAASSSSSGPGGYGSNGPYGPYGPYGPNGPYGQNGQYGPNGSSAGASSSAASGPGGVGPNSSSSAASAAASSSSGNNGP
VVVIDEGSSAAAAAAASSSSSGPGGYGSNGPYGPYGPYGPNGSGSSASGSVSSGAGGYGPNGQYGPNGSSAGASSSAASGPGGVGPNGSSSAASAAASSSSGNNGP
VVVIDEGSSAAAAAAASSSSSGPGGYGSNGPYGPYGPYGPNGPYGQNGQYGPIGSSAGASSSAASGPGGVGPNGSSSAASAAASSTSGNNGP
VVVIDEGSSSAATAASSSSSGPGGYGPNGPYGPYGPYGPNGSGSSASGAASSGAGGYGPNGQYGPNGSSAGASSSAASGPGGVGPNGSSSAASAAASSSSGNNGP
VVVIDEGSSAAAAAAASSSSSGQGGYGSNGPYGPYGPYGPNGPYGQNGQYGPNGSSAGASSSAASGPGGVGPNGSSSAASAAASSSSGNNGP
VVVIDEGSSAAAAAAASSSSSGPGGYGPNGPYGPYGPYGPNGSGSSASGSASSGAGGYGPNGQYGPNGSSAGASSSAASGPGGVGPNGSSSAASAAASSSSGNNGP
VVVIDEGSSAAAAAAASSSSSGPGGYGSNGPYGPYGPYGPNGPYGQNGQYGPNGSSAGASSSAASGPGGVGPNGSSSAASAAASRSSGNNGP
VVVIDEGSSAAAAAAASSSSSGPGGYGSNGPYGPYGPYGSNGSGSSASGSASSGAGGYGPNGQYGPNGSSAGASSSAASGPGGVGPNGSSSAASAAASSSSGNNGP
VVVIDEGSSAAAAAAASSSSSGPGGYGSNGPYGPYGPYGPNGPYGQNGQYGPNGSSAGASSSAASGPGGVGPNGSSSAASAAASSSSGNNGP
VVVIDEGSSAAAAAAASSSSSGPGGYGSNGPYGPYGPYGPNGSGSSASGSASSGAGGYGPNGQYGPNGSSAGASSSAASGPGGVGPNGSSSAASAAASSSSGNNGP
VVVIDEGSSAAAAAAASSSSSGPGGYGSNGPYGPYGPYGPNGPNGASASSSAASGPGGYRPNGSSASAAASSASASGPGGVNGPNGQSGNQGP
VIIIEEGGSSSSAAALSASSGPGGYGPYGPNGPNGLYGPNEGSAPSPAPVSPGGVNGPNGPSGNQSP
VVIIEKRGPSSAAAASSSSSGPGEYGPNGPYGPNGPYGPNGPYGPNEVSAPSPAPAGPSGVNGPNGPGGNQSP
VVIIEKRGSSSGAAASSSSAGPGGYGPYGPNGPYRPNEVSAPSPAPAGPGVVNGPNGPSGNQSP
VVIIEKRGPSSAAAASSSSSGPGEYGPYGPNGPYGPNGPYGPNEVSAPSPAPAGPGVVNGPNSPSGNQSP
VVIIEEKGSASSAAASSSSAGPGGYGPYGPNGPYGQNGPYGPNGVSASSSAASGPGGYGPNGSSASAAASSASASGPGGVNGPNGPSGNQGP
VIIIEEGDSSSSAAASSLSSGPGGYGPYGPNGPNGPYRPNEVSAPSPAPVSPGGVNGPNGPSGNQSP
VVIIEKRGPSSAAAASSSSSGPGEYGPYGPNGPYGPNGPYGPNEVSAPSPAPAGPSGVNGPNGPGGNQSP
VVIIEKRGSSSGAAASSSSAGPGGYGPYGPNGPYRPNEVSAPSPAPAGPGVVNGPNSPSGNQSP
VVIIEEKGSASSAAASSSSAGPGGYGPYGPNGPYGPYGPNGPYGPNGASASSSAASGPGGYGPNGSSASAAASSASASGPGGVNGPNGPSGNQGP
VIIIEEGDSSSSAAALSASSGPGGYGPYGPNGPNGPYRPNEVSAPSPAPVSPGGVNGPNGPSGNQSP
VVIIEKRGPSSAAAASSSSSGPGEYGPYGPNGPYGPYGPNEVSAPSPAPAGPSDVNGPNGPSENQSP
VVIIEKRGSSSGAAASSSSAGPGGYGPYGPNGPNGPYGPYGPNEVSAPSPAPAGPSDVNGPNGPSENQSP
VVIIEKRGPSSAAAASSSSSGPGGYGPYGPNGASPSSSAASGPGGYGPNGLGSGASSSAASGPGGVNGPSGLSGPGP
VVVIEGGGSSSSASSSSSSYAPYGRPGVRGACRLTRRQFVVKIGTRRQPCSTC*

(G) *Acentria ephemerella* FibH

```
MRVTTFVILFCALQYVTADAIVDSFTKLEKEFHELTETTTDEFDVDKNGTVIERSTTRKKFERDSKAPKQKISGEDKIVRTFVIETDALGHETIYEED
VVIKKVPGQSGSSRKAHSGGRGSSSAASAAGAAAGVSRGPVVIGAPGSRSGAAAAAAAAAASGNDGSYGPNGRRGTRGPGGRGGP
VVIKEGPGAAAAAAAAASAGSGGPYGPYGPGGSRGSVVVGGPGSGSGAAAAATAAAASGNDGSYGPNGRRGSRGPGGRDGL
VVIEDGPGAAAAAAAAASAGSDGPYGPYGPGKSRGPVVVGGPGSGSGAAAAAAAAAASGPDGPYGPGKSRGPVVVGGPGSGSGAAAAAAAAAASGNDGSYGPNGRRGSRGPGGRGGL
VVIEDGPGAAAAAAAAASAGSDGPYGPYGPGKSRGPVVVGGPGSGSGAAAAAAAAAASGPDGPYGPYGPGGSRGPVVVGGPGSGSGAAAAAAAAAASGNDGSYGPNGRRGSRGPGGRGGL
VVIEDGPGAAVAAAAAASAGSDGPYGPYGPGKSRGPVVVGGPGSGSGAAAAAAAAAASGPDGPYGPYGPGKSRGPVVVGGPGSGSGAAAAAAAAAASGNDGSYGPNGRRGSRGPGGRGGL
VVIEDGPGAAAAAAAAASAGSDGPYGPYGPGKSRGPVVVGGPGSGSGAAAAAAAAAASGPDGPYGPYGPGGSRGPVVVGGPGSGSGAAAAAAAAAASGNDGSYGPNGRRGSRGPGGRGGL
VVIEDGPGAAAAAAAAASAGSDGPYGPYGPGKSRGPVVVGGPGSGSGAAAAAAAAAASGPDGPYGPYGPGKSRGPVVVGGPGSGSGAAAAAAAAAASGNDGSYGPNGRRGSRGPGGRGGL
VVIEEGPGAAAAAAAAASAGSDGPYGPYGPGKSRGPVVVGGPGSESGAAAAAAAAAASGPDGPYGPYGPGKSRGPVVVGGPGSGSGAAAAAAAAAASGNDGSYGPNGRRGSRGPGGRGGL
VVIEDGPGAAAAAAAAASAGSDGPYGPYGPGKSRGPVVVGGPGSGSGAAAAAAAAAASGPDGPYGPYGPGGSRGPVVVGGPGSGSGAAAAAAAAAASGNDGSYGPNGRRGSRGPGGRGGL
VVIEDGPGAAAAAAAAASAGSDGPYGPYGPGKSRGPVVVGGPGSGSGAAAAAAAAAASGPDGPYGPYGPGKSRGPVVVGGPGSGSGAAAAAAAAAASGNDGSYGPNGRRGSRGPGGRGGL
VVIEDGPGAAAAAAAAASAGSDGPYGPYGPGKSRGPVVVGGPGSGSGAAAAAAAAAASGPDGPYGPYGPGGSRGPVVVGGPGSGSGAAAAAAAAAASGNDGSYGPNGRRGSRGPGGRGGL
VVIEDGPGAAAAAAAAASAGSDGPYGPYGPGKSRGPVVVGGPGSGSGAAAAAAAAAASGPDGPYGPYGPGGSRGPVVVGGPGSGSGAAAAAAAAAASGNDGSYGPNGRRGSRGPGGRGGL
VVIEDGPGAAAAAAAAASAGSDGPYGNYGPSKSRGPVVVGGPGSGSGAAAAAAAAAASGPDGPYGPYGPGKSRGPVVVGGPGSGSGAAAAAAAAAASGNDGSYGPNGRRGSRGPGGRGGL
VVIEDGPGAAAAAAAAASAGSDGPYGPYGPGKSRGPVVVGGPGSGSGAAAAAAAAAASGPDGPYGPYGPGGSRGPVVVGGPGSGSGAAAAAAAAAASGNDGSYGPNGRRGSRGPGGRGGL
VVIEDGPGAAAAAAAAASAGSDGPYGPYGPGKSRGPVVVGGPGSGSGAAAAAAAAAASGNDGSYGPNGRRGSRGPGGRGGL
VVIEDGPGAAAAAAAAASAGSDGPYGPYGPGKSRGPVVVGGPGSGSGAAAAAAAAAASGPDGPYGPYGPGGSRGPVVVGGPGSGSGAAAAAAAAAASGNDGSYGPNGRRGSRGPGGRGGL
VVIEDGPGAAAAAAAAASAGSDGPYGPYGPGKSRGPVVVGGPGSGSGAAAAAAAAAASGPDGPYGPYGPGGSRGPVVVGGPGSGSGAAAAAAAAAASGNDGSYGPNGRRGSRGPGGRGGL
VVIEDGPGAAAAAAAAASAGSDGPYGPYGPGKSRGPVVVGGPGSGSGAAAAAAAAAASGPDGPYGPYGPGGSRGPVVVGGPGSGSGAAAAAAAAAASGNDGSYGPNGRRGSRGPGGRGGL
VVIEDGPGAAAAAAAAASAGSDGPYGPYGPGKSRGPVVVGGPGSGSGAAAAAAAAAASGPDGPYGPYGPGGSRGPVVVGGPGSGSGAAAAAAAAAASGNDGSYGPNGRRGSRGPGGRGGL
VVIEDGPGAAAAAAAAASAGSDGPYGPYGPGKSRGPVVVGGPGSGSGAAAAAAAAAASGNDGSYGPNGRRGSRGPGGRGGL
VVIEDGPGAAAAAAAAASAGSDGPYGPYGPGKSRGPVVVGGPGSGSGAAAAAAAAAASGPDGPYGPYGPGGSRGPVVVGGPGSGSGAAAAAAAAAASGNDGSYGPNGRRGSRGPGGRGGL
VVIEDGPGAAAAAAAAASAGSDGPYGPYGPGKSRGPVVVGGPGSGSGAAAAAAAAAASGPDGPYGPYGPGGSRGPVVVGGPGSGSGAAAAAAAAAASGNDGSYGPNGRRGSRGPGGRGGV
VVIEEGPGAAASASAAASSSPVVIIEEGSGASAAAAAGASAGSNGPYGPYGPGGPRGPVVGGPGSGSGPYGPNGPFGPNGPLGPNNRGGPRGPLVVGGPGSGSGAAAAAAAAAASGPDGPYGPNGRRGPRGPGGRDGV
VVIEEGPGAAASASAAASSSPVVIIEEGSGASAAAAAGASAGSNGPYGPYGPGGPRGPVVGGPGSGSGPYGPNGPFGPNGPLGPNNRGGPRGPVVVGGPGSGSGAAAAAAAAAASGNDGSYGPNGRRGSRGPGGRGGL
VVIEDGPGAAAAAAAAASAGSDGPYGPYGPGKSRGPVVVGGPGSGSGAAAAAAAAAASGPDGPYGPYGPGGSRGPVVVGGPGSGSGAAAAAAAAAASGNDGSYGPNGRRGSRGPGGRGGL
VVIEDGPGAAAAAAAAASAGSDGPYGPYGPGKSRGPVVVGGPGSGSGAAAAAAAAAASGPDGPYGPYGPGGSRGPVVVGGPGSGSGAAAAAAAAAASGNDGSYGPNGRRGSRGPGGRGGV
VVIEEGPGAAASASAAASSSPVVIIEEGSGASAAAAAGASAGSNGPYGPYGPGGPRGPVVGGPGSGSGPYGPNGPFGPNGPLGPNNRGGPRGPLVVGGPGSGSGAAAAAAAAAASGPDGPYGPNGRRGPRGPGGRDGV
VVIEEGPGAAASASAAASSSPVVIIEEGSGASAAAAAGASAGSNGPYGPYGPGGPRGPVVGGPGSGSGPYGPNGPFGPNGPLGPNNRGGPRGPVVVGGPGSGSGAAAAAAAAAASGPNGPYGPYGPGGSRGPVVVGGPGSGSGAAAAAAAAAASGPDGPYGPNGRRGPRGPGGRDGV
VVIEEGPGAAASASAAASSSPVVIIEEGSGASAAAAAGASAGSNGPYGPYGPGGPLGPVVGGPGSGSGPYGPNGPFGPNGPLGPNNRGGPRGPVVVGGPGSGSGAAAAAAAAAASGPNGPYGPYGPGGSRGPLVVGGPGSGSGAAAAAAAAAASGPDGPYGPNGRRGPRGPGGRDGV
VVIEEGPGAAASASAAASSSPVVIIEEGSGASAAAAAGASAGSNGPYGPNGPLGPNNRGGPLGPVVVGGPGSGSGAAAAAAAAAASGPNGPYGPYGPGGSRGPVVVGGPGSGSGAAAAAAAAAASGPDGPYGPNGRRGPRGPGGRDGV
VVIEEGPGAAASASAAASSSPVVIIEEGSGASAAAAAGASAGSNGPYGPYGPGGPLGPVVGGPGSGSGPYGPNGPFGPNGPLGPNNRGGPRGPVVVAGPGSGSGAAAAAAAAAASGPNGPYGPYGPGGSRGPVVVGGPGSGSGAAAAAAAAAASGPDGPYGPNGRRGPRGPGGRDGV
VVIEEGPGAAASASAAASSSPVVIIEEGSGASAAAAAAAASGPNGPYGPYGPGGSRGPVVVGGPGSGSGAAAAAAAAAASGPNGPYGPYGPGGPRGPVVVGGPGSGSGPYGPNGPFGPNGPLGPNNRGGPRGPVVVAGPGSGSGAAAAAAAAAASGPNGPYGPYGPGGSRGPVVVGGPGSGSGAAAAAAAAAASGPDGPYGPNGRRGPRGPGGRDGV
VVIEEGPGAAASASAAASSSPVVIIEEGSGASAAAAAAAASGPNGPYGPYGPGGSRGPVVVGGPGSGSGAAAAAAAAAASGPNGPYGPYGPGGPRGPVVVGGPGSGSGPYGPNGPSGPNGPYGPYGPGGSRGPVVVGGPGSGSGAAAAAAAAAASGSDGPYGPNGRRGPRGPGGRDGV
VVIEEGPGAAASASAAASSSPVVIIEEGSGASAAAAAGASAGSNGPYGPYGPGGPLGPVVGGPGSGSGPYGPNGPFGPNGPLGPNNRGGPRGPVVVGGPGSGSGAAAAAAAAAASGPNGPYGPYGPGGSRGPVVVGGPGSGSGAAAAAAAAAASGPDGPYGPNGRRGPRGPGGRDGV
VVIEEGPGAAASASAAASSSPVVIIEEGSGASAAAAAAASAGSNGPYGPYGPGGPRGPVVGGPGSGSGPYGPNGPFGPNGPLGPNNRGGPRGPVVVGGPGSGSGAAAAAAAAAASGPNGPYGPYGPGGSRGPVVVGGPGSGSGAAAAAAAAAASGPDGPYGPNGRRGPRGPGGRDGV
VVIEEGPGAAASASAAASSSPVVIIEEGSGASAAAAAAASAGSNGPYGPYGPGGPRGPVVGGPGSGSGPYGPNGPFGPNGPLGPNNRGGPRGPVVVGGPGSGSGAAAAAAAAAASGPNGPYGPYGPGGSRGPVVVGGPGSGSGAAAAAAAAAASGPDGPYGPNGRRGPRGPGGRDGV
VVIEEGPGAAASASAAASSSPVVIIEEGSGASAAAAAAASAGSNGPYGPYGPGGPRGPVVVGGPGSGSGPYGPNGPSGPNGPYGPYGPGGSRGPVVVGGPGSGSGAAAAAAAAAASGPDGPYGPNGRRGPRGPGGRDGV
VVIEEGPGAAASASAAASSSPVVIIEEGSGASAAAAAAASAGSNGPYGPYGPGGPLGPVVGGPGSGSGPYGPNGPFGPNGPLGPNNRGGPRGPVVVGGPGSGSGAAAAAAAAAASGPNGPYGPYGPGGSRGPVVVGGPGSGSGAAAAAAAAAASGPDGPYGPNGRRGPRGPGGRDGV
VVIEEGPGAAASASAAASSSPVVIIEEGSGASAAAAAAASAGSNGPYGPYGPGGPRGPVVVGGPGSGSGPYGPNGPSGPNGPYGPYGPGGSRGPVVVGGPGSGSGAAAAAAAAAASGPDGPYGPNGRRGPRGPGGRDGV
VVIEEGPGAAASASAAASSSPVVIIEEGSGASAAAAAGASAGSNGPYGPYGPGGPLGPVVGGPGSGSGPYGPNGPFGPNGPLGPNNRGGPRGPVVVAGPGSGSGAAAAAAAAAASGPNGPYGPYGPGGSRGPVVVGGPGSGSGAAAAAAAAAASGPDGPYGPNGRRGPRGPGGRDGV
VVIEEGPGAAASASAAASSSPVVIIEEGSGTSAAAAAAASAGSNGPYGPYGPGGPRGPVVVGGPGSGSGPYGPNGPFGPNGPLGPNNRGGPRGPVVVGGPGSGSGAAAAAAAAAASGPDGPYGPNGRRGPRGPGGRDGV
VVIEEGPGAAASASAAASSSPVVIIEEGSGASAAAAAAASAGSNGPYGPYGPGGPRGPVVGGPGSGSGPYGPNGPFGPNGPLGPNNRGGPRGPLVVGGPGSGSGAAAAAAAAAASGPDGPYGPNGRRGPRGPGGRDGV
VVIEEGPGAAASASAAASSSPVVIIEEGSGASAAAAAAASAGSNGPYGPYGPGGSRGPVVVGGPGSGSGAAAAAAATAASGPDGPYGPNGRRGPRGPGGRDGV
VVIEEGPGAAASASAAASSSPVVIIEEGSGASAAAAAGASAGSNGPYGPYGPGGPLGPVVGGPGSGSGPYGPNGPFGPNGPLGPNNRGGSRGPVVVGGPGSGSGAAAAAAAAAASGPNGPYGPYGPGGSRGPVVVGGPGSGSGAAAAAAAAAASGPDGPYGPNGRRGPRGPGGRDGV
VVIEEGPGAAASASAAASSSPVVIIEEGSGASAAAAAAASAGSGGSKYGGYGRPGIRGACRLTRRQFVVKIGTRRQPCLTC*
```

(H) *Cnaphalocrocis (Marasmia) exigua* FibH

MRATTFVILCCALQYAVADDALVDSIRGWEKSFRETNETRTDEFAIDQYGEKIEKITTHKKFERDGTPSGNISGEDKIVRTFVIETDAFGHETIYEEDVVIKKVPGAHHGSSASSAAAAASSAASAASGANAGNNGAGSQSSARANANAAAAAAAAASSRPAGPAGPYGPEGPNGSAAAAAAAASSGPAGPVGQYGPEGPYGPRGPGAAAAAAAAAASSGPAGPVGPYGPEGPYGPEGPYGPRGPGAAAAAAAAAASSGPAGPVGPYGPEGP
NGPYGPNGPLGPNGPLGPNGPLGPNGPLGPNGPEGPYGPEGPYGPRGPGAAAAAAAAAASSGPAGPKGPYGPEGPYGPSGPEGPYGPEGPYGPRGPGAAAAAAAAAASSGPAGPVGPYGPEGPYGPEGPYGLRGPGAAAAAAAAAASSGPAGPVGPYGPEGPNGPYGPNGPLGPNGPLGPNGPLGPNGPFGPNGPYGPEGPYGPEGPYGPRGPGAAAAAAAAAASSGPAGPAGPYGPEGPYGPEGPYGPRGPGAAAAAAAAAASSGPAGPVGP
YGPEGPNGPYGPNGPLGPNGPLGPNGPLGPNGPLGPNGPEGPYGPEGPYGPRGPGAAAAAAAAAASSGPAGPVGPYGPEGPGAAAAAAAAAASSGPAGPVGPYGPEGPNGPYGPNGPIGPNGPYGPEGPYGPEGPYGPRGPGAAAAAAAAAASSGPAGPVGPYGPEGPYGPYGPEGPYGPEGPYGPRGPGAAAAAAAAAASSGPAGPVGPYGPEGPYGQYGPEGPYGPEGPYGPRGPGAAAAAAAAAASSGPAGPVGPYGPEGPYGPEGPYGP
RGPGAAAAAAAAAASSGPAGPVGPYGPEGPGAAAAAAAAAASSGPTGPKGPYGPEGPYGPYGPEGPYGPEGPYGPRGPGAAAAAAAAAASSGPAGPVGPYGPEGPYGPEGPYGPRGPGAAAAAAAAAASSGPAGPVGPYGPEGPNGPYGPNGPLGPNGPLGPNGPLGPNGPFGPNGPYGPEGPYGPEGPYGPRGPGAAAAAAAAAASSGPAGPVGPNGPEGPGAAAAAAAAAASSGPAGPVGPYGSEGPYGPRGPGAAAAAAAAAASSGPAGPVG
PYGPEGPYGPYGPEGPYGPEGPHGPRGPGAAAAAAAAAASSGPAGPVGPYGPEGPNGPYGPNGPLGPNGPLGPNGPLGPNGPFGPYGPEGPYGPEGPYGPEGPYGPRGPGAAAAAAAAAAAAAASSGPAGPVGPYGPEGPNGPYGPNGPLGPNGPLGPNGPLGPNGPFGPNGPYGPEGPYGPEGPYGPEGPYGPRGPGAAAAAAAAAASSGLAGPVGPYGPEGPGAAAAAAAA
ASSGPAGPKGPYGPEGPNGPYGPNGPLGPNGPLGPNGPLGPNGPFGPYGPEGPYGPEGPYGPRGPGAAAAAAAAAASSGPAGPVGPYGPEGPGAAAAAAAAAASSGPAGPVGPYGPEGPNGPYGPNGPLGPNGPLGPNGPLGPNGPFGPYGPEGPYGPRGPGAAAAAAAAAASSGPAGPVGPYGPEGPNGPYGPNGPLGPNGPLGPNGPLGPNGPFGPYGPEGPYGPRGPGAAAAAAAAAASSGPA
GPVGPYGPEGPGAAAAAAAAAASSGPAGPVGPYGPEGPNGPYGPNGPFGPNGPYGPEGPYGPEGPYGPRGPGAAAAAAAAAASSGPAGPVGPYGPEGPYGPYGPEGPYGPEGPYGPSGPGAAAAAAAAAASSGPAGPVGPYGPEGPYGPYGPEGPYGPEGPYGPRGPGAAAAAAAAAASSGPAGPVGPYGPEGPYGPEGPYGPRGPGAAAAAAAAAASSGPAGPVGPYGPEGPGAAAAAAAAAASSGPTGPVGPYGPEGPNGPYGPNGPFGPNG
PYGPEGPYGPEGPYGPRGPGAAAAAAAAAASSGPAGPVGPYGPEGPGAAAAAAAAAASSGPAGPVGPYGPEGPNGPYGPNGPLGPNGPLGPNGPLGPNGPFGPYGPEGPYGPRGPGAAAAAAAAAASSGPAGPVGPYGPEGPGAAAAAAAAAASSGPAGPVGPYGPEGPNGPYGPNGPIGPNGPYGPEGPYGPEGPYGPRGPGAAAAAAAAAASSGPAGPVGPYGPEGPYGPYGPEGPYGPEGPYGPRGPGAAAAAAAAAASSGPAGPVGPYGP
EGPYGPYGPEGPYGPEGPYGPRGPGAAAAAAAAAASSGPAGPVGPYGPEGPYGPEGPYGPRGPGAAAAAAAAAASSGPAGPVGPYGPEGPGAAAAAAAAAASSGPTGPVGPYGPEGPNGPYGPNGPFGPNGPYGPEGPYGPEGPYGPRGPGAAAAAAAAAASSGPAGPVGPYGPEGPYGPYGPEGPYGPEGLYGPRGPGAAAAAAAAAALSGPAGPVGPYGPEGPGAAAAAAAAAA
SSGPAGPVGPYGPEGPNGPYGPNGPLGPNGPLGPNGPLGPNGPFGPYGPEGPYGPEGPYGPRGPGAAAAAAAAAASSGPAGPVGPYGPEGPYGPEGPYGPEGPYGPRGPGAAAAAAAAAASSGPAGPVGPYGPEGPYGPYGPEGPYGPEGPYGPRGPGAAAAAAAAAASSGPAGPVGPYGPEGPNGPYGPNGPFGPNGPYGPEGPYGPRGPGAAAAAAAAAASSGPAGPVGPYGPEGPGAAAAAAAAAASSGPAGPVGPYGPEGPNGPYGPNG
PLGPNGPLGPNGPLGPNGPLGPSGPEGPYGPEGPYGPRGPGAAAAAAAAAASSGPAGPVGPVGPEGPYGPEGPYGPRGPGAAAAAAAAAASSGPAGPVGPYGPEGPGAAAAAAAAAASSGPAGPVGPYGPEGPNGPYGPNGPFGPNGPYGPEGPYGPRGPGAAAAAAAAAASSGPAGPVGPYGPEGPYGPYGPEGPYGPEGPYGPRGPGAAAAAAAAAASSGPAGPVGPYGPEGPNGPYGPNGPLGPNGPLGPNGPLGPNGPFGPNGPFGPEG
PYGPEGPYGPRGPGAAAAAAAAAASSGPAGPVGPYGPEGPYGPYGPEGPYGPEGPYGPRGPGAAAAAAAAAASSGPAGPVGPYGPEGPGAAAAAAAAAASSGPAGPVGPYGPEGPNGPYGPNGPLGPNGPLGPNGPLGPNGPFGPYGPEGPYGPRGPGAAAAAAAAAASSGPAGPVGPYGPEGPYGPEGPYGPRGPGAAAAAAAAAASSGPAGPVGPYGPEGPNGPYGPNGPFGPNGPYGPEGPYGPEGPYGPEGPYGPRGPGAAAAAAAAAAS
SGPAGPVGPYGPEGPNGPYGPNGPLGPNGPLGPNGPLGPNGPFGPNGPYGPEGQYGPEGPYGPRGPGAAAAAAAAAASSGPAGPVGPYGPEGPGAAAAAAAAAASSGPAGPVGPYGPEGPNGPYGPNGPFGPNGPYGPEGPYGPEGPYGPRGPGAAAAAAAAAASSGPAGPVGPYGPEGPYGPYGPEGPYGPEGPYGPRGPGAAAAAAAAAASSGPAGPVGPYGPEGPYGPEGPYGPRGPGAAAAAAAAAASSGPAGPVGPYGPEGPGAAAAA
AAAASSGPAGPVGPYGPEGPYGPYGPEGPYGPEGPYGPRGPGAAAAAAAAAASSGPAGPVGPYGPEGPNGPYGPNGPLGPNGPLGPNGPLGPNGPFGPNGPYGPYGPEGPYGPEGPYGPRGPGAAAAAAAAAASSGPAGPVGPYGPEGPGAAAAAAAAAASSGPAGPVGPYGPEGPNGPYGPNGPLGPNGPLGPNGPLGPNGPFGPYGPEGPYGPRGPGAAAAAAAAAASSGPAGPVGPYGPEGPYGPEGPYGPQGPYGPRGPGAAAAAAAA
ASSGPAGPVGPYGPEGPGAAAAAAAAAASSGPAGPVGPYGPEGPNGPYGPNGPLGPNGPLGPNGPLGPNGPFGPYGPEGPYGPEGPYGPRGPGAAAAAAAAAASSGPAGPVGPYGPEGPYGPEGPYGPEGPYGPRGPEGPYGPEGPYGPRGPGAAAAAAAAAASSGPAGPVGPYGPEGPYGPEGPYGPRGPGAAAAAAAAAASSGPAGPVGPYGPEGPNGPYGPNGPLGPNGPLGPNGPLGPNGPFGPNGPYGPEGPYGPEGPYGPRGPGAA
AAAAAAASSGPAGPVGPYGPEGPGAAAAAAAAAASSGPAGPVGPYGPEGPIGPYGPNGPLGPNGPLGPNGPLGPNGPFGPYGPEGPHGPEGPYGPRGPGAAAAAAAAAASSGPAGPVGPYGPEGPYGPEGPYGPRGPGAAAAAAAAAASSGPAGPVGPYGPEGPYGPEGPYGPRGPGAAAAAAAAAASSGPAGPVGPYGPEGPYGPRGPGAAAAAAAAAASSGPAGPVGPYGPEGPYGPYGPEGPYGPEGPYGPRGPGAAAAAAAAAASSGPAG
PVGPYGPEGPYGPEGPYGPRGPGAAAAAAAAAASSGPAGPYGPYGPEGPYGPNGPMGPNGPNGPEGPYGPEGPYGPRGPGAAAAAAAAAASSGPVGPVGPYGPEGPYGPEGLYGPNGPYGPNGPYGPRGPGSGPAVGPAGPSGPE
VIIIEEQGPGSAAAAAAAASSGPAGPFGPNEPYGPYGPYGPRRPASTAVATATSVSAGPE
VIIIEEQGPRSAAVASASSGPTGPYGPYGPYGPEGPYGPYGPYGPEGPYGPRGPGAAAAAAAASSGPAGPE
VIIIEEQGPSSAAAAAAASSGPVGPAGPYGPYGPYGPEGPYGPRGPGAAAAAAAASSGPAGPE
VIIIEEQGPGSAAAAAAAASGPAGPAGPYGPYGPEGPYGPRGPGSAAAAAAAASSGPAGPVGPEGPYGPNGPLGPNGPLGPNGPLGPNGPLGPKGPLGPNGPNGPYGPEGPYGPEGPYGPRGPGSAAAAAAAASSGPAGPYGPEGPYGPRGPGSAAAAAAAAASGPAGPE
VIIIEEQGPSSAAAAAAASSGPVGPYGPYGPEGPNGPYGPNGPYGPNGPYGPGGPNGPYGPNGPYGPNGPYGPNGPFGPEGPYGPRGPGAAAAAAAASSGPAGPE
VIIIEEQGPSAAAAAAAASSGPYGPNGPYGPNGPYGPGGPNGPYGPNGPYGPNGPYGPNGPYGPRGPGAAAAAAAASSGPAGPE
VIIIEEQGPSAAAAAAAASSGPTGPYGPYGPYGPNGPYGPNGPYGPNGPEGPYGPRGPGSAAAAAAAASSGPAGPYGPEGPYGPEGPYGPRGPGAAAAAAAASSGPAGPE
VIIIEEQGPSSAAAAAAASSGPVGPYGPYGPEGPNGPYGPNGPYGPNGPYGPNGPLGPNGPNGPYGPNGPYGPNGPYGPEGPYGPRGPGAAAAAAAASSGPAGPE
VIIIEEQGPSSAAAAAAASSGPVGPYGPEGPNGPYGPNGPYGPNGPYGPEGPYGPRGPGAAAAAAAASSGPAGPE
VIIIEEQGPSSAAAAAAASSGPVGPYGPYGPYGPYGPEGPYGPRGPGAAAAAAAASSGPAGPE
VIIIEEQGPSSAAAAAAASSGPVGPYGPYGPYGPEGSAASAAAASGSAGPYGPGSAAASAAASASSGAGYGRPGVRTACRLTRRQFVVKIGTRRQPCLTC*

(I) *Chilo suppressalis* FibH

MRVPTFVILCCALQYTAANAINDKIANIEKGLIETDETKTDEFELSKNGTVLEKSITRKKFESENDFQPQFLPKAGEKTVTTLVIETDAEGHQTIYEEDVVIKEVAETSSASSASASSGSRSTAKPLRRRRPYQGFGSRSAASAGAAAASAQRASGVRAAAAAKAEAAAAARAAAPGSAGYGQGGSYGSYGPQVQYGAAGASAAAAAAAAAQASSQTARQGSSSAQSSAQGSAAGAANAAGANSAGYIQGGSYGPQGQYGAAASSAAA
ASAEAAAAAGAAAAAEAAAAAAAAAGSVGYGQGGSYGPQGQYGAAAASAAAAAAAAAQASSQTARQGSSSAQSSAQASAAGAANAAGANSAGYGQGGSYGPQGQYGEAASSAAAASAEAAAAAGATAAAEAAAAAGSVGYGPGGSYGPQGQYGAAAASAAAAAAAAAQASSQNARQGSSSAQGSAQASAAGAANAAGANSAGYRQGGSYGAQGQYGSTAAAAGAAAAAEAAAAASAQRASGAYAGSGSAAAAGAAAAAAAEAEAAAAA
AEAEAAAAARATAAAGSRGYGQGGSYGPQGQFGAAAASAAAAAAAAAQASSQIARQGSSSAQSSAQASAAGAANAAGANSARYGQRGSYGPQGQYGAAAAAAGAAAAAEAAAADSAQRASGAYAGSGSAAAARAAAAAAAEAEAAAAAGSVGYGQGGSYGPYGPQGQYGAAAASAAAAAAAAAQASSQNARQGSASAQGSAQASAAGAANAAGVNSAGYGQGGSYGPQGQHGAAAASAAAASAEAAAAAGAAAAAEAAAAAGSVGYGP
GGSYGPQGQYGAAAASAAAAAAAAAQASSQNARQGSTSAQSSAQASATGAANAAGANSAGYIQGGSYGPQGQYGAAASSAAAASAEAAAAAGAAAAAEAAAAAGSVGYGPGGSYGPQGQYGAAAASAAAAAAAAAQANSQNTRQGSSSAQSSAQASAAGAANAAGANSAGYIQSGSYGPQGQYGAAASSAAAASAEAAAAAGAAAAAEAAAAAGSVGYGPGGSYGPQGQYGAAAASAAAAAAAAAQASSQTARQGSSSAQSSAQASAA
GAANAAGASSAGYRQGGSYGAQGQYGSTAAAAGAAAAAEAAAAASAQRASGAYAGSGSAAAARAAAAAAAEAEAAAAAGSIGYGQGGSYGPYGPQGQYGAAAASAAAAAAAAAQASSQNARQGSSSAQGSAQASAAGAANAAGVNSAGYGQGGSYGPQGQYGAAAASAAAASAEAAAAAGAAAAAEAAAAAGSVGYGPGGSYGPQGQYGAAAASAAAAAAAAAQASSQIARQGSSSAQGSAQASAAGAANAAGANSAGYGQGGSYGPQ
GQYGAAASSAAAASAEAAAAAGAAAAAEAAAAAGSVGYGPGGSYGPQGQYGAAAASAAAAAAAAAQASSQNARQGSSSAQGSAQASAAGAANAAGANSAGYRQEGQYGSTAAAAGAVAAAEAAAAASAQRASGAYAGSGSAAAAGAAAAAAAEAEAAAAAAEAEAAAAAGSVGYGQGGSYGPQGQSGAAAASAAAAAAAAAQASSQIARQGSSSAQGSAQASAAGAANAAGANSARYGQRGSYGPQGQYGPYGPQGQYGAAAASAAAA
AAAAAQASSQNARQGSSSAQGSAQASAAGAANAAGVNSAGYGQGGSYGPQGQYGAAAASAAAASAEAAAAAGAAAAAEAAAAAGSVGYGPGGSYGPQGQYGAAAASAAAAAAAAAQASSQNARQGSSSAQGSAQASAAGAANAAGANSAGYRQGGSYGAQGQYGSLAAAAGAVAAAEAAAAASAQRASGAYAGSGSAAAAGAAAAAAAEAEAAAAAAEAEAEAAAAARATAAAGSVGYGKGGSYGPQGQSGAAAASAAAAAAAAAQAS
SQIARQGSSSAQGSAQASAAGAANAAGANSARYGQRGSYGPQGQYGPYGPQGQYGAAAASAAAQASSQNARQGSSSAQGSAQASAAGAANAAGVNSAGYGQGGSYGPQGQYGAAASSAAAASAEAAAAAGAAAAAEAAAAAGSVGYGPGGSYGPQGQYGAAAASAAAAAAAAAQASSQNARQGSSSAQGSAQASAAGAANAAGANSAGYRQGGSYGAQGQYGSTAAAAGAAAAAEAAAAASAQRASGAYAGSGSAAAAGAAAAAAAEA
EAAAAAAEAEAAAAARATAAAGSVGYGQGGSYGPQGQSGAAAASAAAAAAAAAQASSQIARQGSSSAQGSAQASAAGAANAAGANSARYGQRGSYGPQGQYGPYGPQGQYGAAAASAAAAAAAAAQASSQNARQGSSSAQGSAQASAAGAANAAGVNSAGYGQGGSYGPQGQYGAAAASAAAASAEAAAAAGAAAAAEAAAAAGSVGYGPGGSYGPQGQYGAAAASAAAAAAAAAQASSQNARQGSSSAQGSAQASAAGAANAAGANS
AGYGQGGSYGPQGQYGAAASSAAAASAEAAAAAGAAAAAEAAAAAGSVGYGPGGSYGPQGQYGAAAASAAAAAAAAAQASSQNARQGSSSAQGSAQASAAGAANAAGANSAGYRQGGSYGAQGQYGSTAAAAGAVAAAEAAAAASAQRASGAYAGSGSAAAAGAAAAAAAEAEAAAAAAEAEAAAAARATAAAGSVGYGQGGSYGPQGQSGAAAASAAAAAAAAAQASSQIARQGSSSAQGSAQASAAGAANAAGANSARYGQRGSYG
PQGQYGAAAAAAGAAAAAEAAAAARAQRASGAAGAAAAAAAEAEAAAAAGSVGYGQGGSYEPYGPQGQYGAAAASAAAAAAAAAQASSQNARQGSSSAQGSAQASAAGAANAAGVNSAGYGQGGSYGPQGQYGAAAASAAAASAEAAAAAGAAAAAEAAAAAGSVGYGPGGSYGPQGQYGAAAASAAAAAAAAAQASSQTARQGSSSAQGSAQASAAGAANAAGVNSAGYGPGGSYGSQGQYGAAAASAAAASAEAAAAAGAAAEAEA
AAAAGSVGYGQGGSYGPYGPQGQYGAAAASAAAAAAAAAQASSQNARQGSSSAQGSAQASAAGAANAAGVNSAGYGQGASYGPQGQYGAAAASAAAASADAAAAAGAAAAAEAAAAAGSVGYGPGGSYGPQGQYGAAAASAAAAAAAAAQASSQNARQGSSSAQGSAQASAARAANAAGANSAGYRQGGSYGAQGQYGSTAAAAEATAAASAQRASGAYAGSGSAAAAGAAAAAAAEAEAAAAEAEAAAAARATAAAGSEGYGQGGSY
GPQGQYGAAAASAAAAAAAAAQASSQTARQGSSSAQGSAQASAAGAANAAGVNSAGYGQGGSYGQYGVAAAAGAAAAAEAAAIASAQRASGAYAGSGSAAAAGAAAAAAAEAEAAAAAGSVGYGQGGSYGPYGPQGQYGAAAASAAAAAAAAAQASSQNARQGLASAQGSAQASAAGAANAAGVNSAGYGQGGSYGPQGQYGAAAASAAAASAEAAAAAGAAAAAEAAAAAGSVGYGPGGSYGPQGQYGAAAASAAAAAAAAAQASS
QNARQGSSSAQGSAQASAAGAANAAGVNSAGYGPGGSYGPQGQYGAAAASAAAASAEAAAAAGAAAAAEAAAAAGSVGYGPGGSYGPQGQYGAAAASAAAAAAAAAQASSQSARQGSSSAQGSAQASAAGAANAAGVNSAGYGQGGSYGPQGQYGAAAASAAAAAAAAAQASSQNARQGSSSAQGSAQASAAGAANAAGVNFAGYGPGGSDGPQGQYGAAAASAAAASAEAAAAAGAAAAAEAAAAAGSVGYGPGGSYGPQGQYGAAA
ASAAAAAAAAAQASSQSARQGSSSAQGSAQASAAGAANAAGVNSAGYGQGGSYGQYGAAAAAGAAAAAEAAAAASAQRASGAYAGSGSAAAAGAAAAAAAEAEAAAAAGSVGYGQGGSYGPYGPQGQYGAAAASAAAAAAAAAQASSQNARQGSASAQGSAQASAAGAANAAGVNSAGYGQGGSYGPQGQYGAAAASAAAASAEAAAAAGAAAAAEAAAAAGSVGYGRGGSYGPQGQYGAAAASAAAAAAAAARASSQTARQGSSSAQG
SAQASAAGAANAEGANSAGYGQGGSYGPYGPQGQYGAASASAAAASAAASAQRASGAYAGPGLAAGAGAAAVAEASAAARASAAAGAAGSAGYGQGGSFGSYGQGSSAAAGAAGAEAEAAEAEAAAAEASAAAAAAAASAVALSRSQATKAAAPAPTIIIEESESSTSSSSSASSSESEQINLNGPRSAYKTYRSGSVAAAEASAAATAAAEASRAEAAAAIAESVAAAAEARSQAAAAAEARSQAAAAAAPAPTVIIEENESSTSSST
SFASSSASEEINFYGPGSAGGLYRPGSVAAAEASAAATEASTAAAEASRAAAAAAAAAASAQGATGVRFTRPGVRGPCRLTRRQFVVKIGTRRRPCVGC*

Supplementary Table 1. Summary of primer sequences used in *E. kuehniella* for (A) northern blot probes and (B) real-time qPCR.

(A) Northerns

**Gene**	**Name**	**Forward**	**Reverse**
FibL	Fibroin light chain	TGCTGCCTATCGTTTTGGTATTAT	CGGGCAGCGTTGGGGTCGTT
P25	P25	TGCTACGTTTCTGAGTTCTTCTTC	TGGGACCATAATCTTCACAAT
Ser1A	Sericin 1A	TCCCAGGATGCGAAAATAAATACT	GGCCGCCTGGTTGAATGAT
Ser1B	Sericin 1B	CGACGCCAACGGAAACAC	GCTCTGCGCATTACTTACG
Dana-1	Mucin 1	AGCGCTCAAGCATCTCAATC	GCGCTGCAATGCCAACTTCA
Dana-2	Mucin 1	ATCCGAGGAATCTACGACACTT	
Ser4-1	Ser4	CCCTTGCTTTGGTACTCCTCACG	CCACTGCTACTGCCGCTACTTTGT
Ser4-2	Ser4	CGGCTCCGGCTCAGAAACA	GAGGAGCCATCAGCCGAGACT

(B) qPCR

**Gene**	**Name**	**Forward**	**Reverse**
EF-1α	Elong. factor 1-alpha	TCAAACGGTTACACGCCTGT	GGACTTGGGGTTGTCCTCAG
Fbn	Fibrillin	CACGCTCGTCTCCAACATCT	CACTTCCACCATCCGCATCT
FibH	Fibroin heavy chain	CAACGGACCAGTTGTCATCA	TGTCTACGGGTAAGACGGCA
FibL	Fibroin light chain	AGCCTTGAACAACCGTAGCA	GCGACTGCTCTCAGGAAGTT
Lprcp66-1	rigid cut. protein 66-like 1	CGTTTCAGTCCACCCTGTCA	GCGACAGTAGTAGCGTACCC
Lprcp66-2	rigid cut. protein 66-like 2	AAGTCAAGTCGTCGGCCAAT	CGCCATCCTTCCTCACAACT
Muc1	Mucin 1	ATCATGCACGCCTTGGAAAC	CATTGGCACGAGTTTAGGCG
Muc2	Mucin 2	ACCGAACCATCTACCTCCGA	TTCGGTGGTAGTTTCGGTGG
P25	P25	ACCTTCGCTGACATCACCAC	GTGGAAGTTCTCCCGTTCGT
P47	P47	ATGATGCTGCAGTGGGTTCA	TCTTCACCCACGCCTGTTAG
Pebp	PEBP	CCCGGGAAACTCAACTTCGA	CTGCGAACTTTGCGATGGAG
Pssp1	salivary secr. peptide 1	AATGCCCTGGTTCTGGGTAC	TGTCCTCCCAAAGCCATGAC
Pssp2	Salivary secr. peptide 2	CTTCTCTGACCCTGGACAGC	CGTGATGATGGCTGTGGAGT
Pssp3	Salivary secr. peptide 3	ACCAAGACTTCGTGTACCGC	TAGCGAGGGTACAGCGTACT
Pcp	Pupal cut. protein-like	CTCATGGCAAGGCAATGCTG	CTCGGGGGTTTCCAGGATG

Ser1A	Sericin 1A	AAATCTTGGTCCAGGCACCG	ACTAACGGAAACAGCAATGCT
Ser1B	Sericin 1B	CTCAGTTGCCCCAGGATCTG	TGAAGAAGCAGAGCTGGTGG
Ser3	Sericin 3	ACCACCAAACACAGCCATGA	CTTGATCACCTCTCCGGTCG
SerP150	Sericin P150	CCCAAAGCAGCACCAGTCTT	ATCGAGCTGGACTGTGCATT
Sn1	Seroin 1	GTCGTCAAACGTGAACGGTG	TCCTTGGCATTCTTCGGGTC
Sn2	Seroin 2	TAGTCCTCCCAATCCGAGCA	AGGAGGAGGAGTAGGCTGTG
Sn3	Seroin 3	CCGCTTTCGTTTGTGCTTCA	TTCTCAGTTGATGGACCGGT
Sn4	Seroin 4	TGTGAAGAGCCTCAAGCCAG	CCATGGTCTTCCCGTTCACA
Scp24	Sialomucin core prot. 24	CGCTAAGACACATAGAGACAGACA	TGCGTGTCGTTTCATTGAGG
Spi	Silk proteinase inh.	GGCCGCCATTGTTTCATCAA	TTCCAGAAGCATATCCCGGC
Vsp	Venom serine protease	TACACGCCTGGGAAAGACAC	GTCCATGCCGCAGTAGTCTC
Zon1	Zonadhesin 1	AACGGCTGCGACTGTATTGA	TCCGTTAACGCACGTCGAAT
Zon2	Zonadhesin 2	TACGAAACCAAGACCCTGCC	GTTGACTTCGCAGGTTGGTG
Zon3	Zonadhesin 3	TCGGCAAATTCTTCAAATTCTTCCA	TCCAACAGTCACATTATTGCTGA

Supplementary Table 2. Statistics of expression levels of selected silk genes detected by qPCR in *E. kuehniella*. AMS, anterior-middle SG; MMS, middle-middle SG; RMS, rear-middle SG; PS, posterior SG; PS, anterior-middle SG; WLWS, wandering larva without SG. Statistical differences (T-test; $P < 0.05$) are indicated by asterisk (*).

		**Fold-change**					***P*-value**			
**Gene**	**Name**	**AMS**	**MMS**	**PS**	**RMS**	**WLWS**	**AMS**	**MMS**	**RMS**	**PS**
Fbn	Fibrillin	2,203	0,903	0,768	1,501	1,001	*0.001	0,701	0,155	0,069
FibH	Fibroin heavy chain	2,87	16,685	119,12	17,668	1,023	0,63	*0.011	*0.000	*0.000
FibL	Fibroin light chain	1,125	3,546	29,657	5,481	1,003	0,968	*0.041	*0.004	*0.000
Lprcp66-1	Larval / pupal rigid cuticle protein 66-like 1	2,043	1,611	1,322	1,024	1,132	0,056	0,093	0,826	0,931
Lprcp66-2	Larval / pupal rigid cuticle protein 66-like 2	2,771	1,78	1,038	2,709	1,14	*0.041	*0.048	*0.018	0,242
Muc1	Mucin 1	11,514	31,387	8,811	55,267	1,342	*0.031	*0.010	*0.013	*0.028
Muc2	Mucin 2	0,429	0,513	0,3	0,571	1,036	0,079	0,166	0,25	*0.026
P25	P25	9,418	35,525	14,334	67,263	1,031	*0.001	*0.000	*0.001	*0.003
P47	P47	1,63	2,24	2	4,615	1,009	*0.016	0,168	*0.006	*0.010
Pebp	Phosphatidylethanolamine-bin. Prot	0,499	0,78	1,086	0,678	1,077	0,263	0,586	0,39	0,697
Pssp1	Probable salivary secreted peptide 1	3,879	1,587	1,914	1,823	1,006	*0.000	0,345	0,159	*0.005
Pssp2	Probable salivary secreted peptide 2	3,432	2,106	1,777	6,804	1,054	*0.019	0,082	0,09	0,132
Pssp3	Probable salivary secreted peptide 3	0,114	0,102	0,076	0,079	1,016	*0.003	*0.002	*0.000	*0.001
Pcp	Pupal cuticle protein-like	1,684	4,278	0,573	1,686	1,019	0,568	*0.017	0,241	0,353
Ser1A	Sericin 1A	0,569	2,064	28,324	26,681	1,077	0,229	0,059	*0.007	0,058
Ser1B	Sericin 1B	2,764	7,875	27,865	36,649	1,129	0,1	0,094	*0.008	*0.011
Ser3	Sericin 3	59,288	110,316	12,281	127,197	1,309	*0.025	*0.013	*0.023	0,061
SerP150	Sericin P150	676,983	611,45	39,29	448,051	1,247	*0.005	*0.004	*0.005	*0.012
Sn1	Seroin 1	15,837	14,222	5,167	13,37	1,015	*0.000	*0.001	*0.000	*0.001
Sn2	Seroin 2	2,09	1,746	0,526	1,473	1,009	*0.006	0,051	*0.022	*0.036
Sn3	Seroin 3	4,021	2,294	1,334	1,848	1,038	*0.004	0,09	*0.020	0,341
Sn4	Seroin 4	1,678	1,506	1,083	1,949	1,024	*0.033	0,07	*0.011	0,349
Scp24	Sialomucin core protein 24	7,77	3,925	1,911	5,235	1,231	*0.019	*0.013	*0.005	0,102
Spi	Silk proteinase inhibitor	37,426	83,184	63,73	127,507	1,002	*0.000	*0.000	*0.001	*0.002
Vsp	Venom serine protease-like	4,874	4,942	0,434	1,692	1,01	*0.002	*0.000	0,191	0,469
Zon1	Zonadhesin 1	58,363	85,918	4,94	68,127	1,006	*0.000	*0.000	*0.000	*0.006
Zon2	Zonadhesin 2	16,218	19,306	2,811	18,863	1,173	*0.015	*0.008	*0.008	0,154
Zon3	Zonadhesin 3	106,037	142,901	96,997	205,674	1,028	*0.000	*0.000	*0.000	*0.001

Supplementary Table 3. (A) BUSCO assessment of initial and improved transcriptome of *E. kuehniella.* (B) Genome assembly statistics for *E. kuehniella*.

**(A) *E. kuehniella* transcriptome**	**Initial**	**Improved**
**Element**	Number (%)	Number (%)
Total BUSCO groups searched	1367 (100)	1367 (100)
Complete BUSCOs	1115 (81.6)	1348 (98.6)
Complete and single-copy BUSCOs	771 (56.4)	1341 (98.1)
Complete and duplcated BUSCOs	344 (25.2)	7 (0.5)
Fragmented BUSCOs	110 (8.0)	5 (0.4)
Missing BUSCOs	142 (10.4)	14 (1.0)

**(B) *E. kuehniella* genome assembly**	**Statistics**
Assembly size (Mb)	351,8
Number of contigs	165
Largest contig (Mb)	15,1
GC content (%)	36,1
N50 contig length (Mb)	8,3
Number of protein coding genes	13382
Mean gene length (bp)	7207,8
Repetitive elements (%)	37,8

Supplementary Table 4. Summary of domains identified in zonadhesin protein sequences of *E. kuehniella*, *G. mellonella* and *A. transitella* from family Pyralidae, and *Danaus plexippus* from family Nymphalidae. The search was performed using the web tool MOTIF Search (https://www.genome.jp/tools/motif/) against motif library PROSITE Pattern, PROSITE Profile and Pfam.

**Lepidopteran species**	**GenBank**	**GenPept**	**Name**	**Prosite Pattern**	**Prosite Profile**
Danaus plexippus	XM_032655128.1	XP_032511019	zonadhesin-like C3	EGF_2 x4; SERPIN	CYS_RICH; PROKAR_LIPOPROT.
	XM_032663119.1	XP_032519010	zonadhesin C2	ZINC_FINGER_C2H2_1	
Amyelois transitella	XM_013340097.1	XP_013195551	zonadhesin-like1	EGF_2; ZINC_FINGER_C2H2_1	CYS_RICH
	XM_013336902.1	XP_013192356	zonadhesin-like2	EGF_2 x6; ASX_HYDROXYL x2	CYS_RICH x3; SER_RICH
	XM_013328929.1	XP_013184383	zonadhesin-like3	EGF_2 x3	ANTISTASIN; CYS_RICH x2
	XM_013330525.1	XP_013185979	zonadhesin-like4	EGF_2 x25; serpin	CYS_RICH x2
Galleria mellonella	XM_026900349.2	XP_026756150	zonadhesin-like1 C2	EGF_2 x2; ZINC_FINGER_C2H2_1	CYS_RICH
	XM_031913577.1	XP_031769437	zonadhesin-like2 C3	EGF_2 x6	CYS_RICH x2; SER_RICH
	XM_031914733.1	XP_031770593	zonadhesin-like3 C1	EGF_2 x3	CYS_RICH
	XM_026895687.2	XP_026751488	zonadhesin-like4	EGF_2 x6; SERPIN	CYS_RICH; SER_RICH
	MG770321.1	AXY94923	zonadhesin-like B (ZdB) C3	EGF_2 x5; SERPIN	CYS_RICH
	MG770320.1	AXY94922	zonadhesin-like A (ZdA) C3	EGF_2 x6; SERPIN	CYS_RICH x2; SER_RICH
	NW 022276972.1	XP_026748171.2	uncharacterized protein	EGF_2 x5	
	XM_031913565	XP_031769425	zonadhesin_C2	EGF_2 x6	
Ephestia kuehniella	ON604824		zonadhesin 01	EGF_2 x13	CYS_RICH; SER_RICH
	ON604825		zonadhesin 02	EGF_2 x7; SERPIN	CYS_RICH; SER_RICH
	ON604826		zonadhesin 03	EGF_2 x3	SER_RICH
	OP185494		zonadhesin-like	EGF_2	CYS_RICH

Supplementary Table 5. Summary of proposed landmark genes adjacent to silk genes in *E. kuehniella*, *G. mellonella* and *A. transitella*. These genes are evolutionarily conserved and their positions in the genomes of the three species are shown in Figure 5. The corresponding genes shown in Figure 5 are connected by colored lines (blue, green and magenta).

Color code	Species	Contig / Scaffold	Gene	Name
**Blue**	*Ephestia kuehniella*	contig_172	OP185490	P47 (silk gene)
	Galleria mellonella	NW_022271951.1	LOC113518611 | XP_026759371.2	P47 (silk gene)
	Amyelois transitella	NW_013535448.1	LOC106134372 | XP_013189852.1	P47 (silk gene)
**Green**	*Ephestia kuehniella*	contig_172	EKM1R5v1_00011257	Similar to protein-lysine N-methyltransferase mettl10
		contig_172	EKM1R5v1_00011258	Similar to histidine triad nucleotide-binding protein 3-like
		contig_172	EKM1R5v1_00011259	Similar to motile sperm domain-containing protein 2-like
	Galleria mellonella	NW_022271951.1	LOC113513742 | XP_031767745.1	EEF1A lysine methyltransferase 2
		NW_022271951.1	LOC113513812 | XP_026753595.1	histidine triad nucleotide-binding protein 3-like
		NW_022271951.1	LOC113513811 | XP_026753594.1	motile sperm domain-containing protein 2-like
	Amyelois transitella	NW_013535442.1	LOC106133710 | XP_013188979.1	protein-lysine N-methyltransferase mettl10
		NW_013535442.1	LOC106133711 | XP_013188980.1	histidine triad nucleotide-binding protein 3-like
		NW_013535442.1	LOC106133738 | XP_013189019.1	motile sperm domain-containing protein 2-like
**Magenta**	*Ephestia kuehniella*	contig_493	EKM1R5v1_00013302	Similar to uncharacterized protein LOC106133714
	Galleria mellonella	NW_022271951.1	LOC113513985 | XP_026753770.1	uncharacterized protein LOC113513985
	Amyelois transitella	NW_013535442.1	LOC106133714 | XP_013188983.1	uncharacterized protein LOC106133714
